# Surface Functionalization of Gold Nanoparticles Using Alkyne Derivatives: Applications in Chemical Sensing

**DOI:** 10.1021/acsami.4c12063

**Published:** 2024-10-19

**Authors:** Yun-Qiao Liu, Yi-Cheng Chao, Shun-Qiang Xu, Yun-Rong Peng, Jhih-Jie Syu, Xiang-He Yang, Yung-Kun Pan, Po-Cheng Lin, Ling-Ling Weng, I-Chia Chen, Kui-Thong Tan

**Affiliations:** † Department of Chemistry, National Tsing Hua University, 101 Section 2, Kuang-Fu Road, Hsinchu 300044, Taiwan; ‡ Department of Medicinal and Applied Chemistry, Kaohsiung Medical University, Kaohsiung 80708, Taiwan

**Keywords:** gold nanoparticles, surface functionalization, alkyne, lateral flow assay, chemical sensing

## Abstract

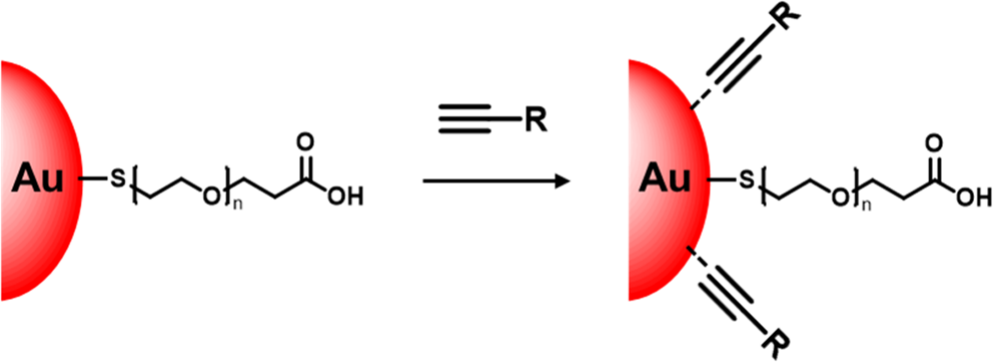

Colloidal gold nanoparticles (AuNPs) are important nanomaterials for chemical sensing and therapeutics. For their application, it is vital to develop a reliable and robust surface functionalization method that can be applied to diverse functional molecules and offer better stability under harsh biological conditions. Currently, thiol (SH) is the most commonly used functional group for forming stable covalent bonds with AuNPs. However, thiolated molecules typically require complicated preparation procedures, are susceptible to oxidation, and are not compatible with many electrophiles and reducing groups. In this study, we report that surface functionalization of AuNPs can be achieved using alkyne derivatives, which exhibit several advantages over classical thiolation and peptide-bond methods, including straightforward preparation of alkyne derivatives, rapid and simple conjugation in buffers and complex media, higher conjugation efficiency, long-term stability, and resistance to decomposition under harsh conditions. Several alkynylated biotin and fluorescein derivatives are prepared, and the alkynylated-AuNPs are characterized using a lateral flow assay, gel electrophoresis, and spectroscopy techniques to investigate the conjugation efficiencies, size distributions, protein interaction properties, and binding mode of the Au–alkyne bond. We also demonstrate that alkynylated-AuNPs can be used for the sensitive detection of hydrogen peroxide and streptavidin proteins.

## Introduction

Gold nanoparticles (AuNPs) are excellent nanomaterials for fabricating novel biochemical sensors and therapeutic reagents owing to their simple preparation procedures, high biocompatibility, chemical inertness, low cytotoxicity, straightforward surface modification, and tunable optoelectronic properties that depend on their shape, size, morphology, and aggregation state.^1−4^ Due to the surface plasmon resonance (SPR) effect, AuNPs display strong absorbance and color, nearly orders of magnitude larger than those of ordinary synthetic organic dyes.^5^ Therefore, many colorimetric sensors and commercial lateral flow assay (LFA) testing kits often use AuNPs as signal reporters to provide simple, rapid, and sensitive detection of diverse classes of target molecules.^6−8^ In addition, AuNPs have been used for detection and therapeutics. For example, AuNPs have been used in photothermal therapy.^9,10^ Furthermore, functionalized AuNPs also play significant roles in the targeted delivery of small-molecule drugs, proteins, and nucleic acids to specific subcellular compartments and cell types in vivo.^11−14^ It is expected that AuNPs will continue to play an important role in chemical sensing and therapeutics.

For the application of AuNPs, it is essential to develop a reliable and robust surface functionalization method. Currently, almost all of the AuNPs are functionalized using either noncovalent physical adsorption or covalent bond formation via the gold–thiol (Au-SH) reaction.^15−17^ Physical adsorption is believed to be established through a collection of interactions, such as hydrogen bonding, electrostatic forces, and hydrophobic forces with the AuNP surface. However, the physical adsorption approach is generally limited to charged macromolecules, thus excluding many small and neutral molecules. Furthermore, the noncovalent reversible AuNP bioconjugates may not be stable under complex intracellular and in vivo conditions or in solutions with high ionic strength and detergents.

In contrast, the covalent conjugation of functional molecules to AuNPs offers increased stability in biological environments and harsh buffering conditions (detergent or high salt concentrations). Diverse molecules, such as small molecules, proteins, synthetic polymers, and oligonucleotides have been reported to form covalent bonds with AuNPs. Currently, thiol (SH) is the most widely used functional group to functionalize AuNP surfaces owing to the rapid formation of a stable Au–S covalent bond.^17−21^ However, there are several restrictions to functionalizing AuNPs using thiolated molecules, including the high chemical reactivity of thiols, complicated synthetic procedures to prepare thiolated functional molecules, and thiols that are susceptible to oxidation and are not compatible with many electrophiles and reducing groups. Furthermore, it has been documented that thiolated-AuNPs exhibit some degree of instability under biologically relevant conditions.^22,23^ Alternatively, the covalent AuNP bioconjugates can also be obtained using two-step procedures. For example, a thiolated poly(ethylene glycol) chain carrying a terminal carboxylic acid is commonly used to cap the AuNP surface to prevent nondesired aggregation and for the reaction with amine derivatives (e.g., polypeptides, nucleic acids, or small molecules) in the presence of 1-ethyl-3-(3-dimethylaminopropyl)carbodiimide/*N*-hydroxysuccinimide (EDC/NHS) peptide-bond coupling reagents.^16^ However, peptide-bond formation on gold nanoparticles using EDC/NHS chemistry typically requires extensive optimization and a large excess of coupling reagents and amine nucleophiles to obtain satisfactory conjugation.^24,25^ Quite often, electrostatic interactions, instead of the desired covalent peptide bonds, are formed between the amine nucleophiles and carboxylic acids of the nanoparticles.

Apart from thiols, derivatives of alkynes,^26−28^ carbenes,^29,30^ phosphines,^31,32^ diazoniums,^33,34^ and selenium^35,36^ have been reported to form covalent conjugates with gold atoms. Of all these functional groups, alkynes have emerged as a promising alternative to thiols due to their small size and relatively inert chemical reactivity that has been used in many applications as reporters and conjugation tags.^37−40^ Alkynes carrying different functional groups (e.g., carboxylic acids, amines, and halogens) are widely available for coupling with diverse functional molecules via simple one-step peptide or alkylation reactions. Therefore, the preparation of alkynylated functional molecules is much simpler than that of the thiol derivatives. Currently, there are only a few reported applications of alkynylated metal nanoparticles that mainly focus on surface-enhanced Raman scattering (SERS) imaging.^41,42^ In those studies, the surfaces of bare gold/silver nanoparticles were modified by alkynes to form irreversible aggregates, which resulted in the functional loss of the nanoparticles for subsequent applications. To date, the scope and applications of alkynylated-AuNPs remain very limited.

**Figure 1:**
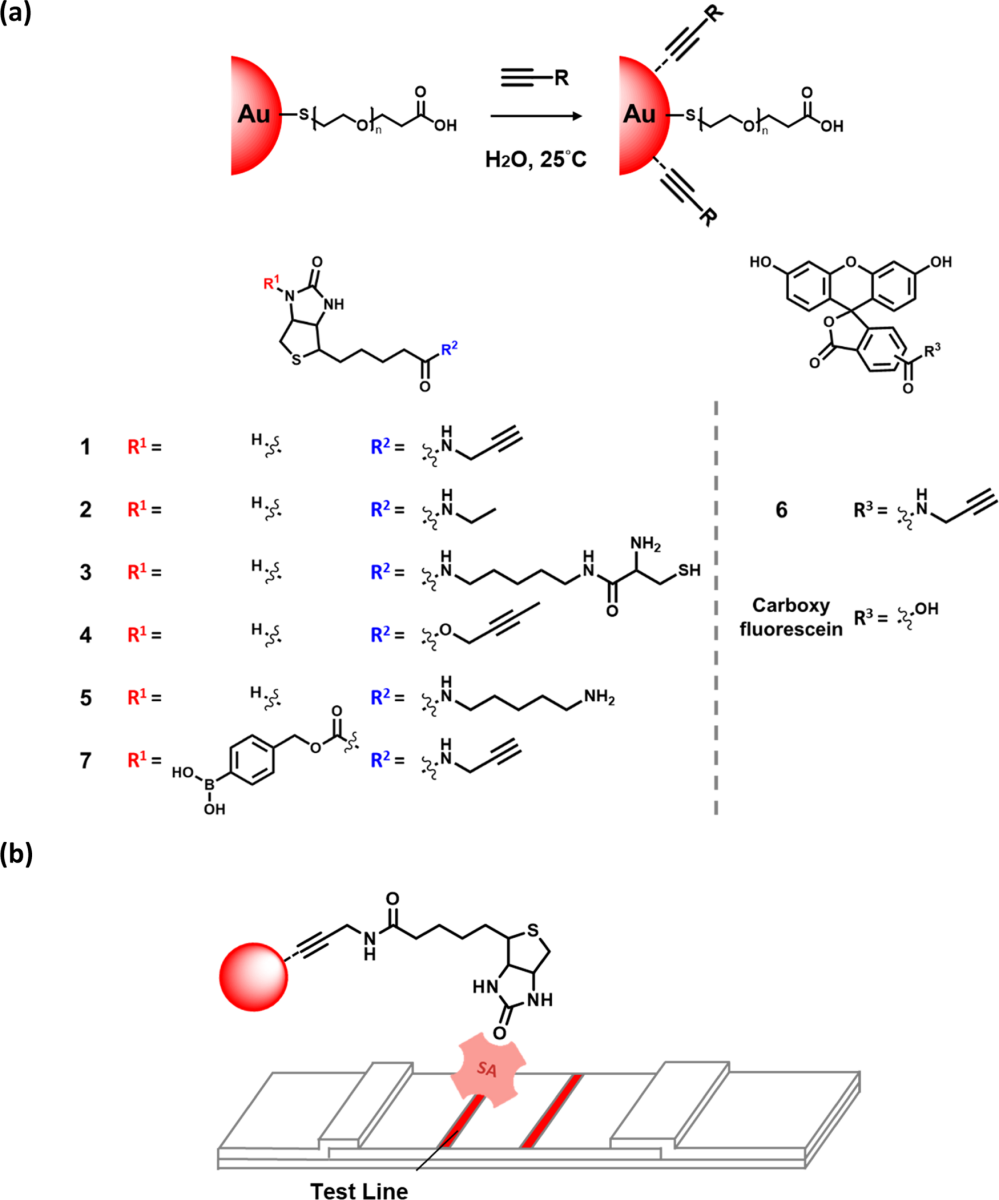
(a) Reaction scheme and chemical structure of alkynylated molecules used for surface functionalization of poly(ethylene glycol) capped-gold nanoparticles **Au@PEG**. (b) Schematic illustration of lateral flow assay (LFA) for analysis of conjugation efficiency. Biotinylated gold nanoparticles can be captured by the immobilized streptavidin protein to form a red color test line. SA: streptavidin protein.

Herein, we report a simple and rapid surface-functionalized approach to generate stable colloidal AuNPs using alkyne derivatives (Figure 1a). In this paper, several biotin and fluorescein derivatives with or without an alkyne group were prepared to understand the alkynylated-functionalized AuNP formation (Schemes S1–S7). Lateral flow assay (LFA), gel electrophoresis, and spectroscopy techniques were used to study the alkynylated-AuNPs. LFA is a simple and low-cost analytical device in which the colorimetric signal on the test line is generated upon molecular interactions of the ligand on the AuNPs and the binding protein immobilized on the nitrocellulose membrane (Figure 1b).^43^ Therefore, LFA can serve as an ideal analytical tool to assess the conjugation efficiency and function of the alkynylated-AuNPs, as biotin ligand can bind very tightly to streptavidin protein (*K*_d_ = 1 × 10^–14^ M) immobilized on the test line. Furthermore, LFA can also be used to study the stability and colloidal states of AuNPs in buffers or complex solutions, as large aggregated nanoparticles are not able to flow through the nitrocellulose membrane. In addition to LFA, agarose gel electrophoresis, absorption and fluorescence spectra, fluorescence lifetime measurements, transmission electron microscopy (TEM) imaging, and dynamic light scattering (DLS) were also used to characterize and validate the colloidal and functionally active alkynylated-AuNPs.

## Results and Discussion

### Surface Functionalization of Gold Nanoparticles (AuNPs) with Alkynylated-Biotin **1**

**Figure 2:**
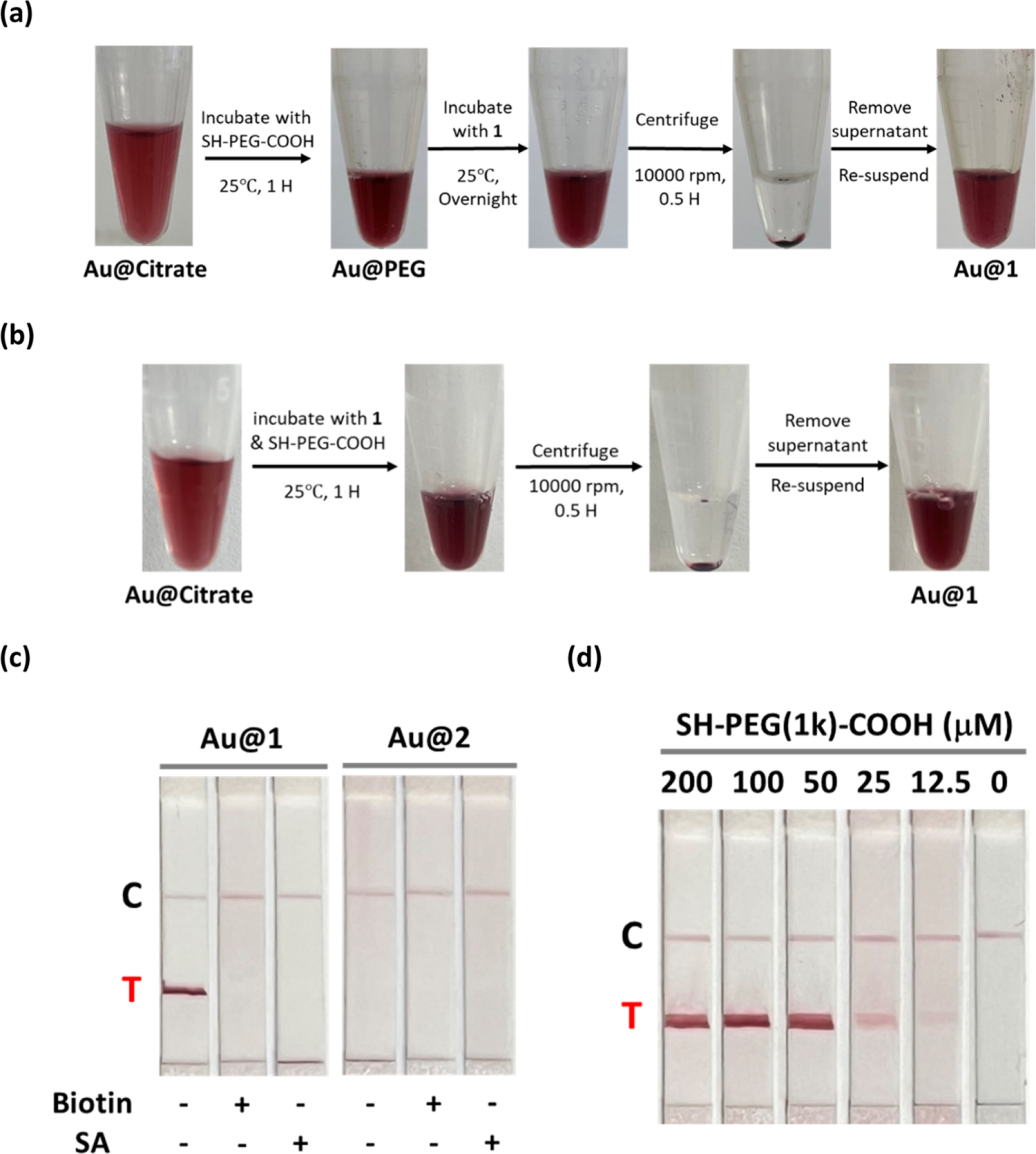
Images and steps for the preparation of alkynylated-gold nanoparticles using (a) two- and (b) one-step procedures. (c) Biotinylation of **Au@1** and **Au@2** analyzed using streptavidin-alkaline phosphatase (SA-ALP)-immobilized LFA test strips in the presence of 0.1 mg/mL streptavidin (SA) or 100 μM biotin. C: control line and T: test line. (d) The reaction of **Au@citrate** with a solution containing a mixture of 25 μM **1** and various concentrations of SH-PEG(1K)-CO_2_H (0–200 μM).

In this article, the Turkevich method was employed to prepare the citrate-capped gold nanoparticles **Au@citrate**. In general, stable and colloidal alkynylated-gold nanoparticles can be obtained via either a simple one- or two-step procedure. For the two-step procedure, **Au@citrate** was capped with SH-PEG(1K)-CO_2_H at 25 °C for 2 h to obtain **Au@PEG**, which was further reacted with alkynylated-biotin **1** to afford the colloidal gold nanoparticle **Au@1** solution in wine red color (Figure 2a). In the one-step method, a solution containing a mixture of **1** and SH-PEG(1K)-CO_2_H was incubated with **Au@citrate** at 25 °C for 1 h to obtain **Au@1** (Figure 2b). It is noteworthy to mention that the one-step procedure provides a rapid and simple way to functionalize AuNPs, whereas the two-step method is suitable for the functional molecules containing electrophilic groups, e.g., alkyl halides, unsaturated alkenes, and epoxides, which are not compatible with the thiolated poly(ethylene glycol). It is crucial to stabilize the gold nanoparticles with poly(ethylene glycol) for the direct incubation of compound **1** and other small molecules with **Au@citrate** changes the solution color from wine red to deep blue, indicating the formation of large and irreversible aggregates (Figure S1). In fact, this is a common phenomenon observed for many nanoparticles that have directly conjugated small molecules on the surface.^44,45^ We also found that the linker length of poly(ethylene glycol) is important, as a shorter chain linker will cause the alkynylated gold nanoparticles to form large aggregates that cannot be detected using LFA test strips (Figure S2).

The conjugation and function of **Au@1** were evaluated using an LFA test strip, with streptavidin immobilized on the test line (T-line) and goat antimouse IgG on the control line (C-line). The test strip was dipped into a Tris buffer solution containing a mixture of **Au@1** and **Au@Control**. We first tested **Au@1** prepared by using a two-step procedure. After 10 min of solution flow, we observed an obvious red color band on the test line (Figure 2c). In contrast, the test line was not visible when 0.1 mg/mL streptavidin protein or 100 μM biotin was added to the solution, indicating specific binding between the biotin of **Au@1** and the immobilized streptavidin. To confirm that the alkyne is critical for the surface functionalization of AuNPs, probe **2**, which does not have an alkyne group was reacted with **Au@PEG** according to the procedure shown in Figure 2a. However, a distinct test line was not detected with the **Au@2**. We also immobilized and compared different variants of streptavidin proteins, of which streptavidin conjugated with alkaline phosphatase (SA-ALP) yielded the strongest signal on the test line (Figure S3). By using **Au@citrate** and **Au@PEG**, which are not functionalized with probe **1**, we did not observe any obvious red band on the test line with the unfunctionalized AuNPs (Figure S4). Therefore, the LFAs performed in this paper were conducted using a two-step procedure and the SA-ALP test line unless otherwise noted.

For **Au@1** prepared using the one-step procedure, a strong **Au@1** test line signal can be obtained even in the presence of an 8-fold excess of SH-PEG(1K)-CO_2_H compared to **1** (Figure 2d). This suggests that the alkyne-gold bond might be stronger than the thiol-gold bond. In fact, it was determined previously by density functional theory calculations that the interaction energies of alkyne-gold and thiol-gold are 109.8 and 72.7 kcal/mol, respectively.^46,47^ The formation of alkynylated-AuNPs was also studied using agarose gel electrophoresis, which shows that **Au@1** has a larger molecular size than **Au@PEG**, while **Au@2** exhibits a similar migration rate as **Au@PEG** (Figure S5). These results indicate that alkynylation is a simple and rapid approach to generating colloidal and functionalized AuNPs.

### Size Distributions of Alkynylated-Gold Nanoparticles

**Figure 3:**
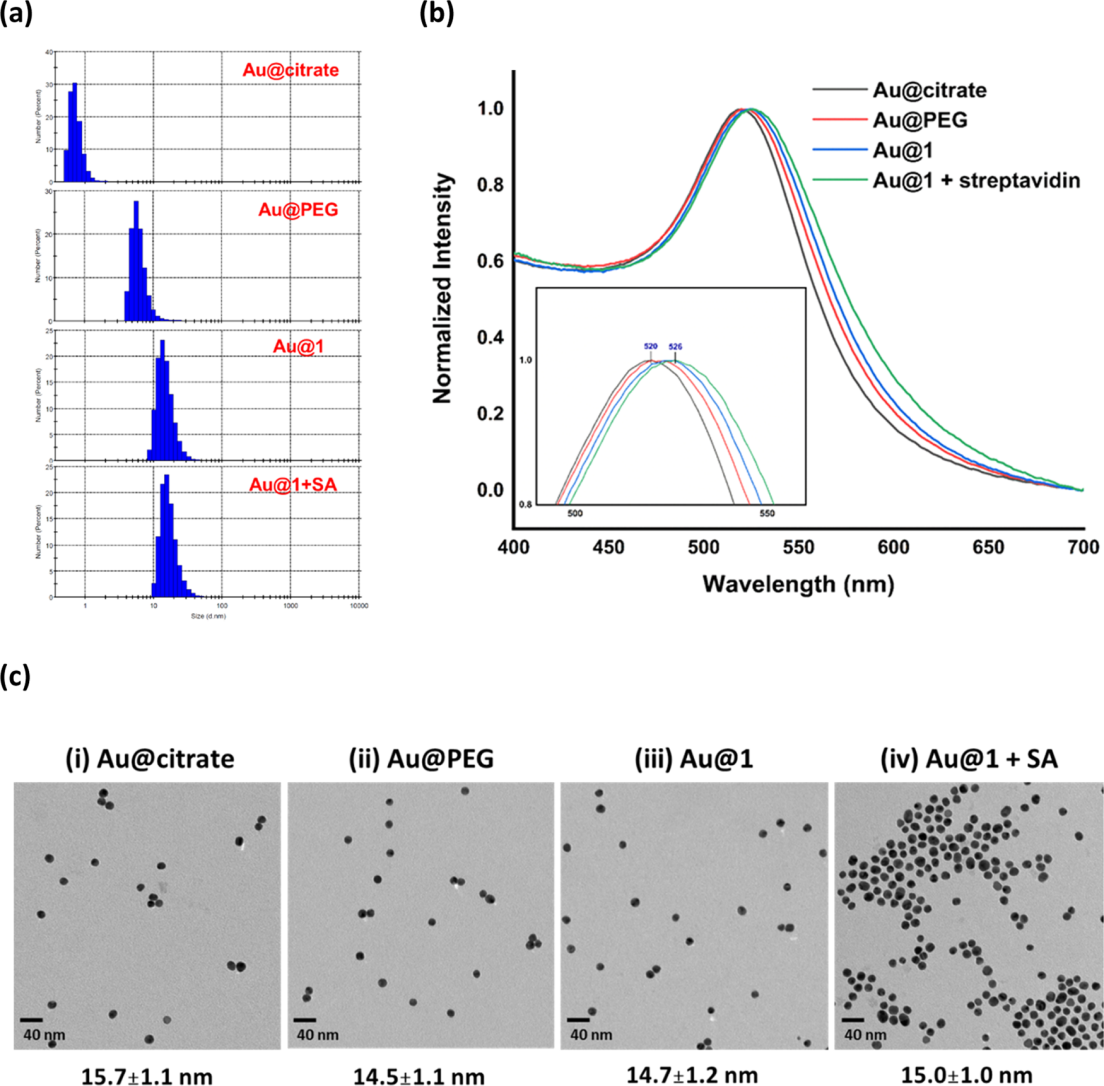
Size distribution of alkynylated-gold nanoparticles. (a) DLS analyses, (b) UV–vis absorption spectra, and (c) TEM images of (i) **Au@citrate**, (ii) **Au@PEG**, (iii) **Au@1**, and (iv) **Au@1** in the presence of streptavidin (SA).

Next, we characterized the size distributions of **Au@citrate**, **Au@PEG**, **Au@1**, and **Au@1** in the presence of streptavidin by using dynamic light scattering (DLS), UV–vis absorption spectroscopy, and transmission electron microscopy (TEM). The DLS results revealed that **Au@citrate** and **Au@PEG** exhibit average hydrodynamic diameters of approximately 28.6 ± 1.7 and 43.5 ± 0.8 nm, respectively (Figure 3a). The alkynylation of **Au@PEG** with **1** (**Au@1**) decreased the size slightly to 31.3 ± 1.0 nm. This is probably due to the displacement of some SH-PEG from the surface, which results in a decrease of the hydrodynamic diameter. As streptavidin is a tetrameric protein that can potentially bind to four biotin molecules, we observed aggregation of the nanoparticles and a dramatic increase in the particle size to 80.9 ± 6.0 nm when **Au@1** was incubated with streptavidin. From the UV–vis spectra, the absorption maxima (λ_max_) of gold nanoparticles showed a gradual bathochromic shift from 520 to 526 nm with increasing functionalization, which is a manifestation of the increasing sizes and changes in the surface properties (Figure 3b).^48^ The TEM images showed that the size of the gold core remained unchanged at an average diameter of about 15 nm (Figure 3c). In agreement with the DLS results, we observed cluster formation of AuNPs in the TEM image of the sample containing a mixture of **Au@1** and streptavidin. These results indicate that the alkynylation of AuNPs does not significantly change the particle size, and they remain dispersive and colloidal in aqueous solutions. The binding of alkynes to AuNPs was also studied using infrared (IR) spectroscopy. A characteristic alkyne stretching band at around 2100 cm^–1^ was observed for **Au@1**, which suggested that the alkyne was incorporated on the surface of the AuNPs (Figure S6). By using HPLC-MS to quantify unreacted **1**, we determined that approximately 61 molecules of **1** were anchored on **Au@1** (Figure S7).

### Comparing Surface Functionalization of AuNPs using Terminal Alkynes, Internal Alkynes, Thiols, and the EDC/NHS Peptide-Bond Methods

**Figure 4:**
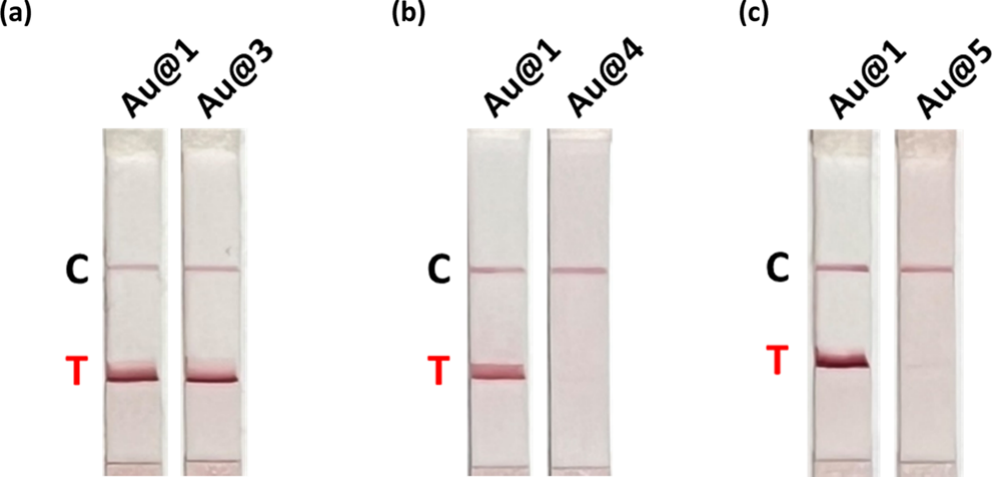
Biotinylation of gold nanoparticles using terminal alkynes, internal alkynes, thiols, and EDC/NHS peptide-coupling methods. LFA test strip images of (a) **Au@1** and **Au@3**, (b) **Au@1** and **Au@4**, (c) **Au@1** and **Au@5**. The peptide-bond reaction between **Au@PEG** and **5** was performed using EDC and NHS reagents in an aqueous solution at 25 °C for 16 h.

The color intensity on the test line is directly proportional to the number of accumulated AuNPs, which, in turn, is related to biotinylation efficiency. To compare the conjugation efficiency of alkynes with the thiol groups, we synthesized compound **3**, which is a thiolated-biotin. **3** was reacted with **Au@PEG** to generate **Au@3**. From the results of the LFA testing, **Au@1** exhibits slightly stronger test line signals than **Au@3** (Figures 4a and S8). To understand the binding mode of the alkyne with AuNPs, compound **4**, which consists of biotin and an internal alkyne, was reacted with **Au@PEG** to obtain **Au@4**. In contrast to **Au@1**, **Au@4** was not able to produce a test line on the LFA membrane (Figures 4b and S9). Compared with the previous spectroscopy studies, our results provide more direct and conclusive evidence to show that AuNPs bind more strongly with the terminal alkyne group to form a stable Au–C bond.^26,49^

We also compared the functionalization efficiency of **Au@1** with that of the AuNPs prepared under classical EDC/NHS peptide-bond coupling conditions. Thus, **Au@5** is the product of the reaction using **Au@PEG**, compound **5**, and EDC/NHS coupling reagents (Figure S10). Despite various attempts to optimize the peptide-bond reaction using different EDC/NHS concentrations and reaction conditions, **Au@5** showed a very faint band on the test line (Figures 4c and S11). These results imply that the activation of the carboxylic groups on the nanoparticles is not as efficient as that in the solution phase. Previously, many studies have reported that the EDC/NHS peptide-coupling method gives a low yield of the carboxylated nanoparticles.^50,51^ We also conducted a dot-blot experiment to validate the results obtained from LFA testing (Figure S12). The results show that **Au@1** and **Au@3** exhibit similar intensities, whereas **Au@2** and **Au@5** give undetectable signals on the membrane. These dot-blot test results are consistent with the LFA results, indicating the reliability of our LFA data.

### Reaction Concentrations, Kinetics, and Stabilities of Alkynylated-Gold Nanoparticles

**Figure 5:**
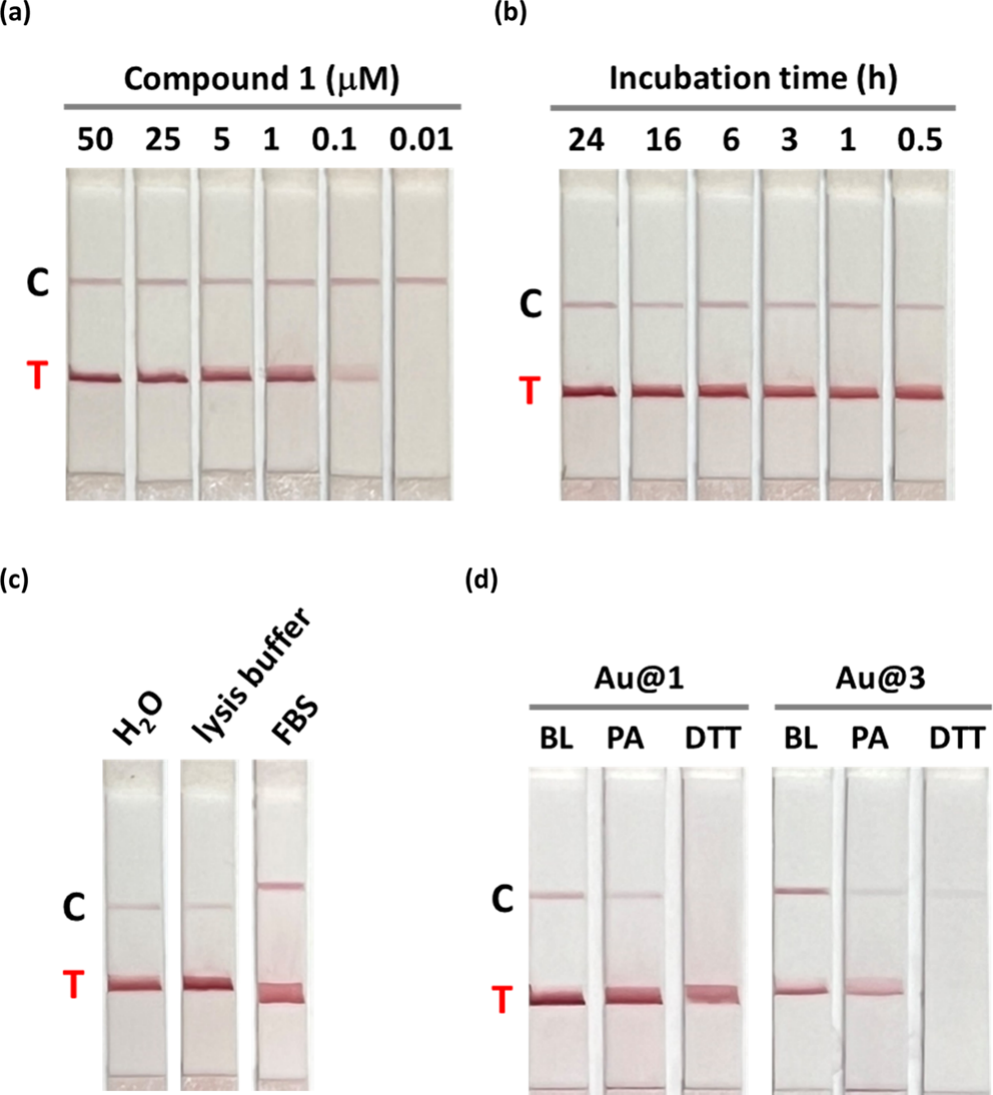
Reaction concentrations, kinetics, and stabilities of alkynylated-gold nanoparticles. (a) The reaction of **Au@PEG** with different concentrations of **1**, (b) reaction time course of **Au@PEG** with 25 μM **1**, (c) formation of functionally active **Au@1** in the presence of 80% lysis buffer and 80% fetal bovine serum (FBS) solution. (d) Stabilities of **Au@1** and **Au@3** after incubation with 10 mM dithiothreitol (DTT) and 4-pentynoic acid (PA) at 25 °C for 1 h. BL: blank.

For AuNP functionalization, it is important that the reaction concentration of alkynes is kept as low as possible to minimize the wastage of functionalized molecules. Thus, various concentrations of compound **1** were reacted with **Au@PEG** to determine the minimum amount of alkynes required for the biotinylation of gold nanoparticles. The LFA results showed that the reaction of **1** with **Au@PEG** is concentration-dependent and an obvious test line can be obtained by using low concentrations of **1** from 0.1 μM (Figures 5a and S13). We also found that **1** reacts very rapidly with **Au@PEG** at room temperature and an obvious test line can be observed after 0.5 h of incubation at 25 °C (Figures 5b and S14).

For biological applications, it is critical that alkynylated-AuNPs remain stable, colloidal, functionally active, and nontoxic in complex biological media and cell culture. Therefore, we investigated the possibility of conducting alkynylation in lysis buffer and fetal bovine serum (FBS). Colloidal and functionally active **Au@1** can be generated even in the presence of 80% lysis buffer and FBS solution (Figures 5c and S15). We further evaluated the binding properties of **Au@1** to streptavidin in FBS, milk, and cell lysates. In these complex running solutions, **Au@1** remains functionally active to bind streptavidin and shows a strong wine red color on the test line (Figure S16). The cellular toxicity of **Au@1** on HeLa, MCF7, A549, and Raw264.7 cell lines was studied using the MTT assay and microscopy images. The results showed that **Au@1** did not exhibit significant toxicity at the test concentrations of 17 and 34 nM (Figure S17). In contrast, a previous study reported the use of 6.2 nM AuNPs to test cellular toxicity.^52^

Compared with the thiolated-AuNPs **Au@3**, we found that **Au@1** is more resistant to decomposition by thiol compounds, such as in the presence of 10 mM dithiothreitol (DTT) (Figures 5d and S18). **Au@3** also shows decreased signals on the test line after being incubated with 10 mM 4-pentynoic acid (PA), suggesting that alkynes can partially displace the thiolated compound from the gold nanoparticles. **Au@1** started to display a weaker signal on the test line after incubation with 100 mM DTT (Figure S19). It is worth mentioning that **Au@Control**, which was prepared by a physical adsorption method using mouse IgG antibody and **Au@citrate**, displayed an attenuating signal with merely 0.01 mM DTT. We also compared the stability of **Au@1** and **Au@3** by incubating the two nanoparticles in 80% FBS, 80% milk, and mouse tumor tissue homogenates at 37 °C for 1 h. The results showed that both **Au@1** and **Au@3** are stable in complex biological media (Figure S20). For the long-term stability of alkynylated-AuNPs, **Au@1** stored at 4 °C for 120 days displayed similar test line signals as the analysis performed on the first day (Figure S21). These results indicate that alkynylated-AuNPs remain in colloidal form to retain their SPR effect in highly complex biological media and covalently formed alkynylated-AuNPs can complement AuNPs functionalized using thiol groups or physical adsorption methods.

### Spectroscopy Studies of Gold Nanoparticles Functionalized with Alkyne-Fluorescein **6**

**Figure 6:**
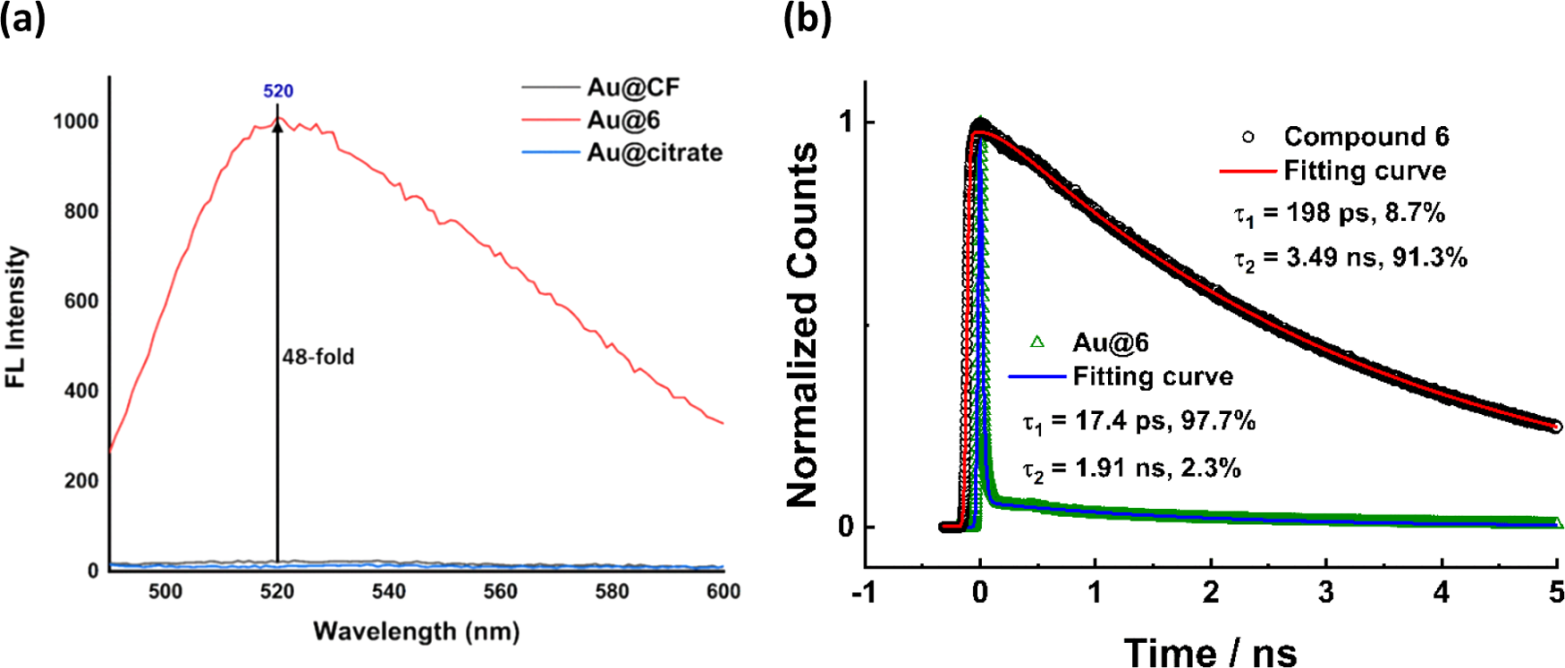
Spectroscopic studies of the gold nanoparticles functionalized with alkyne-fluorescein **6**. (a) Fluorescent spectra of **Au@6**, **Au@CF**, and **Au@citrate**. (b) Fluorescence lifetime spectra of compound **6** and **Au@6**.

To demonstrate that alkynylation can be used as a general approach to functionalize AuNPs, alkyne-fluorescein **6** was incubated with **Au@PEG** to obtain a colloidal **Au@6** solution in red wine color (Figure S22). **Au@6** was characterized by using fluorescence spectroscopy, which showed a characteristic fluorescein emission spectrum with a λ_max_ at 520 nm (Figure 6a). In contrast, the reaction of **Au@PEG** with carboxyfluorescein (**Au@CF**) generated only background fluorescence similar to that of **Au@citrate**. To validate that the alkynylation of AuNPs can be conducted in the presence of different functional groups, compound **6** was reacted with AuNPs capped with poly(ethylene glycol) with different linker lengths carrying terminal NH_2_ or OMe groups. These **6**-functionalized AuNPs exhibit fluorescence that was approximately 11- to 48-fold stronger than that of the background, indicating the robustness of this alkynylation method (Figure S23). By measuring the fluorescence intensity of unreacted **6** in the supernatant, we determined that approximately 109 molecules of **6** were tethered to **Au@6** (Figure S24). In comparison, the number of molecules incorporated on an AuNP through the thiolation method ranges between 15 and 457, as reported in the literature.^14,53−55^

It is well known that fluorescent dyes exhibit reduced fluorescence intensity and lifetime when located near the surface of AuNPs.^56,57^ To show that compound **6** is directly tethered to AuNPs via its alkyne group, we conducted fluorescence lifetime measurements of **6** and **Au@6** using a time-correlated single-photon counting (TCSPC) technique. The fluorescence curve of compound **6** displayed biexponential behavior with rise and decay lifetimes of 198 ps and 3.49 ns, respectively (Figure 6b). This behavior agrees very well with the previous results on fluorescein emission, which indicates that fluorescein has two excited states due to multiple hydroxyl groups.^58^ In contrast, the emission curves of **Au@6** exhibit two very short decay components, a fast decay of ca. 17 ps with an amplitude of 98%, and a slow decay of 1.9 ns. These short time constants reached the temporal resolution of our detection. The biexponential behavior of dye emission due to the plasmonic effect of metal nanoparticles has been reported.^58−60^ Here, the very short fluorescence lifetime of **Au@6** indicated that fluorescein dye most likely lay near the surface of AuNPs about a few nanometers, resulting in large fluorescence quenching. Consistent with the fluorescence lifetime experiments and fluorescence quenching, the addition of 10 mM DTT to **Au@6** released **6** from the gold nanoparticles, which resulted in the recovery of strong fluorescence in the solution (Figure S25).

### Applications of Alkynylated-AuNPs for the Rapid and Sensitive Detection of H_2_O_2_ and Streptavidin on Lateral Flow Assay

**Figure 7:**
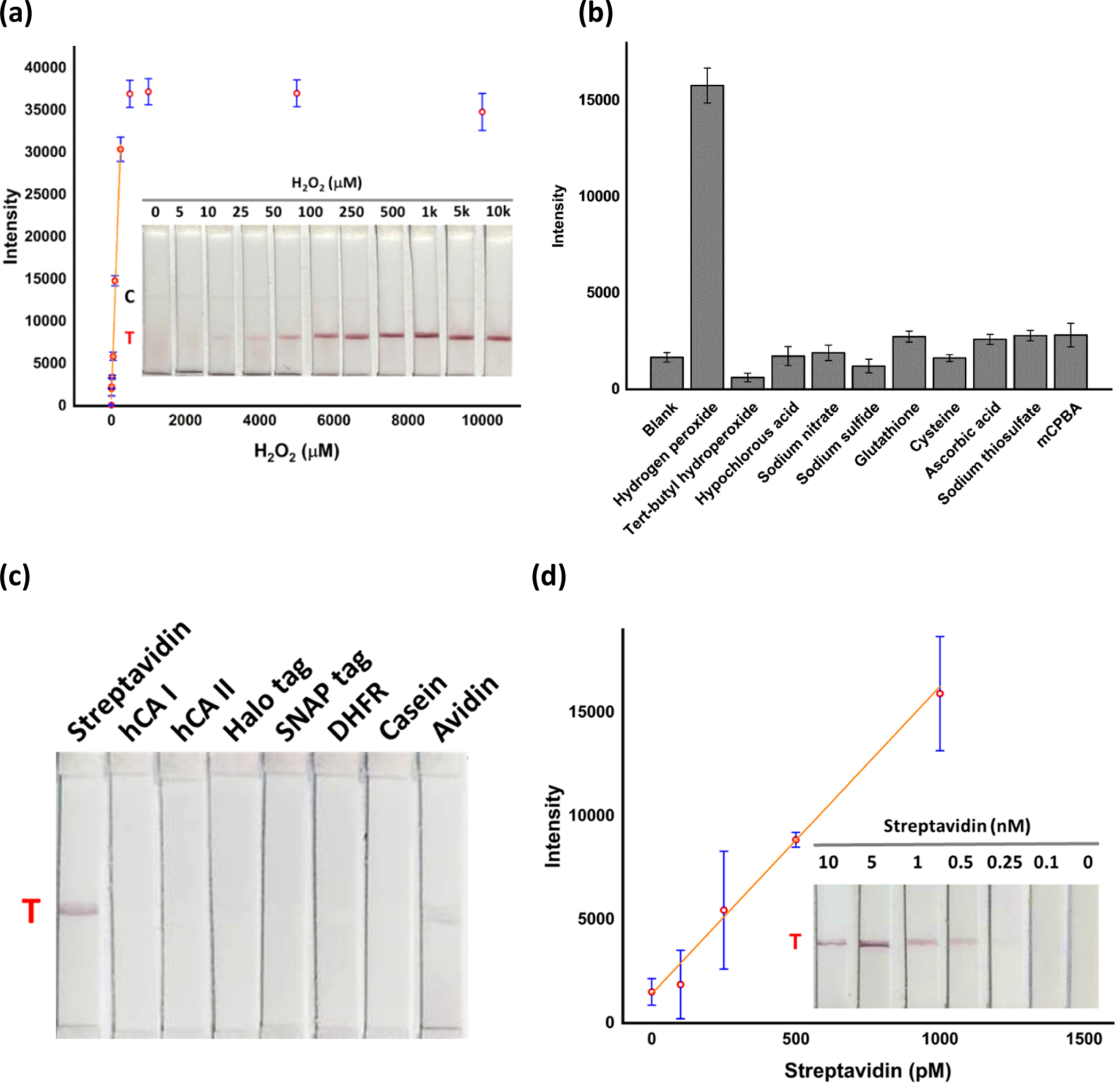
Applications of alkynylated-AuNPs for the rapid and sensitive detection of H_2_O_2_ and streptavidin protein. (a) Quantitative analysis and test strip images after incubation of **Au@7** with various concentrations of H_2_O_2_ for 1 h at 37 °C. (b) Quantitative analysis of the test line signals after incubation of **Au@7** with H_2_O_2_ and other nontarget molecules at 100 μM for 1 h. (c) LFA test strip images of **Au@1** after testing with 25 nM streptavidin and 1 μM of nontarget proteins and (d) quantitative analysis and test strip images after testing different concentrations of streptavidin with **Au@1**. The test line was immobilized with an antistreptavidin antibody.

Previously, we reported various affinity-switchable lateral flow assay (ASLFA) strategies for the rapid analysis of small molecules and reactive species.^61−63^ In these studies, the affinity-switchable biotin probes were conjugated to AuNPs via a carrier protein. However, this conjugation strategy requires longer preparation steps and suffers from a strong background and unsatisfactory detection sensitivity. In this study, we report that an affinity-switchable biotin probe decorated with alkyne groups (probe **7**) can be directly conjugated to **Au@PEG** to form **Au@7**, which was then applied in ASLFA for H_2_O_2_ detection (Figure S26). Various concentrations of H_2_O_2_ were reacted with **Au@7** for 1 h at 37 °C in Tris buffer. Subsequently, the test trips containing avidin test lines were placed in the reaction vial. We observed a H_2_O_2_ concentration-dependent increase of the test line signals, and the concentration of H_2_O_2_ as low as 10 μM can be easily detected visually (Figure 7a). The theoretical limit of detection (LOD) was calculated to be about 0.66 μM H_2_O_2_ with a linear range of 0–250 μM. For comparison, the previous ASLFA strategy for H_2_O_2_ testing gave visual and theoretical LOD values of 50 μM and 25 μM, respectively.^62^ Furthermore, we also showed that H_2_O_2_ detection using **Au@7** has better detection sensitivity than the fluorescent turn-on chemical probe, which can only respond to H_2_O_2_ at a concentration of about 1 mM (Figure S27). Next, we investigated target selectivity by incubating **Au@7** with different oxidants and reducing agents. The results indicated that **Au@7** reacts specifically with H_2_O_2_ (Figures 7b and S28). To demonstrate that alkynylated-AuNPs can be applied for chemical sensing in complex samples, H_2_O_2_ was detected in 50% FBS using **Au@7**. The results showed that H_2_O_2_ can be detected and quantified in 50% FBS with a visual detection limit of approximately 5 μM and a linear range of 0–250 μM (Figure S29). These results were similar to those of H_2_O_2_ detection performed under clean buffer conditions.

Many lateral flow assays use a sandwich-type strategy for protein detection, as this approach offers high target specificity and analytical sensitivity.^64,65^ Typically, antibodies that can bind to two different epitopes of the target protein are needed to form sandwich complexes. However, it is often difficult to identify such antibodies. Herein, we show that the formation of a sandwich complex for protein detection can be achieved using only one antibody and a small protein ligand. To demonstrate this approach, **Au@1** and an antistreptavidin antibody immobilized on the test line were used for the detection of streptavidin using sandwich-type LFA (Figure S30). In the presence of 25 nM streptavidin, an obvious test line was observed on the LFA membrane (Figures 7c and S31). In contrast, a test line was not observed when 1 μM of nontarget proteins was added to the **Au@1** solution. Next, we determined detection sensitivity by titrating different concentrations of streptavidin with **Au@1**. The results showed that this sandwich approach could provide visual and theoretical LOD values as low as 250 pm and 33 pM, respectively (Figure 7d). In comparison, previous methods for streptavidin detection require complicated procedures and are less sensitive (e.g., LOD ≈ 66 pM).^66−68^ Since streptavidin is often employed for biomolecular isolation, enrichment, and signal amplification, we believe that our approach can be a very useful tool for more extensive research in biochemistry, analytical chemistry, and proteomics.

## Conclusions

By using a lateral flow assay and spectroscopy techniques, we show that small molecules derivatized with an alkyne group can be used for the rapid and simple surface functionalization of gold nanoparticles. Our results showed that the reaction of alkynes with AuNPs is fast (<30 min), can be conducted in complex media, and requires only a small amount of alkynes (≈0.1 μM) to achieve sufficient conjugation. Colloidal alkynylated-AuNPs are stable for long-term storage and are resistant to decomposition in complex biological media. We also demonstrated the application of alkyne-functionalized AuNPs for the detection of hydrogen peroxide (H_2_O_2_) and streptavidin using affinity-switchable and sandwich-type lateral flow assay approaches.

Currently, most small-molecule functionalized AuNPs are obtained using derivatives of thiols and amines with EDC/NHS peptide-coupling reagents. Compared with these two classical methods, the alkynylation of AuNPs gives higher conjugation efficiency, as shown by the stronger signals in the LFA test paper. Compared with thiol groups, which typically require complicated synthetic procedures to prepare and are vulnerable to oxidation, the alkyne group is relatively inert and can be obtained rapidly and easily using standard organic chemistry reactions (e.g., peptide reaction and alkylation). Therefore, diverse functional molecules can be incorporated into the surface of the AuNPs. Furthermore, colloidal alkynylated-AuNPs can even be generated in complex biological media (e.g., 80% FBS and lysis buffer), demonstrating potential applications in the analyses of alkyne-labeled proteins and DNAs. We believe that the surface functionalization of AuNPs using biorthogonal alkyne derivatives can be an important strategy in developing novel nanomaterials for applications in diagnosis, therapeutics, and material sciences.

## Experimental Section

### Preparation of Citrate-Capped Gold Nanoparticles (**Au@citrate**)

Gold nanoparticles were prepared by using a sodium citrate reduction method (Turkevich method). A stirred solution of chloroauric acid (1 mM) in 50 mL H_2_O was heated until it was boiled vigorously. Trisodium citrate solution (38.8 mM) was added to the reaction mixture, which was then heated for 10 min. The AuNP solution was then cooled to room temperature and stored at 4 °C until further use. The diameter of the AuNP seed was measured to be approximately 15 nm based on the maximum absorption peak.

### Preparation of Alkynylated Gold Nanoparticles (**Au@alkyne Derivatives**)

200 μL of **Au@citrate** (Abs_max_ = 520 nm) solution was transferred to a microcentrifuge tube. The solution was centrifuged at 5000*g* for 30 min. The supernatant was removed and the AuNPs were modified with SH-PEG(1K)-CO_2_H in H_2_O at room temperature for 1 h to obtain **Au@PEG**. The reaction mixture was centrifuged at 10,000 rpm for 30 min, and the supernatant was removed. The pellet was reacted with the alkyne derivatives overnight at 25 °C with stirring at 120 rpm. The reaction mixture was centrifuged at 10,000 rpm for 30 min and the supernatant was removed. 1 mL of 25 mM Tris buffer (pH = 7.4) was added to the pellet and incubated for 10 min at 25 °C with stirring at 120 rpm. The solution was then centrifuged at 10,000 rpm for 30 min. The supernatant was removed and the **Au@alkyne** derivative pellets were stored at 4 °C until further use.

### Preparation of Mouse IgG-Conjugated Gold Nanoparticles (**Au@control**)

1 mL (Abs_max_ = 520 nm) **Au@citrate** solution was transferred to a microcentrifuge tube, and K_2_CO_3_ was added to the microcentrifuge tube to adjust the pH value of the AuNP solution to 9. AuNPs were functionalized with mouse IgG by physical adsorption at 25 °C for 20 min with stirring at 200 rpm. The solution was centrifuged at 12,000 rpm for 15 min. The supernatant was then removed. Then, 300 μL of conjugate buffer solution (5 mM sodium borate, 0.1% bovine serum albumin (BSA), 0.15% Tween 20, and 5% d-sucrose) was added to the pellet and **Au@control** was stored at 4 °C until further use.

### Preparation of Lateral Flow Assay Test Strips

The control and test lines were coated onto the membranes using a rapid test printer (Regabio Inc., Taiwan). Alkaline phosphatase-conjugated streptavidin (0.5 mg/mL) and goat antimouse IgG (0.25 mg/mL) were used to coat the test line and control line, respectively. After coating, the membranes were incubated at 37 °C for 30 min to remove any excess moisture. The length and width of the test strip were 5.9 and 0.35 cm, respectively.

### Testing of Reaction Concentrations and Kinetics of Alkynylated Biotin with **Au@PEG**

To study the effect of the concentration of probe **1** on the formation of **Au@1**, the prepared **Au@PEG** pellets were reacted with 50, 25, 5, 1, 0.1, and 0.01 μM of compound **1** in water at 25 °C with stirring at 120 rpm overnight. To study the reaction kinetics of alkynylated biotin with AuNPs, **Au@PEG** pellets were reacted with 25 μM of compound **1** in water for 24, 16, 6, 3, 1, and 0.5 h, at 25 °C with stirring at 120 rpm. The reaction mixture was centrifuged at 10,000 rpm for 30 min and the supernatant was removed. 1 mL of 25 mM Tris buffer (pH = 7.4) was added to the pellet and incubated for 10 min at 25 °C with stirring at 120 rpm. The solution was centrifuged at 10,000 rpm for 30 min and the supernatant was removed. The **Au@1** pellet, **Au@control**, and 6.25 μL of BSA (2 mM) were mixed in Tris buffer (50 mM) sequentially. Test strips (SA-ALP coated test line) were then immersed in the mixture for 15 min (total volume: 50 μL). The LFA test strip images were captured using a handphone camera. The test lines were recorded using the ChemiDoc Touch Imaging System (Biorad Inc., CA), and the intensities were analyzed using Image lab software (Biorad Inc., CA).

### Stability Tests of **Au@1** and **Au@3** in Biological Media

2 μL of **Au@1/Au@3**, 2 μL of **Au@control**, and 6.25 μL of BSA (2 mM) were sequentially mixed in Tris buffer (50 mM), FBS solution (80%), cow milk (80%), and cell lysate (80%), respectively. The mixed nanoparticles were incubated in the biological media at 37 °C for 1 h. For testing with DTT, **Au@1** and **Au@3** were incubated with 10 mM DTT in Tris buffer (50 mM), respectively, at 37 °C for 1 h. Subsequently, the **Au@control** and 6.25 μL of BSA (2 mM) were added to the mixture. For the analysis of the testing results, test strips (SA-ALP-coated test line) were immersed in the mixture for 15 min (total volume: 50 μL). The LFA test strip images were captured using a handphone camera. The test lines were recorded using a ChemiDoc Touch Imaging System (Biorad Inc., CA), and the intensities were analyzed using Image lab software (Biorad Inc., CA).

### Detection of H_2_O_2_ with Lateral Flow Assay

For the selectivity test, **Au@7** was incubated with different chemicals (100 μM) in Tris buffer (50 mM) at 37 °C for 1 h. Subsequently, **Au@control** and 6.25 μL of BSA (2 mM) were added to the mixture. The test strips (avidin-coated test line) were immersed in the reaction mixture for 15 min (total volume: 50 μL). For the sensitivity test, **Au@7** was incubated in Tris buffer (50 mM) with different concentrations of H_2_O_2_ (0, 5, 10, 25, 50, 100, 250, 500, 1000, 5000, and 10,000 μM) at 37 °C for 1 h. Subsequently, the control particles and 6.25 μL of BSA (2 mM) were added to the mixture. The test strips were immersed in the reaction mixture for 15 min (total volume: 50 μL). The LFA test strip images were captured by using a handphone camera. The test lines were recorded using a ChemiDoc Touch Imaging System (Biorad Inc., CA), and the intensities were analyzed using Image lab software (Biorad Inc., CA).

### Detection of Streptavidin with Lateral Flow Assay

Streptavidin (SA) was stored in a PBS buffer solution at −4 °C. Various concentrations of SA were added to **Au@1** in Tris buffer (total volume: 50 μL). Subsequently, antistreptavidin-immobilized test strips were immersed in the reaction mixture to initiate detection. The LFA test strip images were captured using a handphone camera. The test lines were recorded using a ChemiDoc Touch Imaging System (Biorad Inc., CA), and the intensities were analyzed using Image lab software (Biorad Inc., CA).

## Supporting Information Available

Probe synthesis, LFA images, fluorescence spectra, quantitative analysis, and NMR and HRMS spectra (PDF)
