# Supplementary material for: Surface Functionalization of Gold Nanoparticles Using Alkyne Derivatives: Applications in Chemical Sensing
Source: ACS Appl Mater Interfaces. 2024 Oct 19;16(43):58262–73. doi: 10.1021/acsami.4c12063 (PMC11533169; doi:10.1021/acsami.4c12063)
Supplement: Supplementary file 1 — am4c12063_si_001.pdf [file am4c12063_si_001.pdf]

Supporting Information

Surface Functionalization of Gold Nanoparticles using Alkyne Derivatives: Applications in Chemical Sensing

Yun-Qiao Liu,[†,#] Yi-Cheng Chao,[†,#] Shun-Qiang Xu,[†,#] Yun-Rong Peng,[†] Jhih-Jie Syu,[†] Xiang-He Yang,[†] Yung-Kun Pan,[†] Po-Cheng Lin,[†] Ling-Ling Weng,[†] I-Chia Chen[†] and Kui-Thong Tan[†,‡,*]

[†] Department of Chemistry, National Tsing Hua University, 101 Section 2, Kuang-Fu Road, Hsinchu 300044, Taiwan

[‡] Department of Medicinal and Applied Chemistry, Kaohsiung Medical University, Kaohsiung 80708, Taiwan

[#] Y.-Q. L., Y.-C.C., and S.-Q.X. contributed equally to this work

Corresponding Author: kttan@mx.nthu.edu.tw

Materials and instruments

Chemicals and reagents were purchased from Sigma-Aldrich and TCI and used without further purification. All solvents (dimethyl sulfoxide (DMSO), N,N-dimethylformamide (DMF), acetonitrile, dichloromethane (CH_2Cl_2), hexane, ethyl acetate, chloroform ($CHCl_3$), and methanol) were purchased from Sigma-Aldrich and TCI and used without further treatment or distillation. Alkaline phosphatase-conjugated Streptavidin (SA-ALP), mouse IgG and goat anti-mouse IgG were purchased from Jackson ImmunoResearch (USA). Avidin was purchased from Prospec. Streptavidin was purchased from Fitzgerald. Anti-streptavidin antibody was purchased from Rockland. The polyethylene glycol derivatives were purchased from Sigma-Aldrich.

Thin layer chromatography (TLC) was performed on TLC-aluminum sheets (Silica gel 60 F254, Merck). Flash column chromatography was performed with silica gel (230-400 mesh, Merck). HPLC analysis was performed with an analytical column (XBridge BEH C18 Column, 130Å, 5 mm, 4.6 mm x 250 mm). The lateral flow assay membrane (Whatman FF120HP membranes) was purchased from Cytiva. The backing card was purchased from Prisma Biotech. The absorbent pad was purchased from Merck. The sample pad was purchased from Advanced Microdevices. ^{1}H, ^{13}C, and ^{19}F nuclear magnetic resonance (NMR) spectra were recorded on Bruker-400 and Bruker-600 with ^{1}H chemical shifts (δ) reported in ppm relative to the solvent residual signals of *d*-chloroform (7.24 ppm), d-methanol (3.30 ppm), *d*-DMSO (2.49 ppm), and D_2O (4.79 ppm). ^{13}C chemical shifts (δ) were reported in ppm relative to the solvent residual signals of *d*-chloroform (77.0 ppm), *d*-methanol (49.0 ppm), and *d*-DMSO (39.5 ppm). Coupling constants were reported in Hz. High-resolution mass spectra (HRMS) with electrospray ionization (ESI) were measured on JOEL JMS-T100LP 4G. The UV-Vis absorption and fluorescent spectra were recorded on TECAN Infinite M200 PRO and Jasco V570, respectively. The TEM images were obtained using Hitachi HT7700. The DLS analyses were performed on Malvern Zetasizer Nano. All the LFA images presented in the manuscript were captured using a handphone camera. The test line intensities were recorded using ChemiDoc Touch Imaging system (Bio-rad Inc, CA, USA) and analyzed using Image lab software (Bio-rad Inc, CA, USA).

(a)

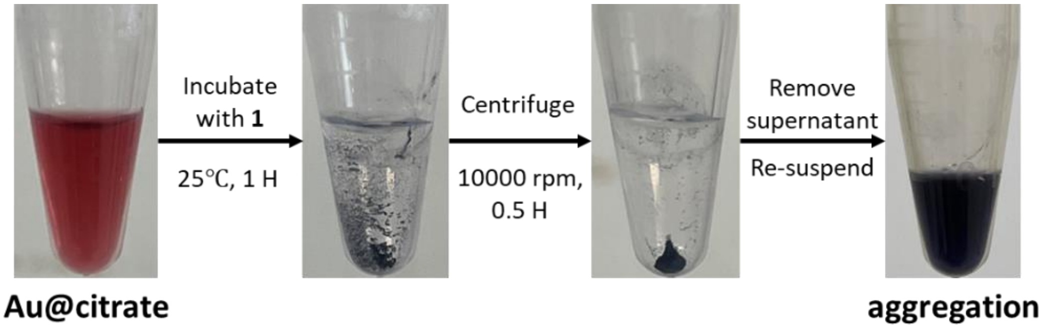

(b)

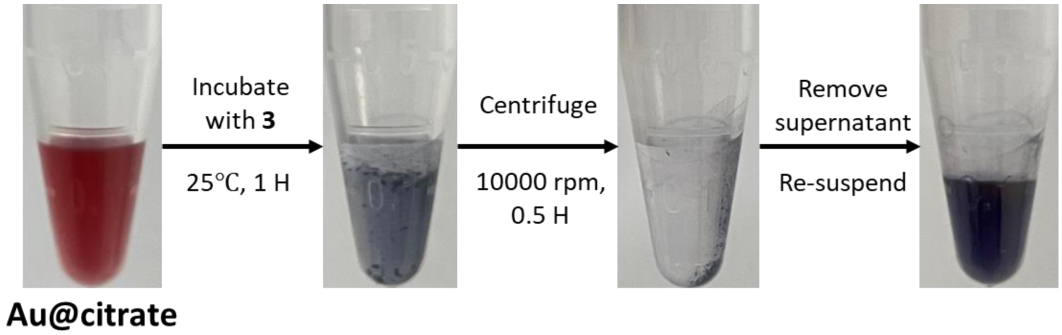

Figure S1. Reaction of compound (a) **1** or (b) **3** with the citrate-capped gold nanoparticles **Au@citrate** results in the formation of aggregated blue color alkynylated-gold nanoparticles.

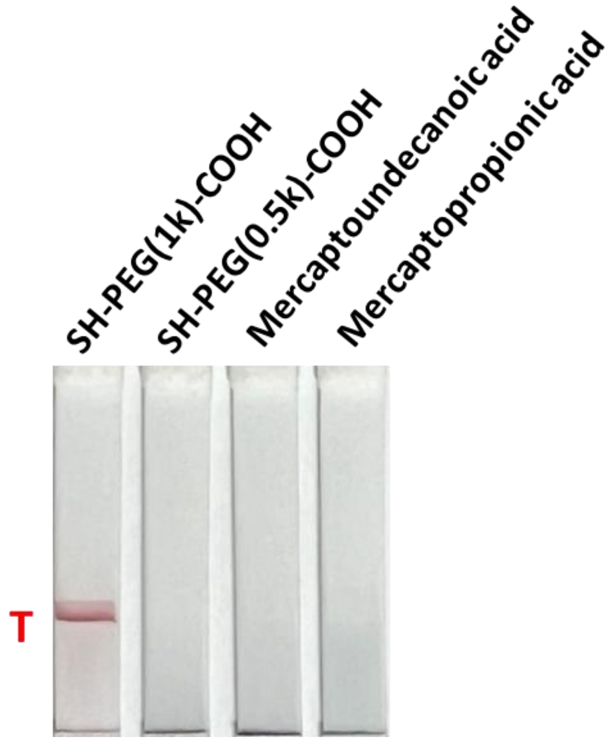

Figure S2. LFA testing of AuNPs functionalized with **1** and capped with different linkers.

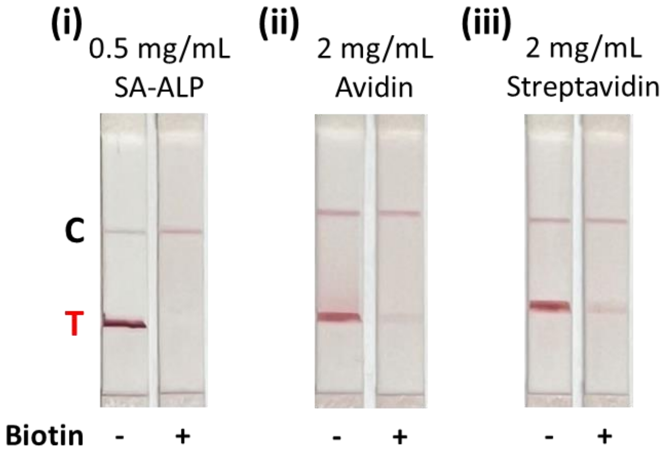

Figure S3. Testing of **Au@1** using the LFA test line immobilized with (i) 0.5 mg/mL streptavidin-alkaline phosphatase, SA-ALP (ii) 2 mg/mL avidin, and (iii) 2 mg/mL streptavidin. The results showed that SA-ALP is the best protein variant for capturing **Au@1** due to the stronger test line signal while requiring a lower concentration.

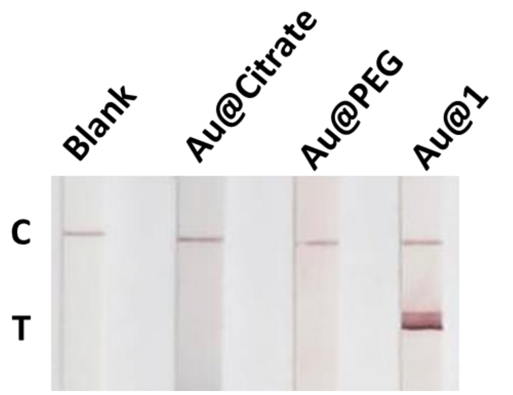

Figure S4. Lateral flow assay test strips treated with **Blank** (without test particles), **Au@citrate**, **Au@PEG** and **Au@1**.

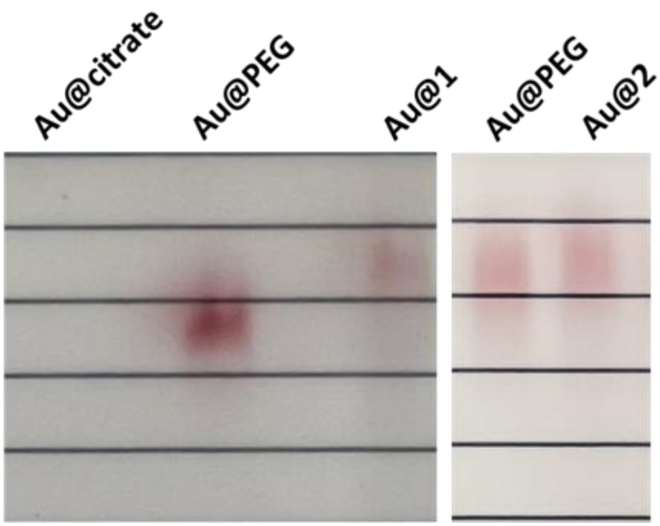

Figure S5. Electrophoretic migration rate of **Au@citrate**, **Au@PEG**, **Au@1** and **Au@2** on agarose gel.

(a)

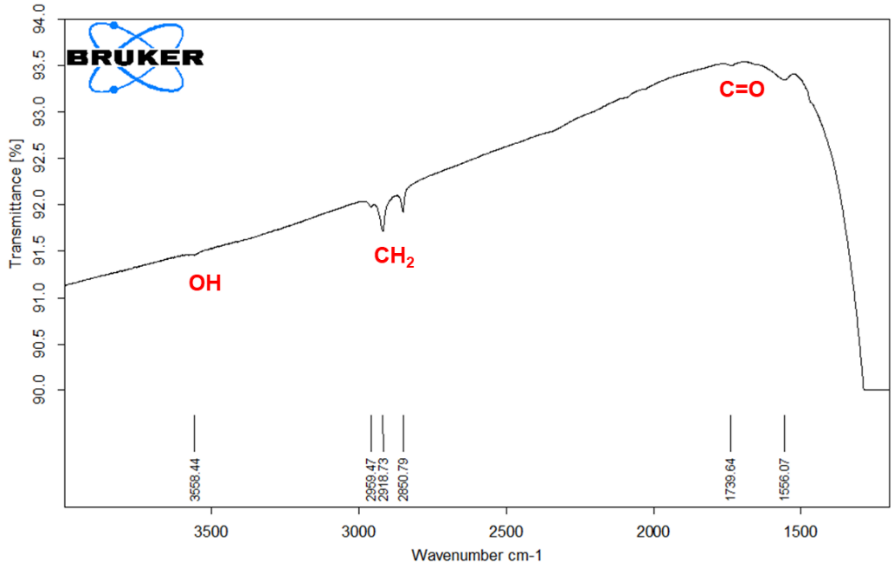

(b)

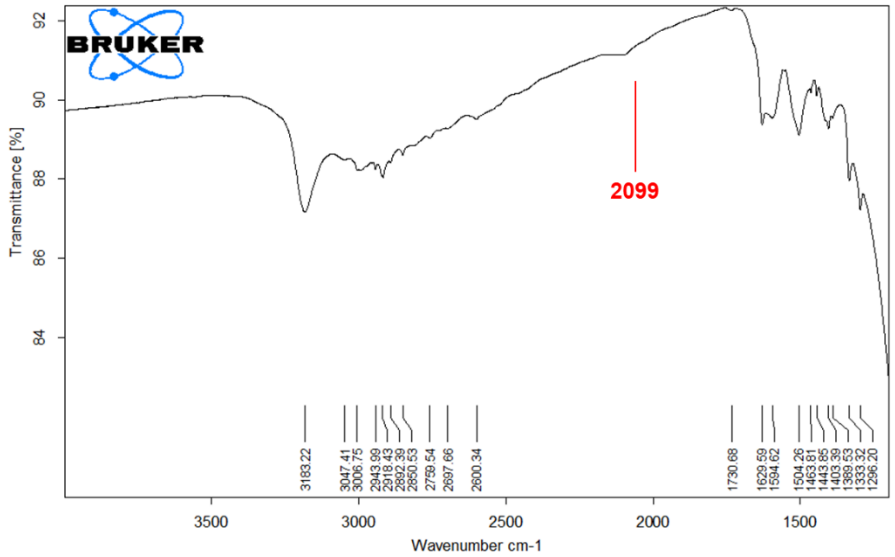

Figure S6. Infrared spectra of (a) **Au@PEG** and (b) **Au@1**.

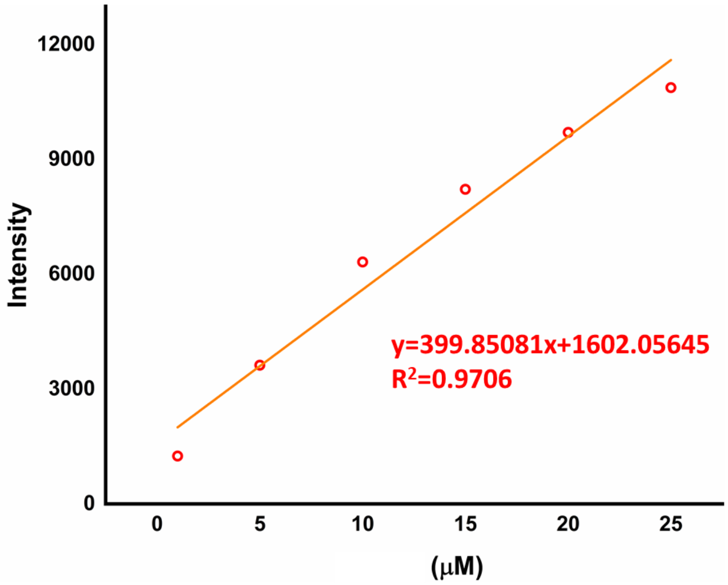

Figure S7. Calibration curve of compound **1** generated by using HPLC-MS. The intensity of unreacted 1 (initial concentration 25 μM) was fitted to the equation to obtain the density of **1** on AuNPs.

(a) (b)

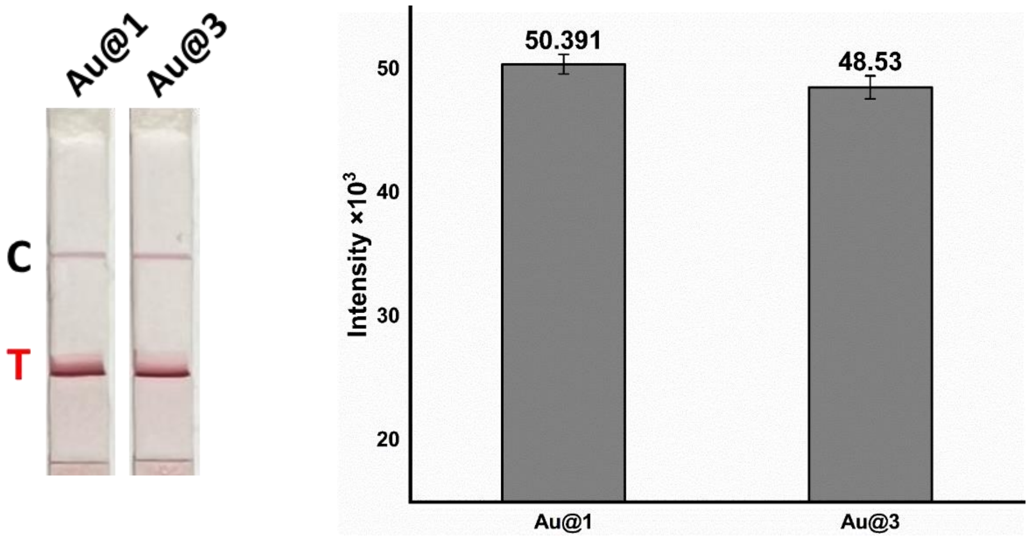

Figure S8. (a) Lateral flow assay images (b) quantitative analysis of the LFA test line signals generated by using **Au@1** and **Au@3**.

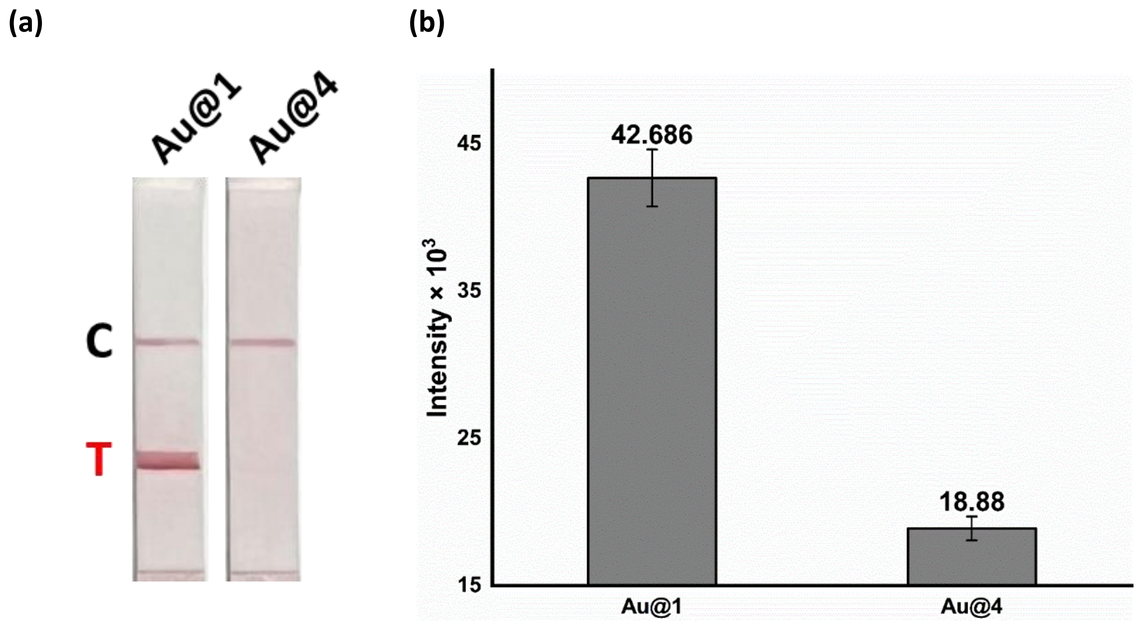

Figure S9. (a) LFA images and (b) quantitative analysis of the **Au@1** and **Au@4** LFA test line signals.

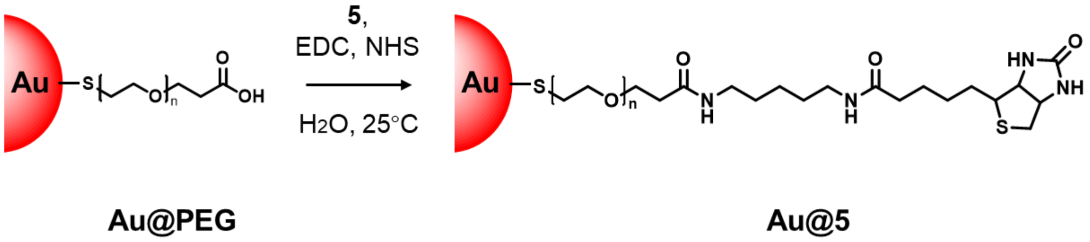

Figure S10. Schematic illustration of **Au@5** preparation using **Au@PEG** and compound **5** in the presence of EDC/NHS peptide coupling reagents at 25 °C for 16 hours.

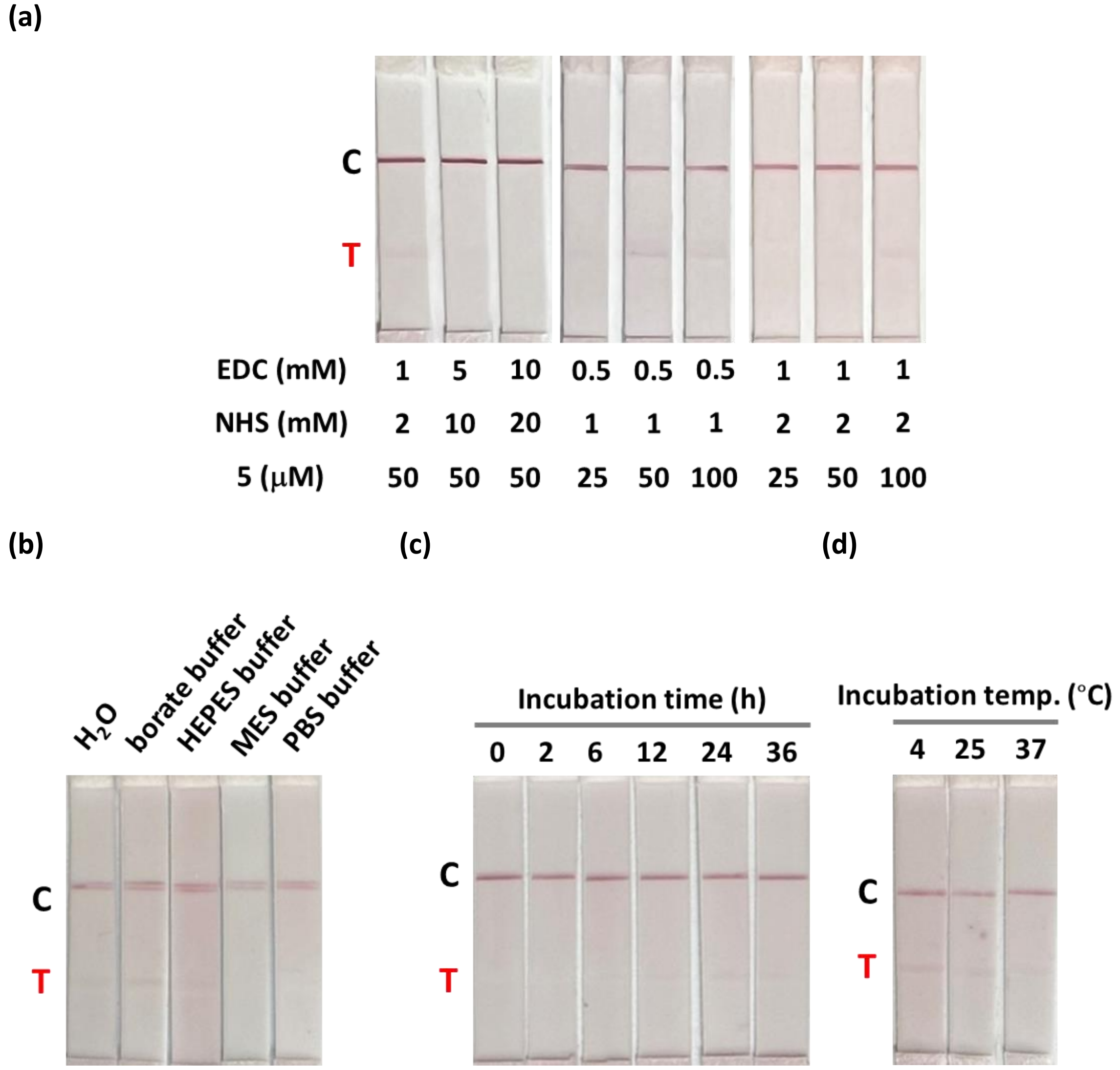

Figure S11. Various EDC/NHS reaction conditions to optimize the peptide bond formation between **Au@PEG** and compound **5**. (a) Reaction of **Au@PEG** with different concentrations of EDC, NHS and **5**. The peptide coupling reaction was performed in an aqueous solution at 25 °C for 16 hours. Reaction of **Au@PEG** and **5** (100 μM) with EDC (1 mM) and NHS (1 mM) at 25 °C for 16 hours (b) in different buffers, (c) different incubation times and (d) different temperatures.

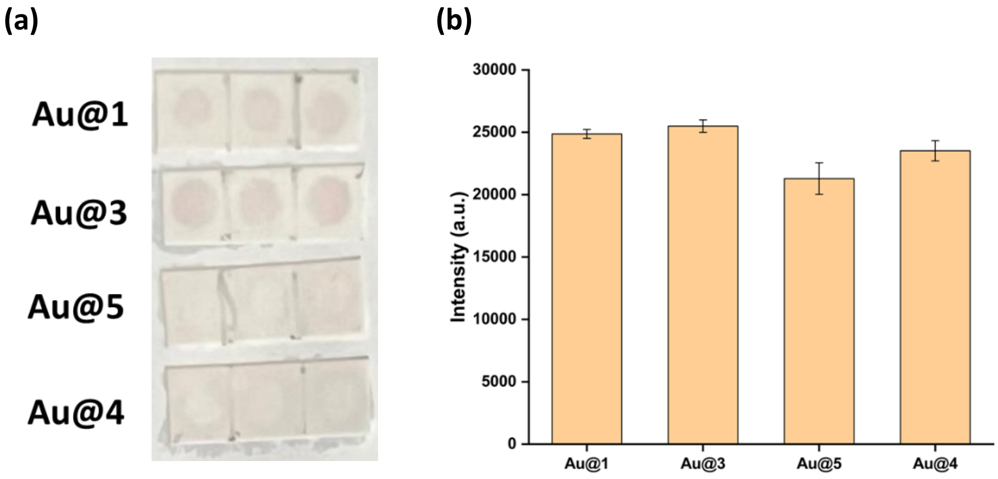

Figure S12. (a) Dot blot images (b) quantitative analysis of the dot blot signals generated by using **Au@1**, **Au@3**, **Au@5** and **Au@4**. The nitrocellulose membranes were spotted with 0.5 mg/mL streptavidin. The gold nanoparticles were incubated with the dot blot membranes at room temperature for 12 hours.

(a)

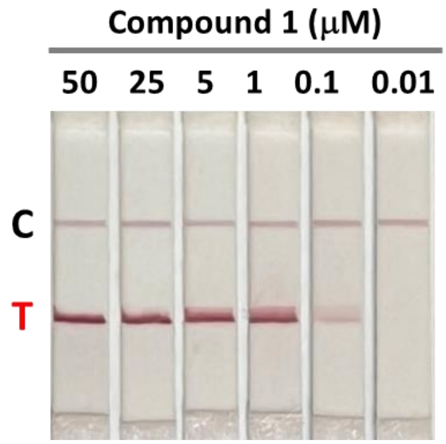

(b)

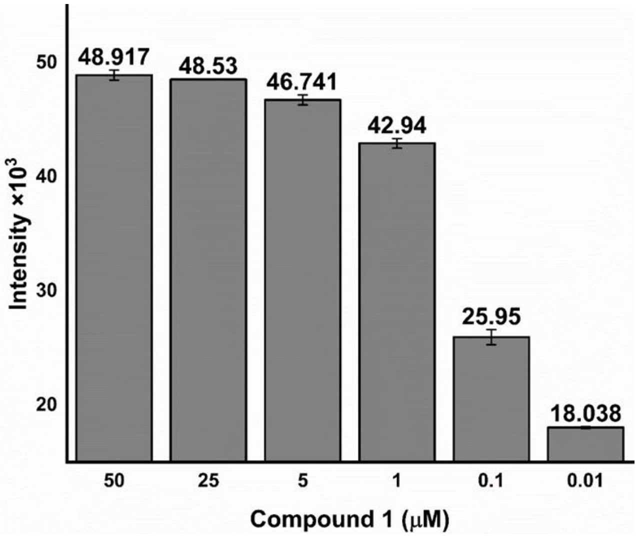

Figure S13. (a) LFA images and (b) quantification of the **Au@1** test line signals in which the gold nanoparticles are generated by treatment of **Au@PEG** with different concentrations of compound **1**.

(a) (b)

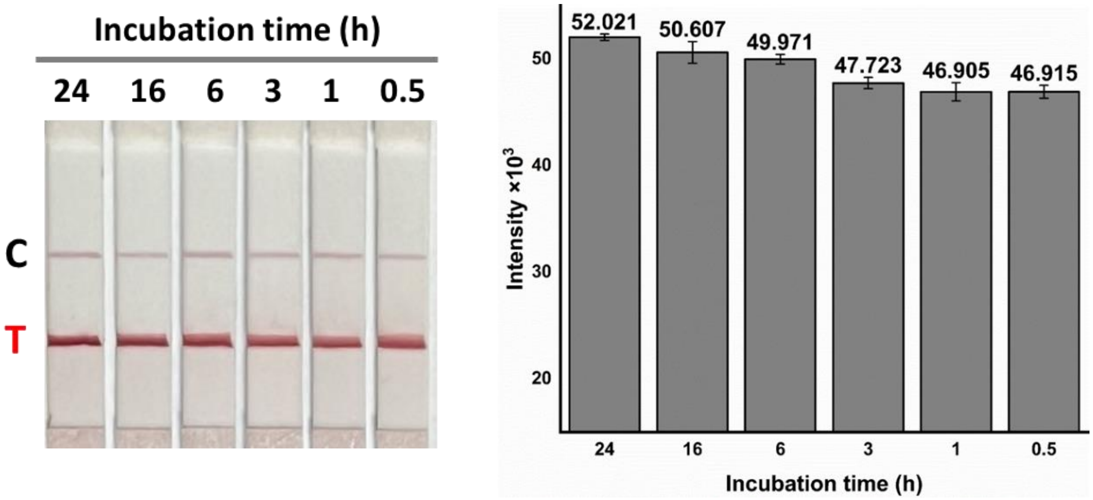

Figure S14. Reaction time course of Au@PEG and compound 1. (a) LFA images and (b) quantification of the **Au@1** test line signals in which **Au@PEG** and 25 μM **1** were incubated for different time intervals.

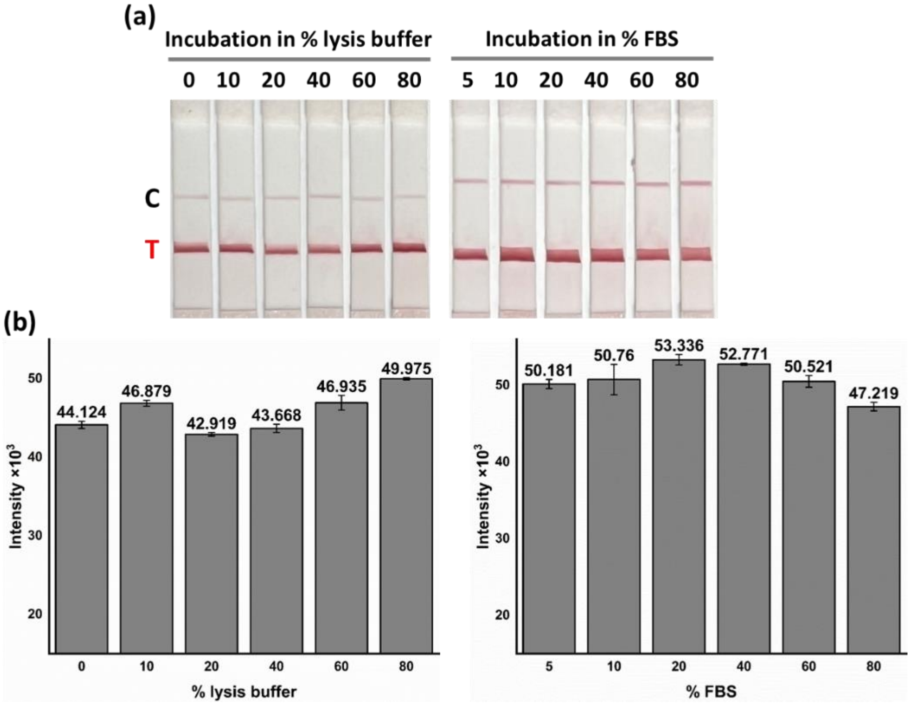

Figure S15. (a) LFA images and (b) quantification of the **Au@1** test line signals in which alkynylation of **Au@PEG** with 25 μM **1** was performed in various concentrations of lysis buffer and FBS. (Lysis buffer: 25 mM Tris-HCl pH 7.4, 150 mM NaCl, 1 mM EDTA, 1% NP-40 and 5% glycerol)

(a) (b)

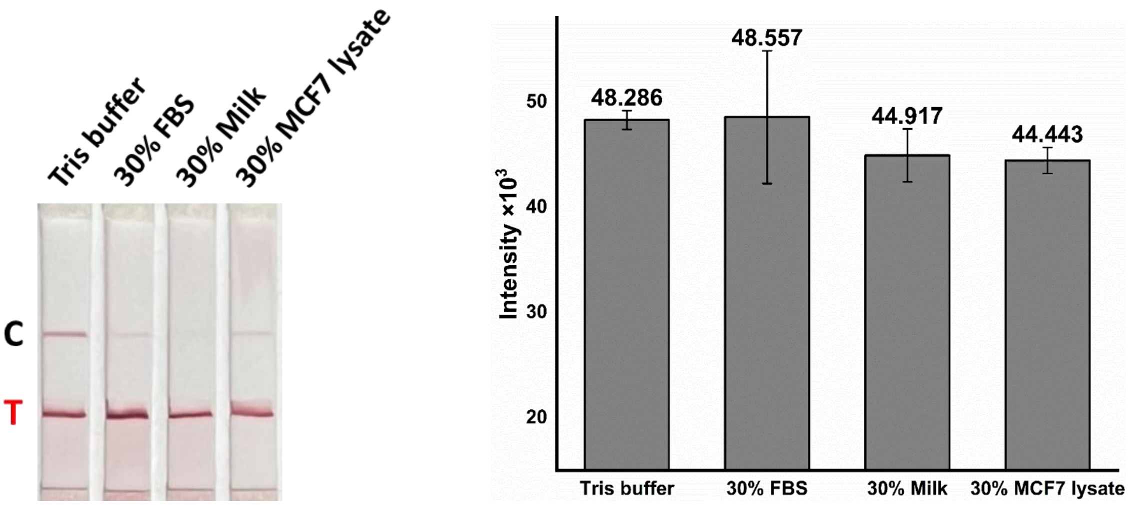

Figure S16. (a) LFA images and (b) quantitative analysis of the **Au@1** test line signals for the testing performed in Tris buffer, 30 % FBS, 30 % milk and 30 % MCF7 cell lysates.

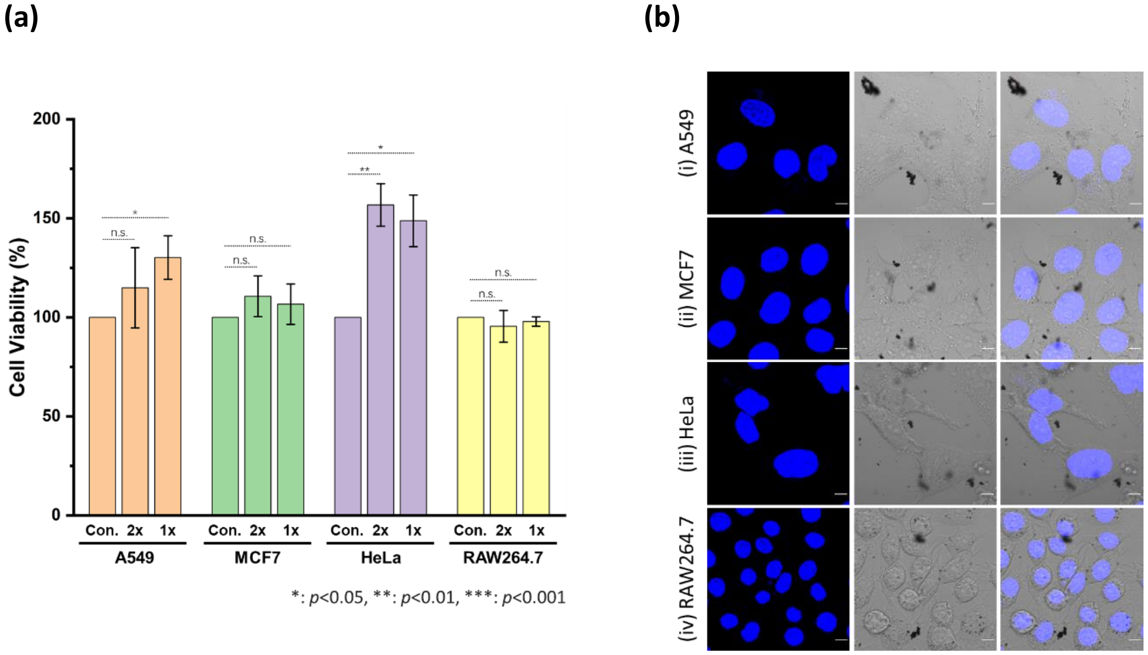

Figure S17. Cellular toxicity test of **Au@1**. (a) Determination of **Au@1** toxicity on HeLa, MCF7, A549 and Raw264.7 cell lines using MTT assay. The cells were incubated with 17 nM (1x) and 34 nM (2x) of **Au@1** at 37 °C for 4 hours, respectively. (b) Fluorescent and bright-field images of HeLa, MCF7, A549 and Raw264.7 cell lines after treatment with 34 nM (2x) of **Au@1** at 37 °C for 4 hours. In the case of HeLa and A549 cells, the cell viability percentages exceed the control group. This is probably due to absorption of **Au@1** into the cells which caused higher absorption intensity obtained during MTT measurements.

(a)

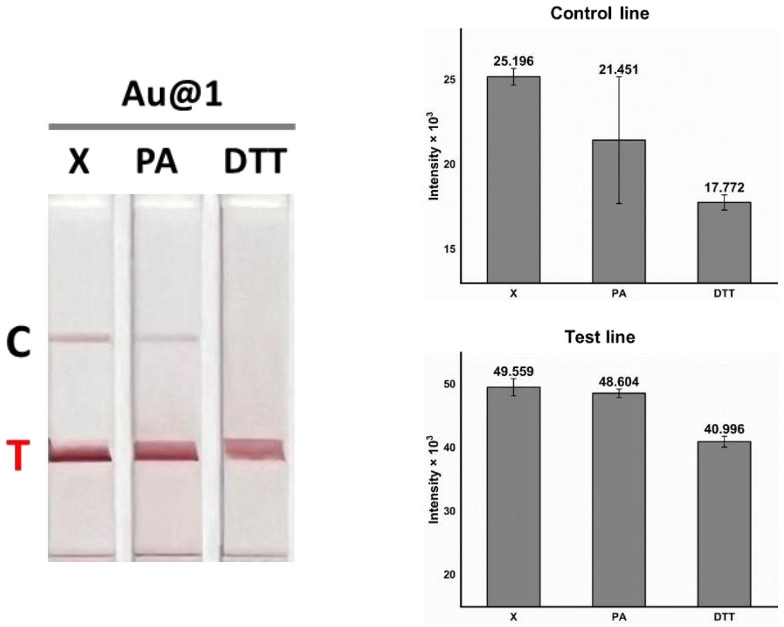

(b)

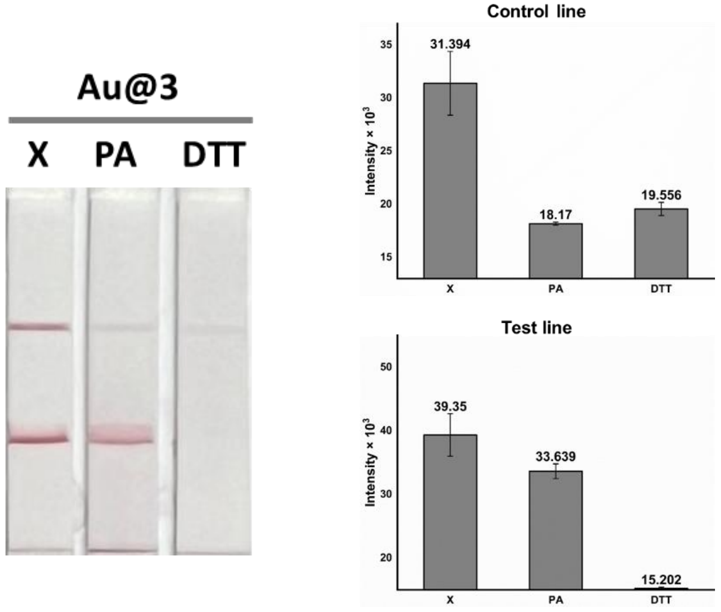

Figure S18. Quantitative analysis of the control and test line signals of (a) **Au@1** and (b) **Au@3** after incubating the gold nanoparticles with 10 mM dithiothreitol (DTT) and 4-pentynoic acid (PA) for 1 hour at 25 °C. X = blank.

(a)

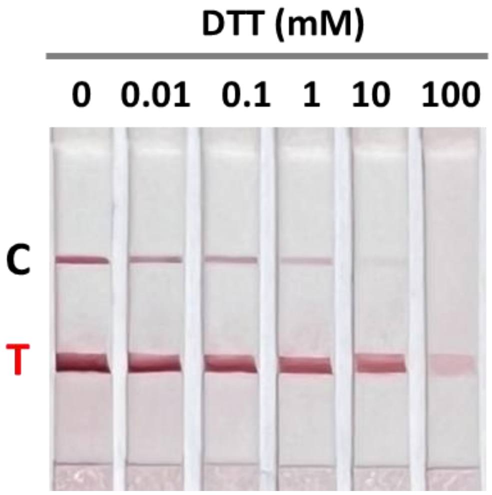

(b)

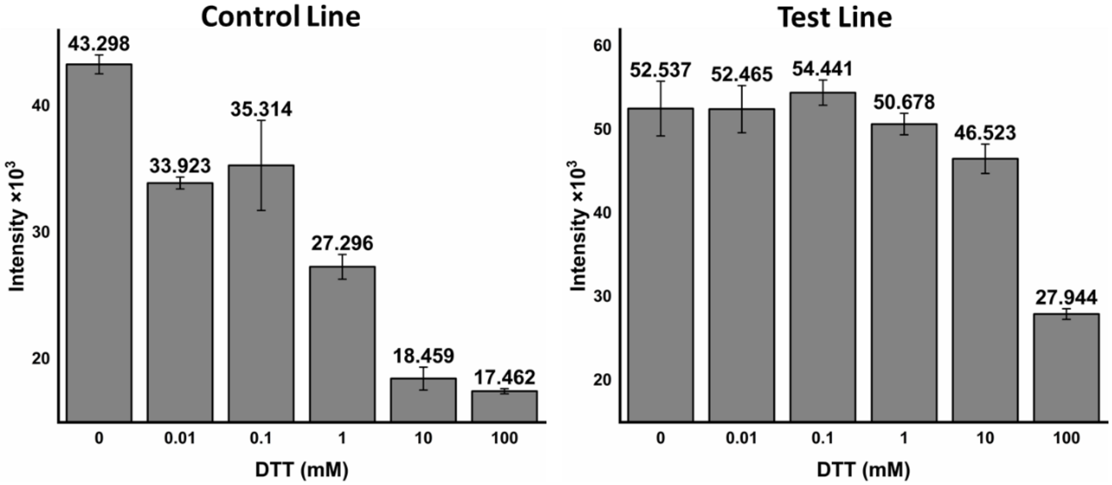

Figure S19. (a) LFA images and (b) quantitative analysis of the **Au@1** test line signals and the control line signals after incubating **Au@1** with various concentrations of dithiothreitol (DTT) for 1 hour.

(a)

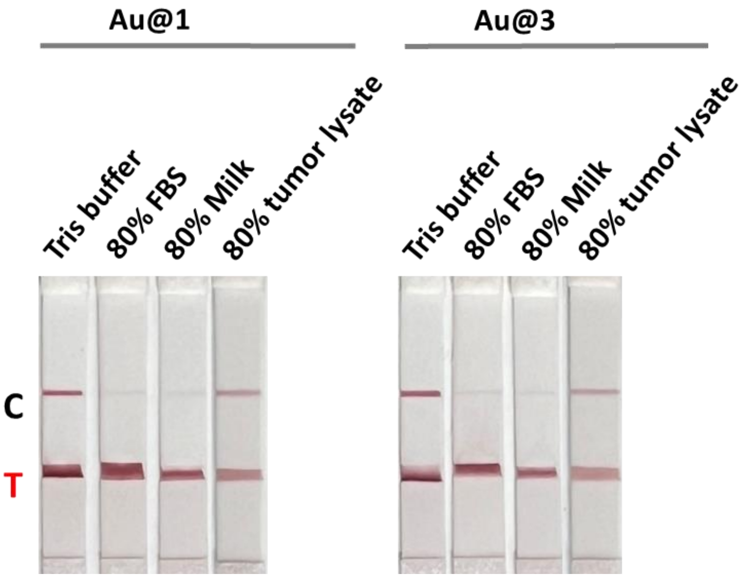

(b)

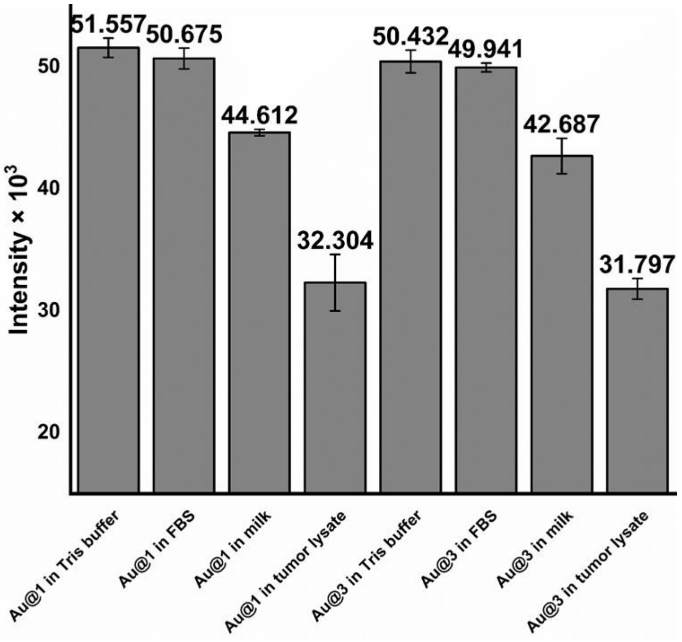

Figure S20. (a) LFA images and (b) quantitative analysis of the **Au@1** and Au@3 test line signals after incubating the nanoparticles in the medium at 37°C for one hour.

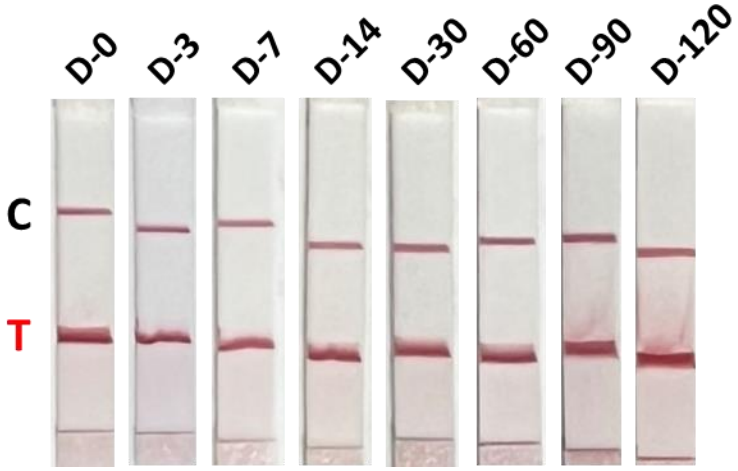

Figure S21. Stability test of **Au@1** stored in Tris buffer at 4 °C from day 0 to day 120.

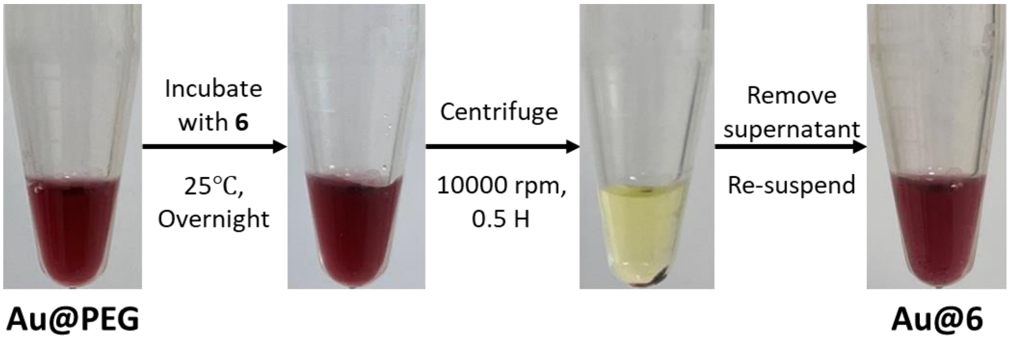

Figure S22. Images and steps for the preparation of fluorescein-functionalized gold nanoparticles **Au@6**.

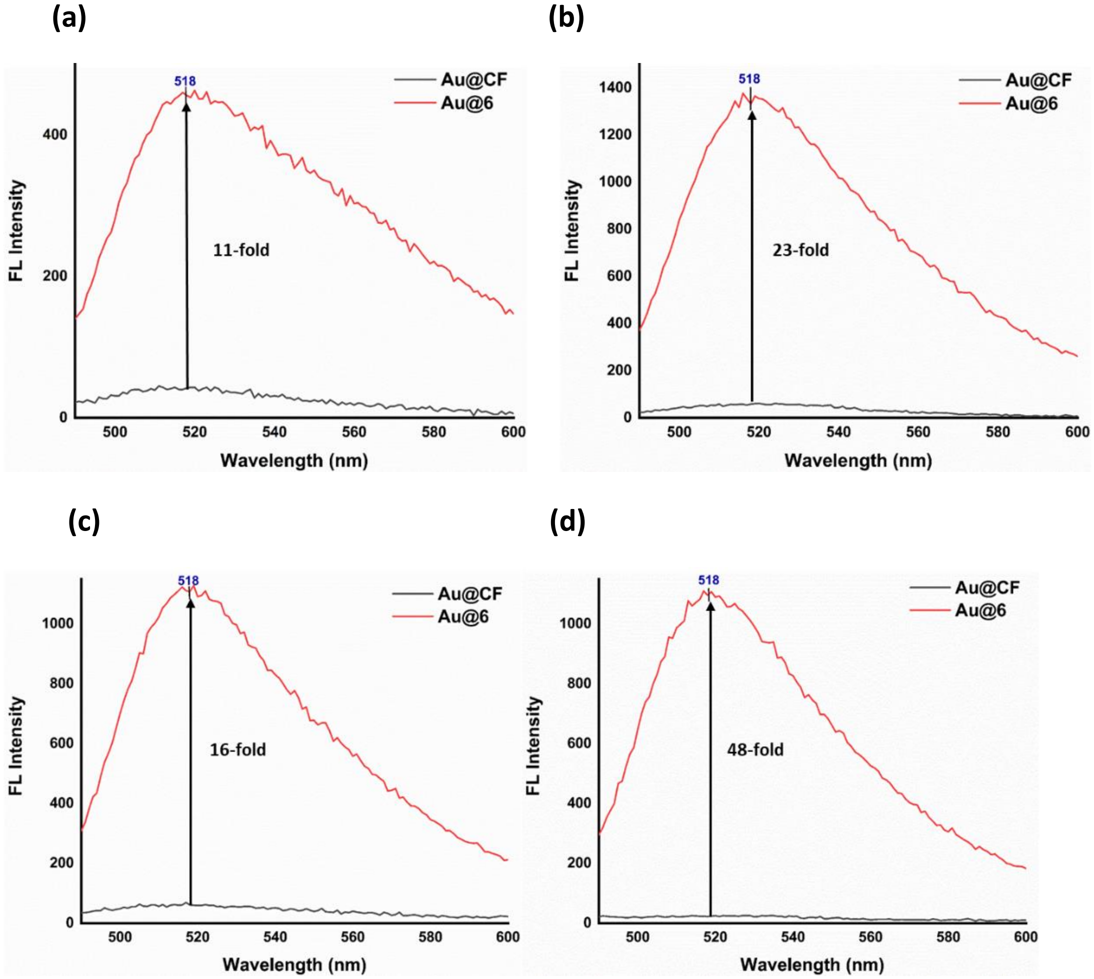

Figure S23. Reactions of 25 μM **6** with **Au@PEG** that are capped by using different polyethylene glycols. Fluorescent spectra of **Au@CF** and **Au@6** in which the capping polyethylene glycols for **Au@6** are (a) SH-PEG(2k)-OMe, (b) SH-PEG(2k)-NH_2, (c) SH-PEG(6k)-OMe, and (d) SH-$CH_2CH_2(CH_2CH_2O)_2$-OMe. 2k = 2000 Da, 6k = 6000 Da.

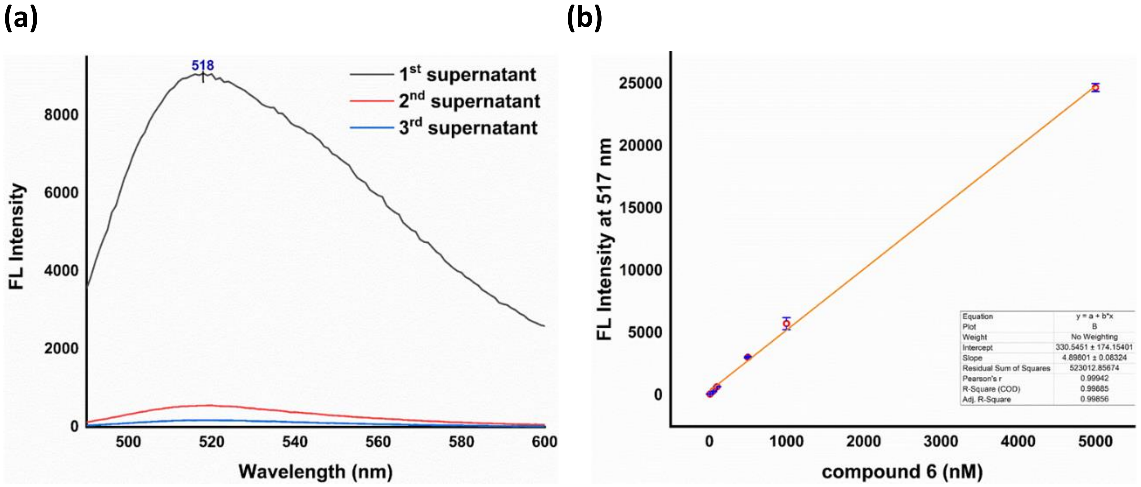

Figure S24. (a) Fluorescent spectra of the unreacted compound **6** after the reaction of 25 μM **6** with **Au@PEG**. Complete removal of **6** can be achieved with three cycles of centrifugations. (b) Calibration curve of **6**.

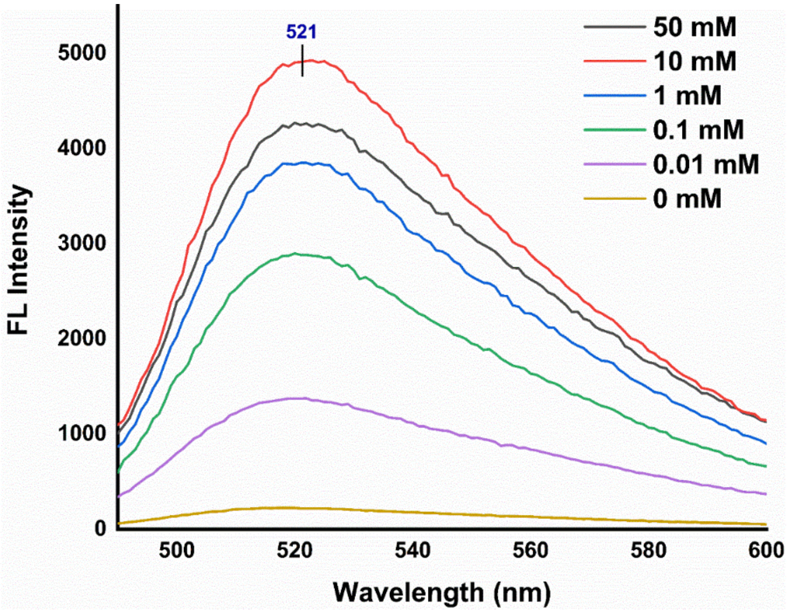

Figure S25. Fluorescent spectra of the released compound **6** from **Au@6** upon incubation with different concentrations of DTT for 1 hour at 25 °C.

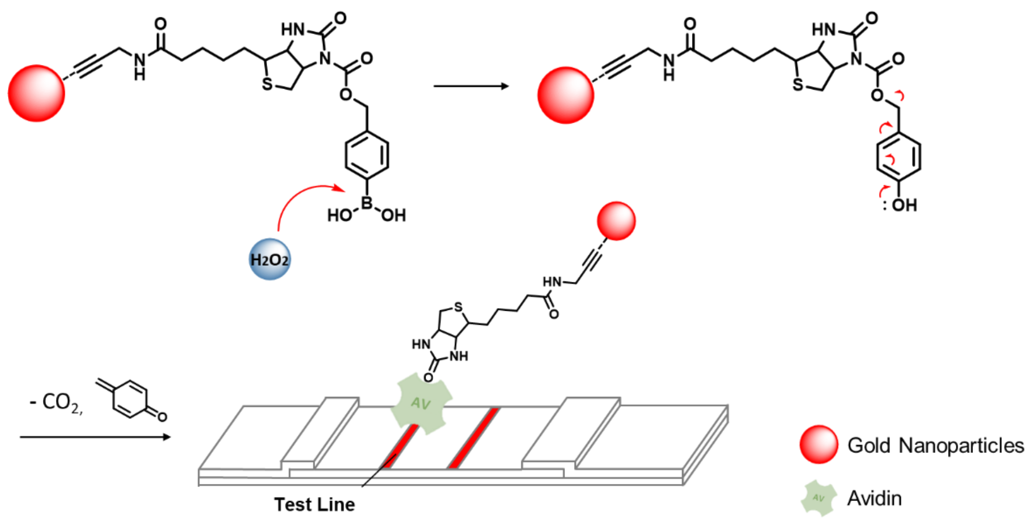

Figure S26. Schematic illustration of affinity-switchable lateral flow assay (ASLFA) strategy employing **Au@7** for the rapid detection of H_2O_2.

(a)

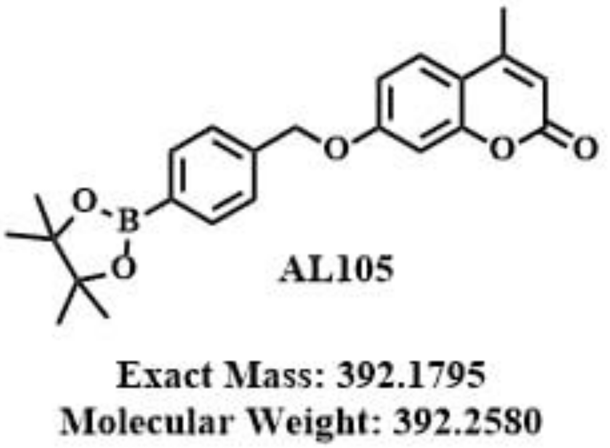

(b)

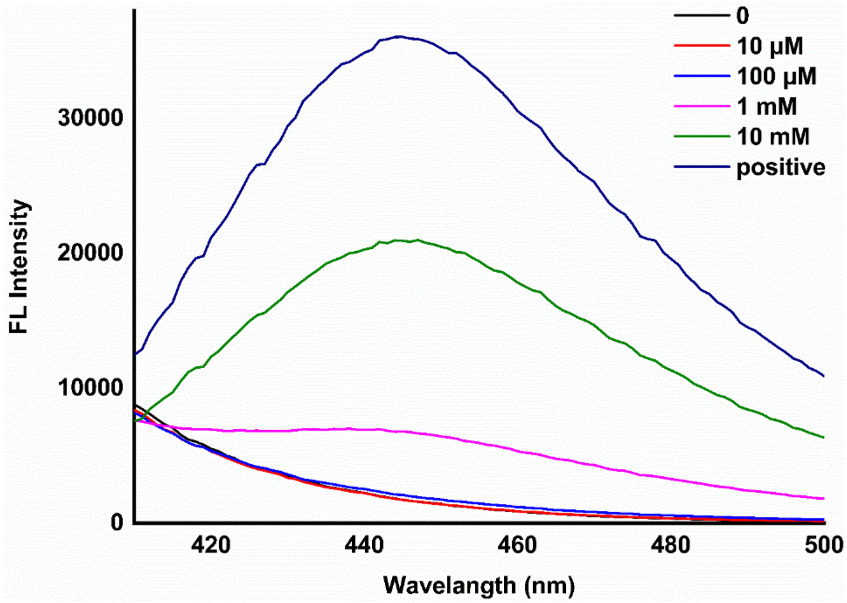

Figure S27. Detection of H_2O_2 using fluorescent turn-on chemical probe **AL105** (a) Chemical structure of **AL105**. (b) Fluorescent spectra of **AL105** upon reaction with different concentrations of H_2O_2 for 1 hour at 37 °C.

(a)

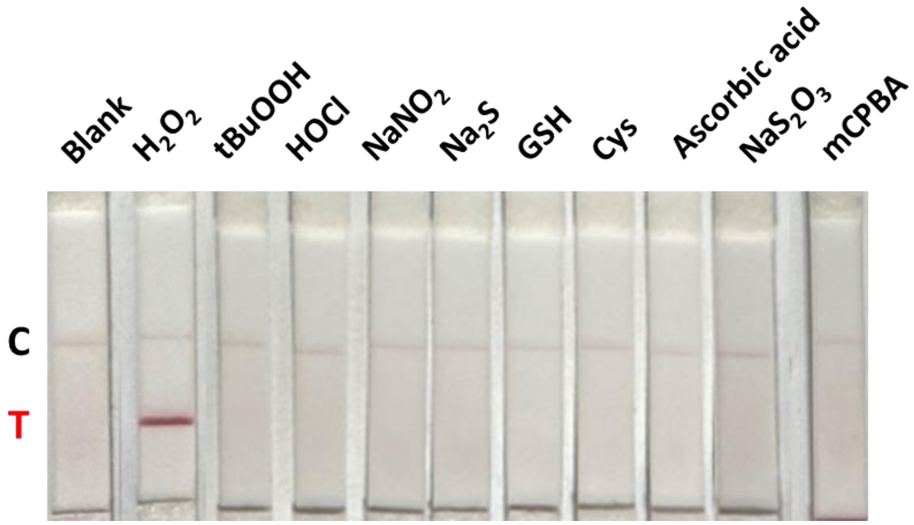

(b)

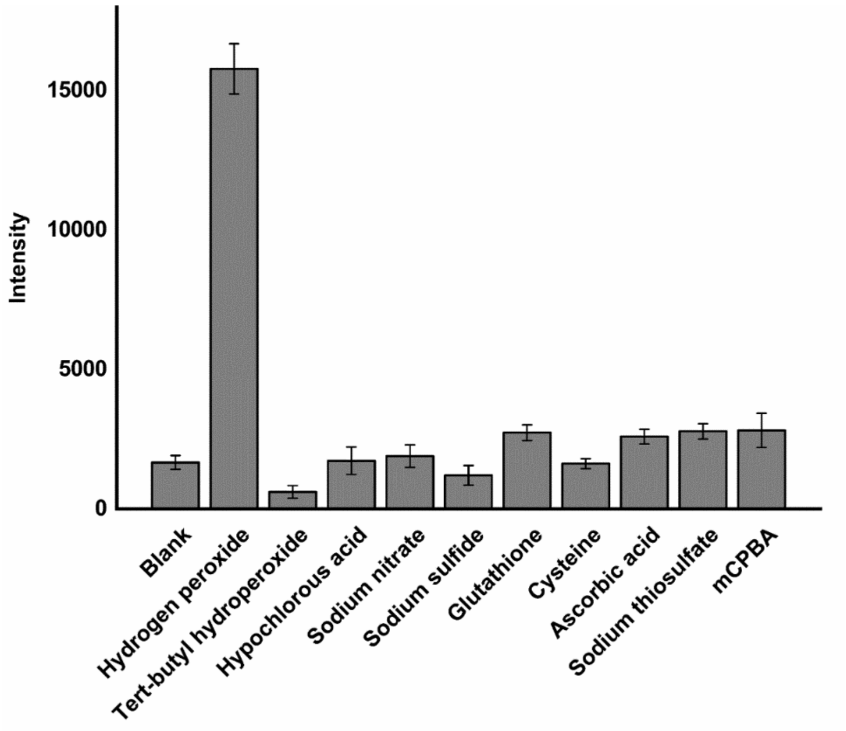

Figure S28. Reaction of **Au@7** with different oxidants and reductants at 100 μM. (a) LFA images and (b) quantitative analysis of the test line signals.

(a) (b)

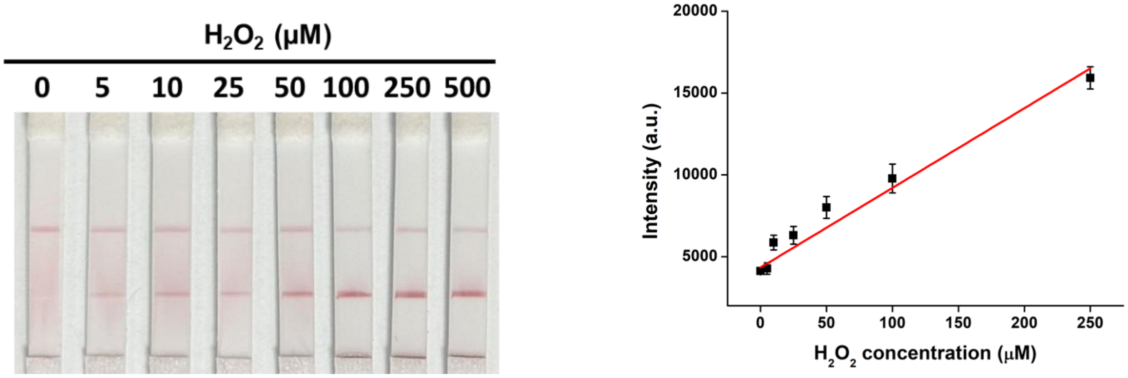

Figure S29. (a) Lateral flow assay images (b) quantitative analysis of the H_2O_2 detection in 50% FBS by using **Au@7**.

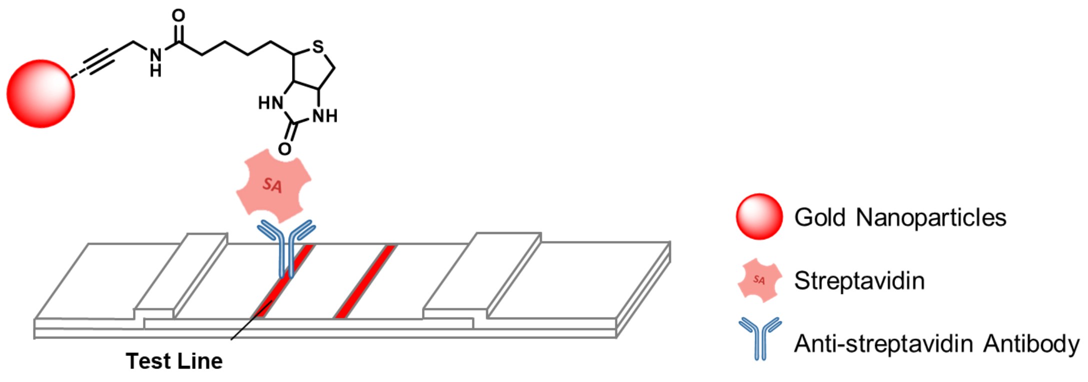

Figure S30. Schematic illustration of sandwich-type LFA strategy for the detection of streptavidin protein using **Au@1** and anti-streptavidin antibody.

(a)

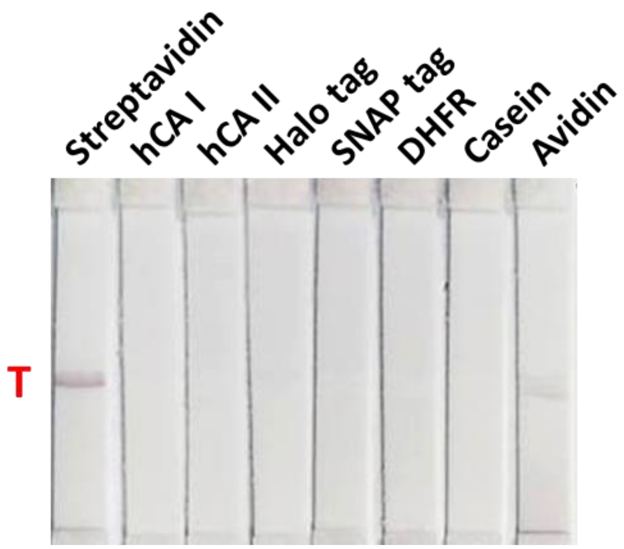

(b)

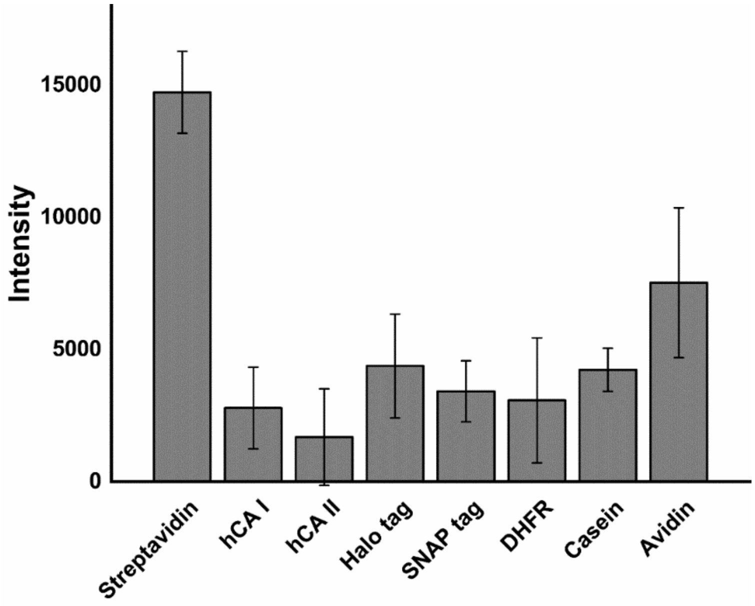

Figure S31. (a) LFA images and (b) quantitative analysis of the test line signals after protein testing using **Au@1**.

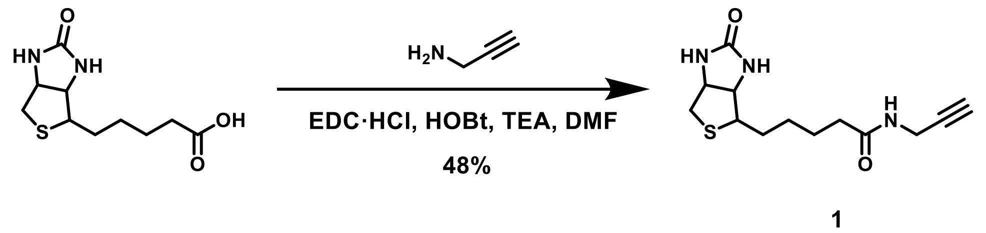

Scheme S1. Synthesis of Compound **1**.

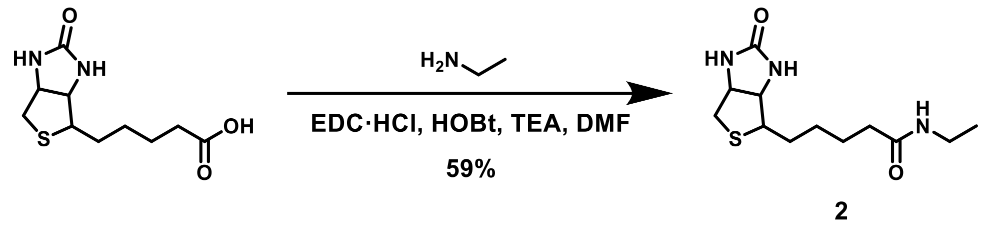

Scheme S2. Synthesis of Compound **2**.

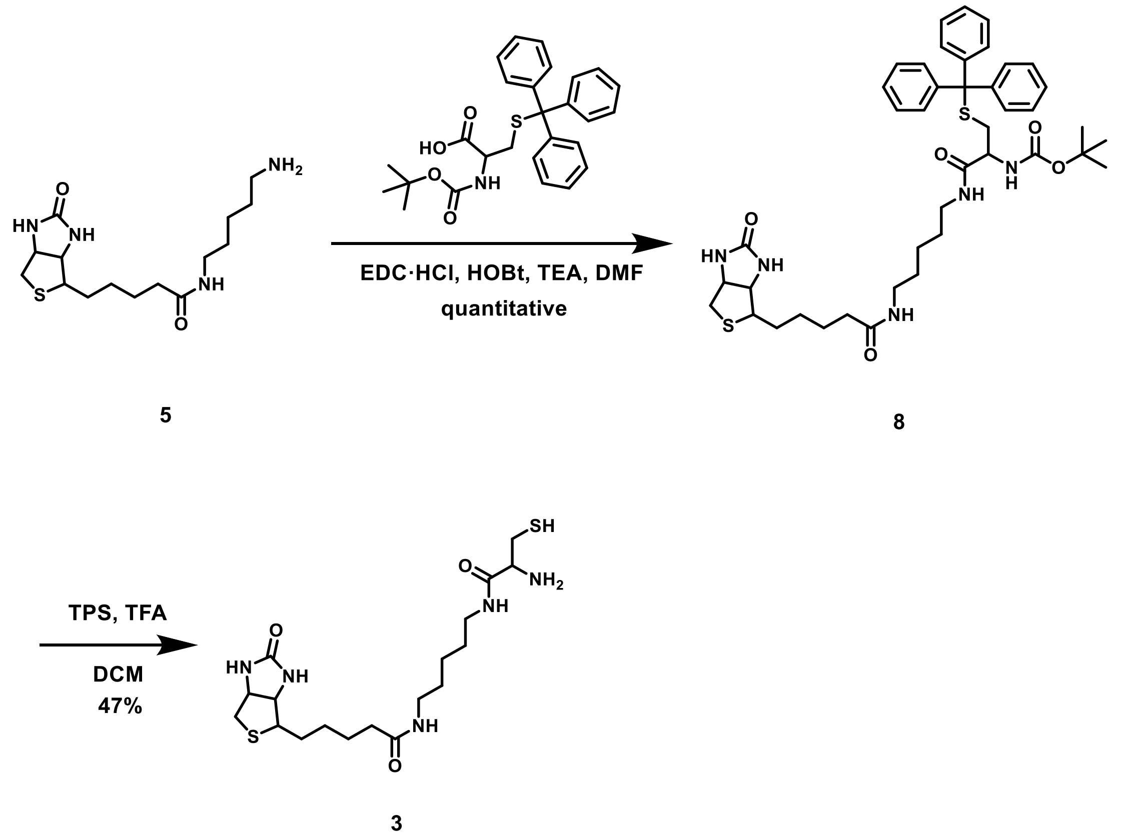

Scheme S3. Synthesis of Compound **3**.

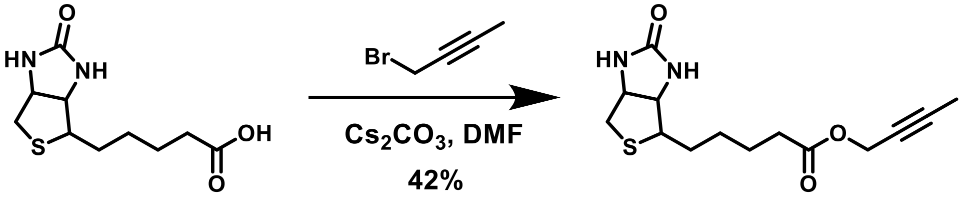

4

Scheme S4. Synthesis of Compound **4**.

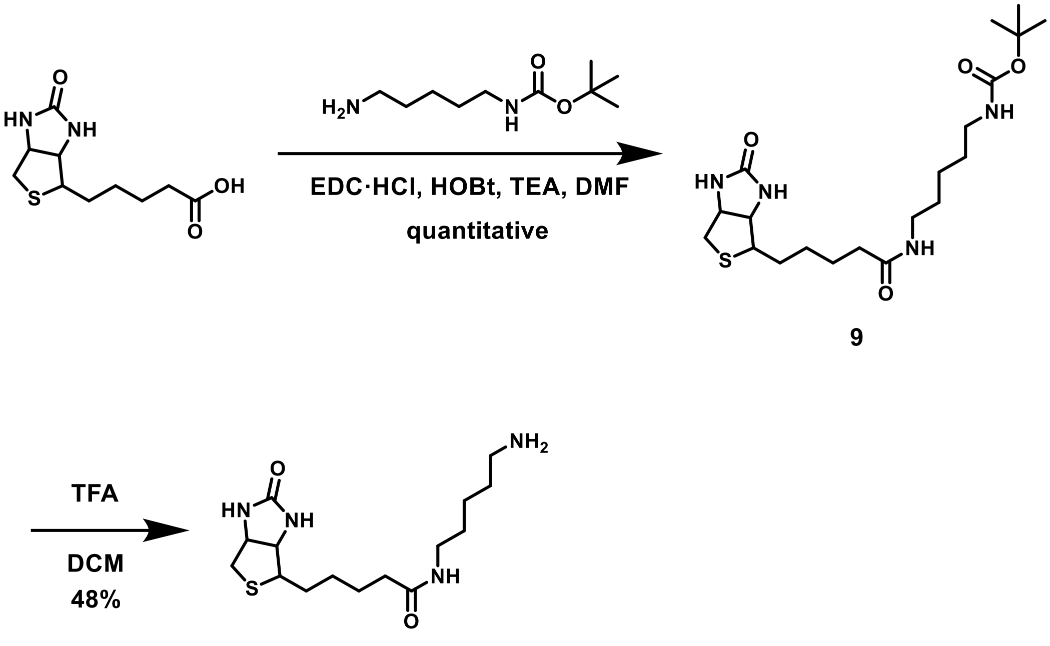

5

Scheme S5. Synthesis of Compound **5**.

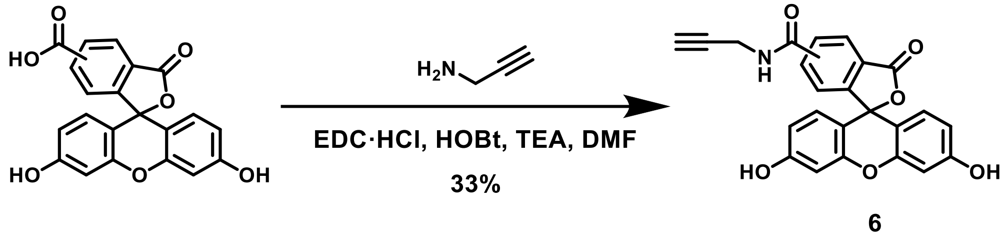

Scheme S6. Synthesis of Compound **6**.

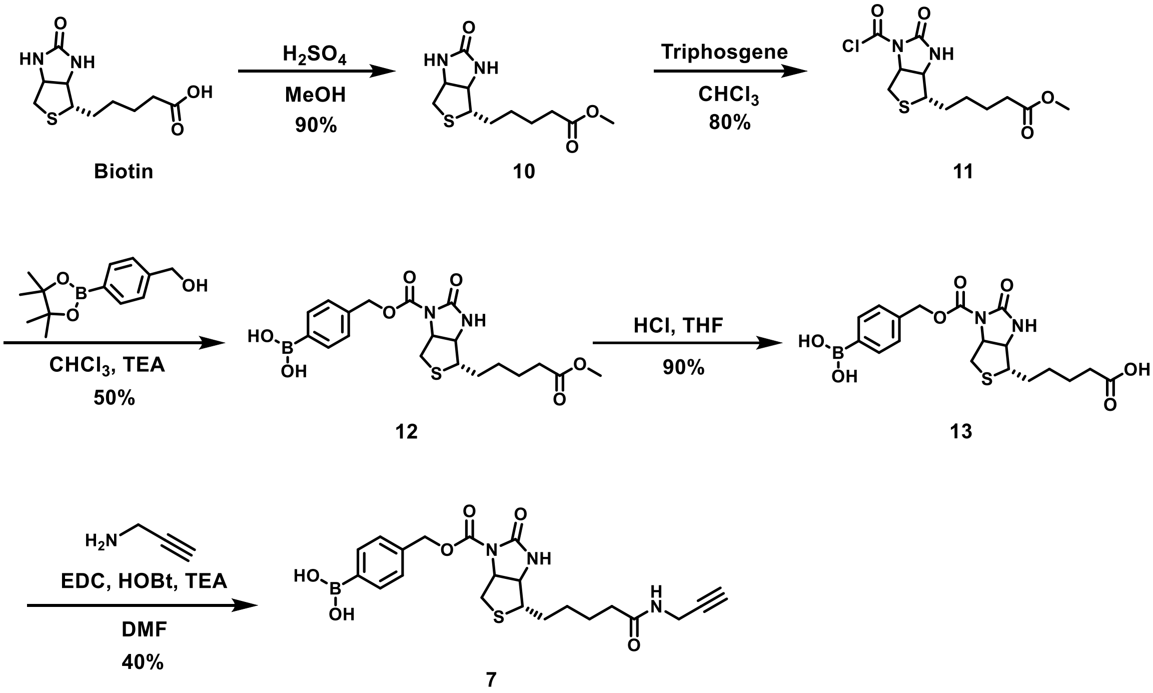

Scheme S7. Synthesis of Compound **7**.

Synthesis of Compound 1

To D(+)-Biotin (50 mg, 0.2 mmol) was added DMF (2mL), TEA (84 µL, 0.6 mmol), EDC·HCl (57 mg, 0.3 mmol), HOBt (40 mg, 0.3mmol) sequentially at room temperature, and stir for 15 minutes. Propargylamine (26 µL, 0.4 mmol) was added into the mixture and stirred at room temperature. The reaction was monitored by HPLC. After the reaction was complete, DMF was removed under reduced pressure. The solid mass was then dissolved DCM (4 mL) and was successively wash with saturated aqueous $NaCO_3$ (5mL), 3N HCl (5mL), and finally dry over anhydrous Na_2SO_4. The organic solvent was removed by rotary evaporation. Subsequently, the residue was purified by reverse-phase column chromatography (gradient 10% ACN - 90% H_2O to 100% ACN for 21 min, detected at 8.8 min) to afford compound **1** as a yellow solid in 48% yield (27.0 mg). **^{1}H NMR** (700 MHz, CD_3OD) δ 4.49 (dd, *J* = 7.9, 4.4 Hz, 1H), 4.31 (dd, *J* = 7.9, 4.4 Hz, 1H), 3.94 (d, *J* = 2.6 Hz, 2H), 3.22-3.14 (m, 1H), 2.93 (dd, *J* = 12.7, 5.0 Hz, 1H), 2.70 (d, *J* = 12.7 Hz, 1H), 2.58 (t, *J* = 2.6 Hz, 1H), 2.22 (td, *J* = 7.5, 2.6 Hz, 2H), 1.77 – 1.57 (m, 4H), 1.48 – 1.40 (m, 2H) ppm. **^{13}C NMR** (176 MHz, CD_3OD) δ 175.65, 166.14, 80.64, 72.10, 63.37, 61.64, 56.97, 41.04, 36.47, 29.72, 29.45, 29.36, 26.70 ppm. **HRMS** (ESI): m/z calc. for $[C_{13}H_{19}N_3O_2S_1Na_1]^+$ $[M+Na]^+$ 304.10957 found 304.10889

Synthesis of Compound 2

To D(+)-Biotin (50 mg, 0.2 mmol) was added DMF (3mL), TEA (84 µL, 0.6 mmol), EDC·HCl (57 mg, 0.3 mmol), HOBt (40 mg, 0.3 mmol) sequentially at room temperature, and stir for 15 minutes. Ethylamine (26 µL, 0.4 mmol) was added into the mixture and stirred at room temperature. The reaction was monitored by HPLC. After the reaction was complete, DMF was removed under reduced pressure. The solid mass was then dissolved DCM (4

mL) and was successively wash with saturated aqueous $NaCO_3$ (5mL), 3N HCl (5mL), and finally dry over anhydrous Na_2SO_4. The organic solvent was removed by rotary evaporation. Subsequently, the residue was purified by reverse-phase column chromatography (gradient 10% ACN- 90% H_2O to 100% ACN for 21 min, detected at 8.6 min) to afford compound **2** as a primrose yellow solid in 59% yield (32.0 mg). **^{1}H NMR** (700 MHz, CD_3OD) δ 4.49 (dd, *J* = 7.9, 5.0 Hz, 1H), 4.31 (dd, *J* = 7.9, 5.0 Hz, 1H), 3.26 – 3.14 (m, 3H), 2.93 (dd, *J* = 12.7, 5.0 Hz, 1H), 2.71 (d, *J* = 12.7 Hz, 1H), 2.18 (td, *J* = 7.5, 2.4 Hz, 2H), 1.77 – 1.70 (m, 1H), 1.70 – 1.56 (m, 3H), 1.47 – 1.40 (m, 2H), 1.11 (t, *J* = 7.3 Hz, 3H) ppm. **^{13}C NMR** (176 MHz, CD_3OD) δ 175.81, 166.11, 63.37, 61.63, 56.98, 41.03, 36.81, 35.18, 29.76, 29.48, 26.88, 14.83 ppm. **HRMS** (ESI): m/z calc. for $[C_{12}H_{21}N_3O_2S_1Na_1]^+$ $[M+Na]^+$ 294.12522 found 294.12534.

Synthesis of Compound 8

To *N*-(*tert*-Butoxycarbonyl)-*S*-trityl-L-cysteine (32 mg, 0.07 mmol) was added DMF (2mL), TEA (41 μL, 0.3 mmol), EDC·HCl (19 mg, 0.1 mmol), HOBt (13 mg, 0.1 mmol) sequentially at room temperature, and stirred for 15 minutes. Compound **2** (23 mg, 0.07 mmol) was dissolved in 1mL of DMF, and dropped into the above solution at room temperature. The reaction was monitored by HPLC. After the reaction was complete, DMF and TEA were removed under reduced pressure to afford compound **8** which was used in the next step without further purification.

Synthesis of Compound 3

To Compound **7** (12 mg, 0.015 mmol) was added DCM (1mL), TFA (1mL), TPS (9μL, 0.045mmol) sequentially at room temperature, and stirred for 2 hours. The bright yellow

solution progressively became limpid. After the reaction appeared complete, the solvents were removed under reduced pressure. The crude mixture was purified by reverse-phase column chromatography (gradient 10% ACN- 90% H_2O to 100% ACN for 21 min, detected at 8.6 min) to afford compound **3** as a limpid liquid in 47% yield (4.2 mg). **^{1}H NMR** (700 MHz, CD_3OD) δ 4.50 (dd, *J* = 7.9, 4.5 Hz, 1H), 4.31 (dd, *J* = 7.9, 4.5 Hz, 1H), 3.93 (dd, *J* = 7.0, 5.1 Hz, 1H), 3.28 – 3.20 (m, 3H), 3.17 (t, *J* = 7.0 Hz, 2H), 3.03 (dd, *J* = 14.6, 5.2 Hz, 1H), 2.96 – 2.90 (m, 2H), 2.71 (d, *J* = 12.8 Hz, 1H), 2.20 (t, *J* = 7.3 Hz, 2H), 1.68 – 1.51 (m, 8H), 1.48 – 1.42 (m, 2H), 1.41 – 1.34 (m, 3H) ppm. **^{13}C NMR** (176 MHz, CD_3OD) δ 176.06, 168.17, 166.14, 63.37, 61.63, 57.01, 56.23, 41.05, 40.61, 40.07, 36.80, 30.09, 29.85, 29.74, 29.50, 26.94, 26.34, 25.18 ppm. **HRMS** (ESI): m/z calc. for $[C_{18}H_{34}N_5O_3S_2]^+$ $[M+H]^+$ 432.21031 found 432.21040.

Synthesis of Compound 4

D(+)-Biotin (50 mg, 0.2 mmol) was dissolved in 3 mL of DMF. Subsequently, cesium carbonate (98 mg, 0.3 mmol) was added to the solution, and the reaction of mixture was stirred at room temperature for 10 minutes. 1-Bromo-2-butyne (53 µL, 0.6 mmol) was added into the mixture and stirred at room temperature. After 18h, DMF was removed under reduced pressure. Purification was performed by reverse-phase column chromatography (gradient 10% ACN- 90% H_2O to 100% ACN for 21 min, detected at 12.4 min) to afford compound **4** as a white solid in 42% yield (24.9 mg).

^{1}H NMR (700 MHz, $CDCl_3$) δ 5.99 (s, 1H), 5.51 (s, 1H), 4.61 (q, *J* = 2.4 Hz, 2H), 4.48 (dd, *J* = 8.0, 5.0 Hz, 1H), 4.28 (dd, *J* = 8.0, 5.0 Hz, 1H), 3.16 – 3.09 (m, 1H), 2.88 (dd, *J* = 12.8, 5.0 Hz, 1H), 2.72 (d, *J* = 12.8 Hz, 1H), 2.34 (t, *J* = 7.6 Hz, 2H), 1.83 (t, *J* = 2.4 Hz, 3H), 1.74 – 1.60 (m, 4H), 1.48 – 1.36 (m, 2H) ppm. **^{13}C NMR** (176 MHz, $CDCl_3$) δ 173.00, 163.72, 83.15,

73.17, 61.93, 60.11, 55.43, 52.69, 40.50, 33.66, 28.27, 28.18, 24.64, 3.62 ppm. **HRMS** (ESI): m/z calc. for $[C_{14}H_{20}N_2O_3S_1Na_1]^+$ $[M+Na]^+$ 319.10923 found 319.10893.

Synthesis of Compound 9

To D(+)-Biotin (30 mg, 0.13 mmol) was added DMF (1mL), TEA (55 µL, 0.4 mmol), EDC·HCl (38 mg, 0.2 mmol), HOBt (27 mg, 0.2 mmol) sequentially at room temperature, and stirred for 15 minutes. *N*-Boc-cadaverine (28 µL, 0.13 mmol) was added into the mixture and stirred at room temperature. The reaction was monitored by HPLC. After the reaction was complete, DMF and TEA were removed under reduced pressure to afford compound **9** which was used in the next step without further purification.

Synthesis of Compound 5

Compound **9** (42 mg, 0.10 mmol) was dissolved in 2.4 mL of DCM. Trifluoroacetic acid (240 µL, 3.13 mmol) was added to the solution, and the reaction of mixture was stirred at room temperature. After 2 hours, the completion of the reaction was detected by TLC (MeOH/DCM= 1:2, R_f = 0.5). The reaction was then diluted to 3 mL with toluene and evaporated to dryness. Subsequently, the residue was purified by reverse-phase column chromatography (gradient 10% ACN- 90% H_2O to 100% ACN for 21 min, detected at 7.6 min) to afford compound **5** as primrose yellow liquid in 48% yield (16.0 mg). **^{1}H NMR** (700 MHz, CD_3OD) δ 4.48 (dd, *J* = 7.9, 5.0 Hz, 1H), 4.28 (dd, *J* = 7.9, 5.0 Hz, 1H), 3.22 – 3.12 (m, 3H), 2.94 – 2.85 (m, 3H), 2.68 (d, *J* = 12.7 Hz, 1H), 2.18 (t, *J* = 7.4 Hz, 2H), 1.74 – 1.50 (m, 8H), 1.45 – 1.35 (m, 4H) ppm. **^{13}C NMR** (176 MHz, CD_3OD) δ 176.13, 166.13, 63.40, 61.62, 57.08, 41.05, 40.58, 39.87, 36.76, 29.93, 29.80, 29.52, 28.14, 26.90, 24.69 ppm. **HRMS** (ESI): m/z calc. for $[C_{15}H_{29}N_4O_2S_1]^+$ $[M+H]^+$ 329.20112 found 329.20094.

Synthesis of Compound 6

To 5(6)-carboxyfluorescein (20 mg, 0.05 mmol) was added DMF (1mL), TEA (22 µL, 0.16 mmol), EDC·HCl (15 mg, 0.08 mmol), HOBt (11 mg, 0.08 mmol) sequentially at room temperature, and stir for 15 minutes. Propargylamine (15 µL, 0.27 mmol) was added into the mixture and stirred at room temperature. The reaction was monitored by HPLC. After the reaction was complete, DMF was removed under reduced pressure. The solid mass was then dissolved DCM (3 mL) and was successively washed with saturated aqueous $NaCO_3$ (5mL), 3N HCl (5mL), and finally dry over anhydrous Na_2SO_4. The organic solvent was removed by rotary evaporation. Subsequently, the residue was purified by reverse-phase column chromatography (gradient 10% ACN- 90% H_2O to 100% ACN for 21 min, detected at 12.4 min) to afford compound **6** as a dark yellow solid in 33% yield (7.4 mg). **^{1}H NMR** (700 MHz, CD_3OD) δ 8.32 (dd, *J* = 1.7, 0.6 Hz, 3/5H), 8.02 (td, *J* = 7.8, 1.7 Hz, 1H), 7.99 (d, *J* = 8.0 Hz, 1/2H), 7.52 (s, 1/2H), 7.17 (d, *J* = 8.0 Hz, 3/5H), 6.64 (dd, *J* = 6.5, 2.2 Hz, 2H), 6.48 – 6.44 (m, 4H), 4.19 (d, *J* = 2.6 Hz, 2H), 3.91 (d, *J* = 2.6 Hz, 2H), 2.63 (t, *J* = 2.6 Hz, 1H), 2.16 (t, *J* = 2.6 Hz, 1H) ppm. **^{13}C NMR** (176 MHz, CD_3OD) δ 170.05, 168.51, 168.32, 167.91, 167.73, 160.53, 157.67, 155.21, 154.43, 154.36, 141.42, 140.01, 139.32, 137.45, 136.19, 135.17, 133.93, 133.74, 131.58, 130.65, 130.43, 130.31, 130.25, 129.87, 129.24, 128.20, 125.30, 124.27, 123.95, 123.07, 122.04, 120.67, 113.41, 113.37, 109.56, 108.20, 103.68, 103.58, 80.46, 80.27, 72.34, 72.25, 30.16, 30.06 ppm. **HRMS** (ESI): m/z calc. for $[C_{24}H_{14}N_1O_6]^-$ $[M–H]^-$ 412.08211 found 412.08210.

Synthesis of Compound 7

Mixture of compound **13** (20 mg, 0.047 mmol), HOBt (19 mg, 0.14 mmol), EDC.HCl (27 mg, 0.14 mmol), TEA (14 mg, 0.14 mmol) in 6 mL DMF was stirred for 5 minutes in a reaction

flask. Propargylamine (8 mg, 0.14 mmol) was added, and the reaction mixture was reacted overnight. DMF was removed under reduced pressure. The crude mixture was purified by reverse-phase column chromatography (ACN/H_2O) to afford compound **7** as a white solid in 40 % yield (9 mg). **^{1}H NMR** (500 MHz, d-DMSO): δ8.22 (t, *J* = 5.5 Hz, 1H), 7.78 (d, *J* = 7.7 Hz, 1H), 7.71 (s, 1H), 7.36 (d, *J* = 7.7 Hz, 1H), 5.20 (ABq, *J* = 12.9 Hz, 2H), 4.78 – 4.75 (m, 1H), 4.13 (dd, *J* = 8.1, 4.2 Hz, 1H), 3.83 (dd, *J* = 5.5, 2.6 Hz, 2H), 3.20 – 3.16 (m, 1H), 3.07 (t, *J* = 2.5 Hz, 1H), 3.0 – 2.9 (m, 2H), 2.08 (t, *J* = 7.5 Hz, 2H), 1.66 – 1.23 (m, 6H) ppm. **^{13}C NMR** (126 MHz, d-DMSO) δ 171.80, 154.64, 151.12, 137.78, 134.16, 126.50, 81.34, 72.78, 66.66, 61.93, 59.52, 57.22, 54.81, 38.05, 34.82, 28.13, 27.80, 27.71, 25.02 ppm. **HRMS** (ESI): m/z calc. for $[C_{21}H_{26}B_1N_3O_6S_1Na_1]^+$ $[M+Na]^+$ 482.15331 found 482.15330.

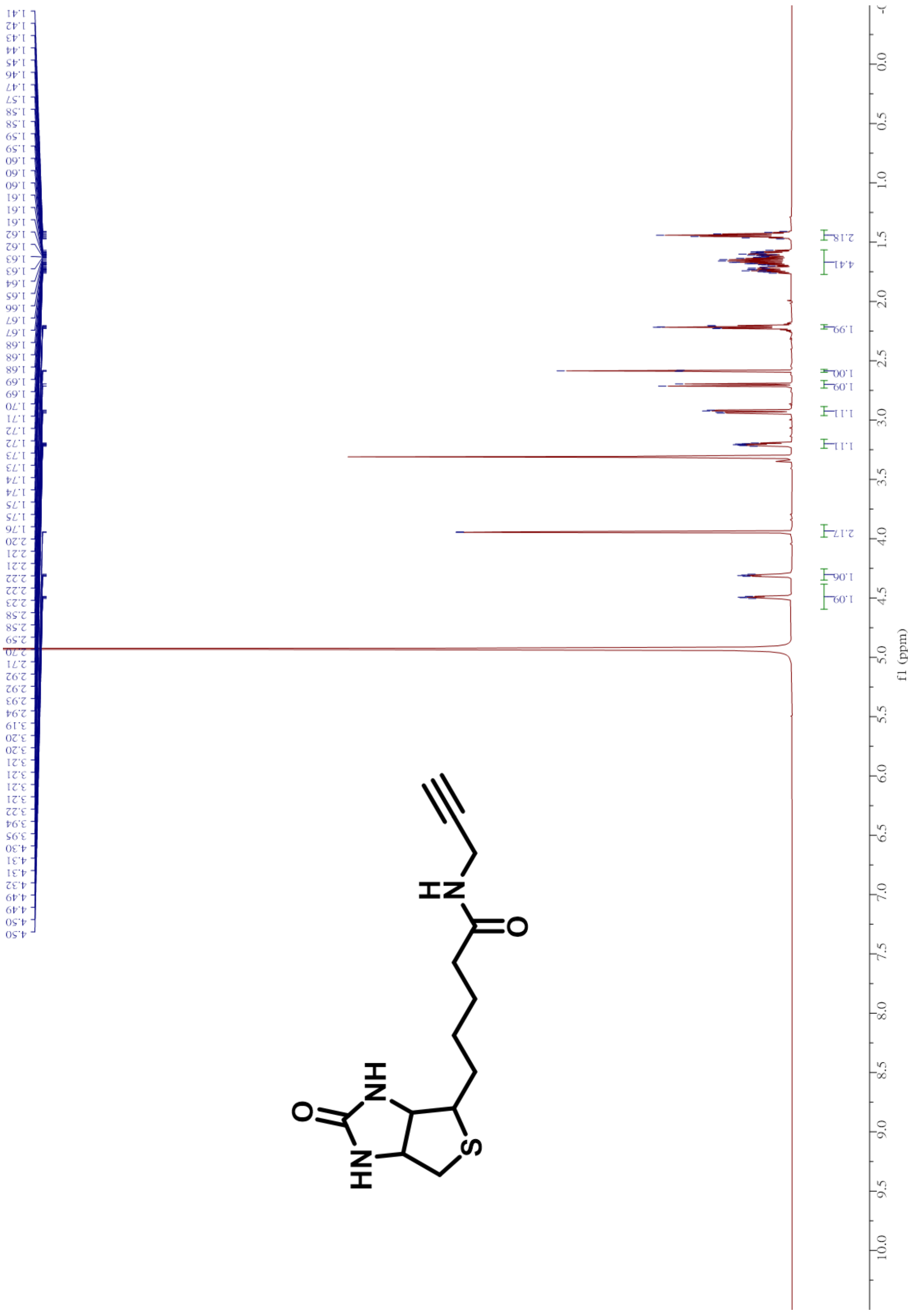
f1 (ppm)

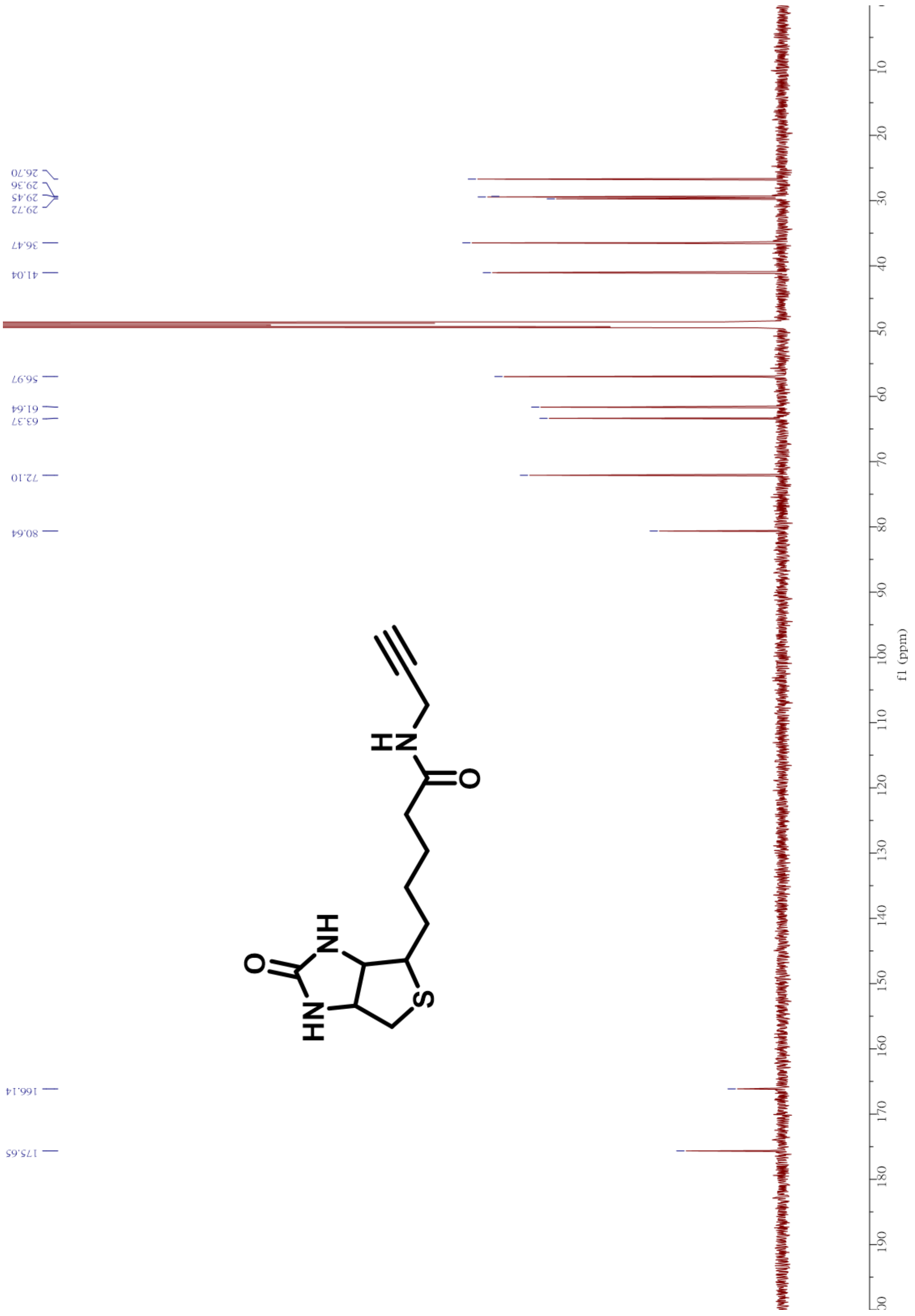
26.70
29.36
29.45
29.72
36.47
41.04
56.97
61.64
63.37
72.10
80.64
166.14
175.65
f1 (ppm)

Data:CY104
Comment:
Description:
Ionization Mode:ESI+
History:Average(MS[1] 0.25..0.33)

Acquired:9/11/2023 2:11:48 PM
Operator:AccuTOF
m/z Calibration File:20230901-TFANa_...
Created:9/11/2023 2:39:52 PM
Created by:AccuTOF

Charge number:1
Tolerance:500.00[ppm], 500.00 .. 500....
Unsaturation Number:-300.5 .. 300.0 (...
Element:^{12}C:13 .. 13, ^{1}H:0 .. 20, ^{14}N:3 .. 3, ^{23}Na:0 .. 2, ^{16}O:2 .. 2, ^{32}S:1 .. 1

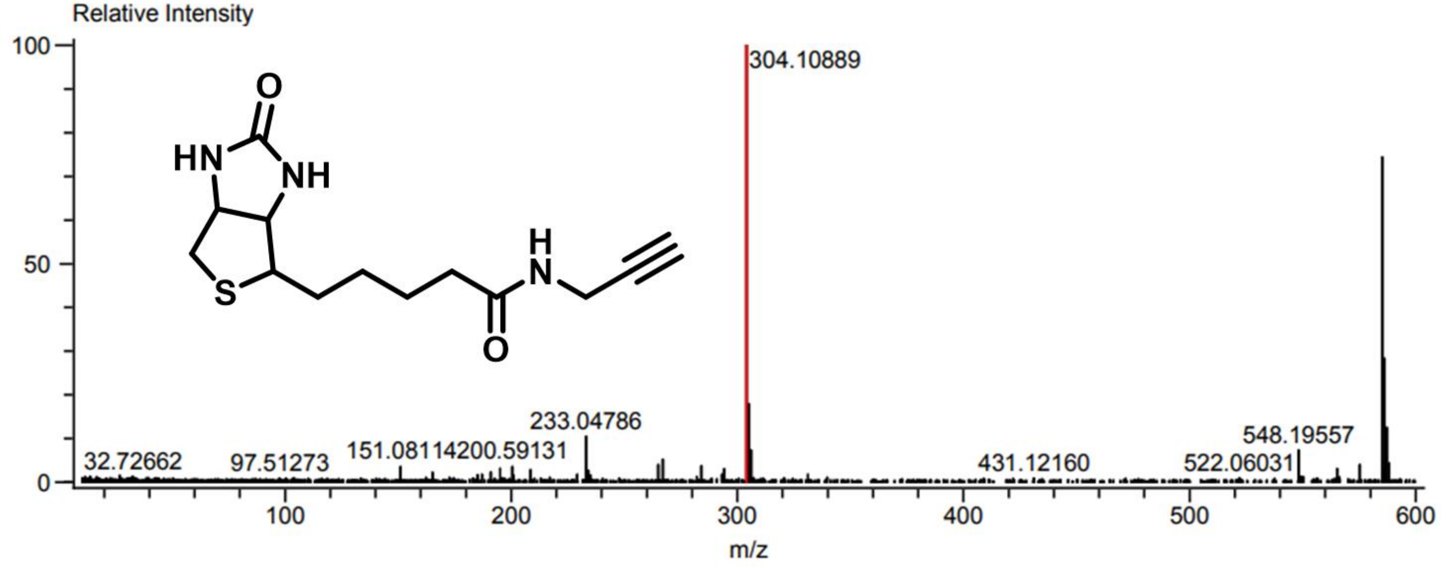

Mass	Intensity	Calc. Mass	Mass Difference [mDa]	Mass Difference [ppm]	Possible Formula
304.10889	20545.74	304.10957	-0.67	-2.22	$^{12}C_{13}{}^{1}H_{19}{}^{14}N_{3}{}^{23}Na_{1}{}^{16}O_{2}{}^{32}S_{1}$

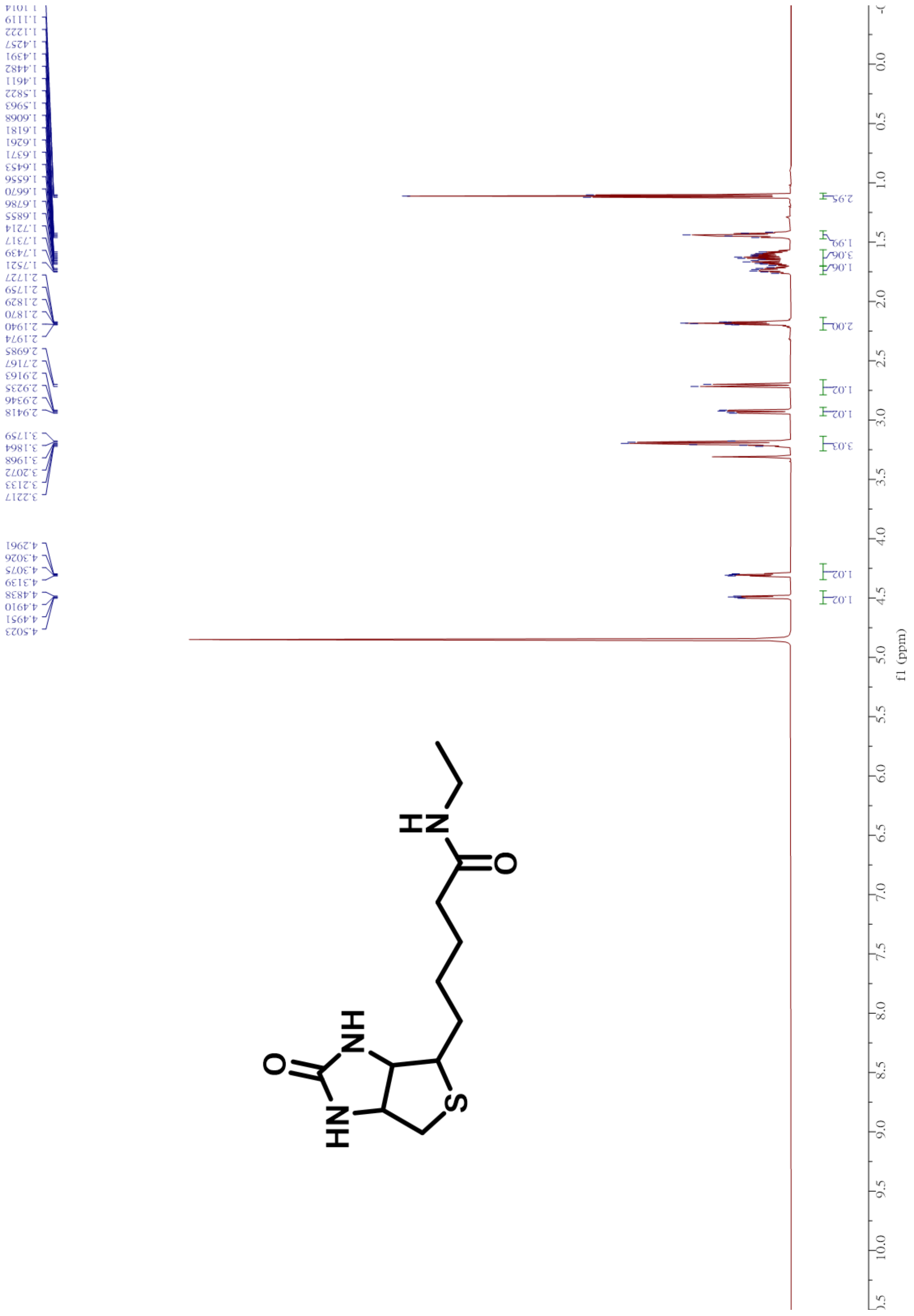
1.1014
1.1119
1.1222
1.4257
1.4391
1.4482
1.4611
1.5822
1.5963
1.6068
1.6181
1.6261
1.6371
1.6453
1.6556
1.6670
1.6786
1.6855
1.7214
1.7317
1.7439
1.7521
2.1727
2.1759
2.1829
2.1870
2.1940
2.1974
2.6985
2.7167
2.9163
2.9235
2.9346
2.9418
3.1759
3.1864
3.1968
3.2072
3.2133
3.2217
4.2961
4.3026
4.3075
4.3139
4.4838
4.4910
4.4951
4.5023
2.95
1.99
3.06
1.06
2.00
1.02
1.02
3.03
1.02
1.02
f1 (ppm)

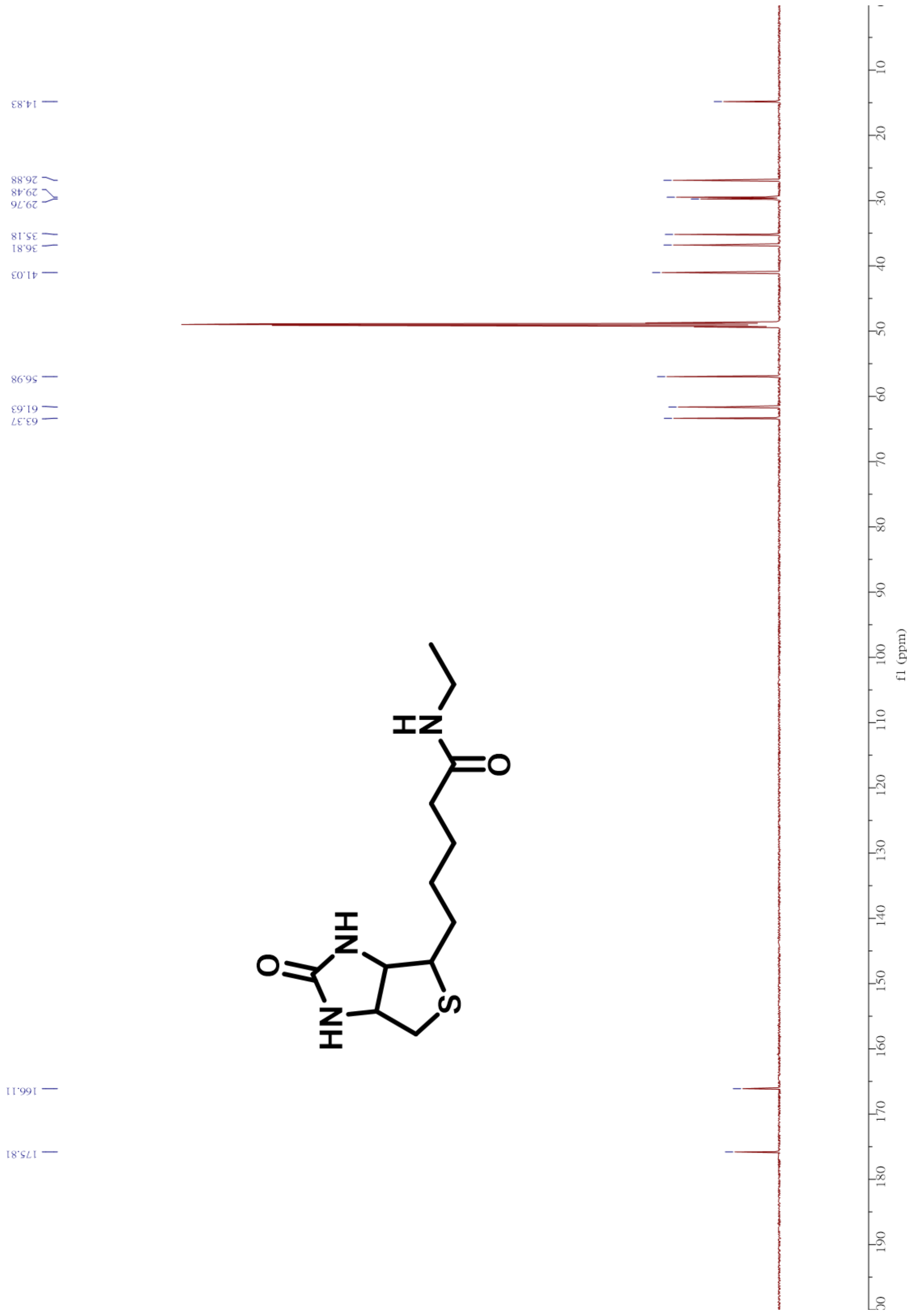

14.83
26.88
29.48
29.76
35.18
36.81
41.03
56.98
61.63
63.37
166.11
175.81
f1 (ppm)
O
HN
NH
S
O
N
H

Data:CY107
Comment:
Description:
Ionization Mode:ESI+
History:Average(MS[1] 0.34..0.39)

Acquired:9/11/2023 2:15:05 PM
Operator:AccuTOF
m/z Calibration File:20230901-TFANa_...
Created:9/11/2023 2:44:40 PM
Created by:AccuTOF

Charge number:1
Tolerance:500.00[ppm], 500.00 .. 500....
Unsaturation Number:-300.5 .. 300.0 (...
Element:^{12}C:12 .. 12, ^{1}H:0 .. 22, ^{14}N:3 .. 3, ^{23}Na:0 .. 2, ^{16}O:2 .. 2, ^{32}S:1 .. 1

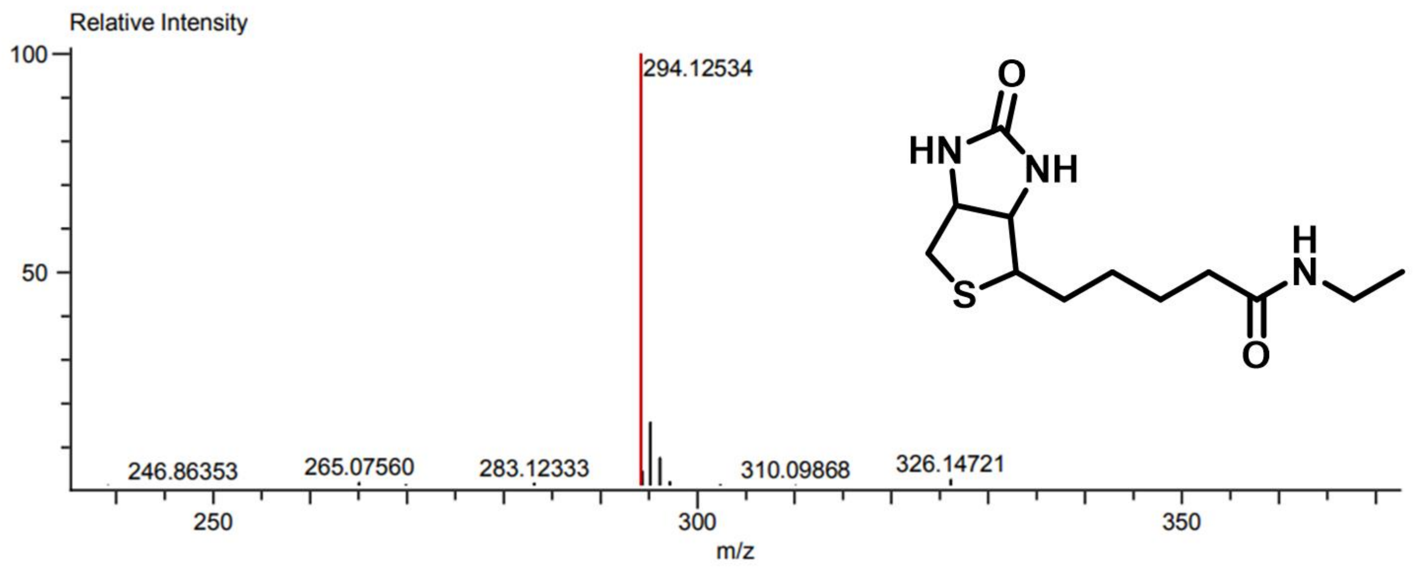

Mass	Intensity	Calc. Mass	Mass Difference [mDa]	Mass Difference [ppm]	Possible Formula
294.12534	28540.68	294.12522	0.13	0.44	$^{12}C_{12}{}^{1}H_{21}{}^{14}N_{3}{}^{23}Na_{1}{}^{16}O_{2}{}^{32}S_{1}$

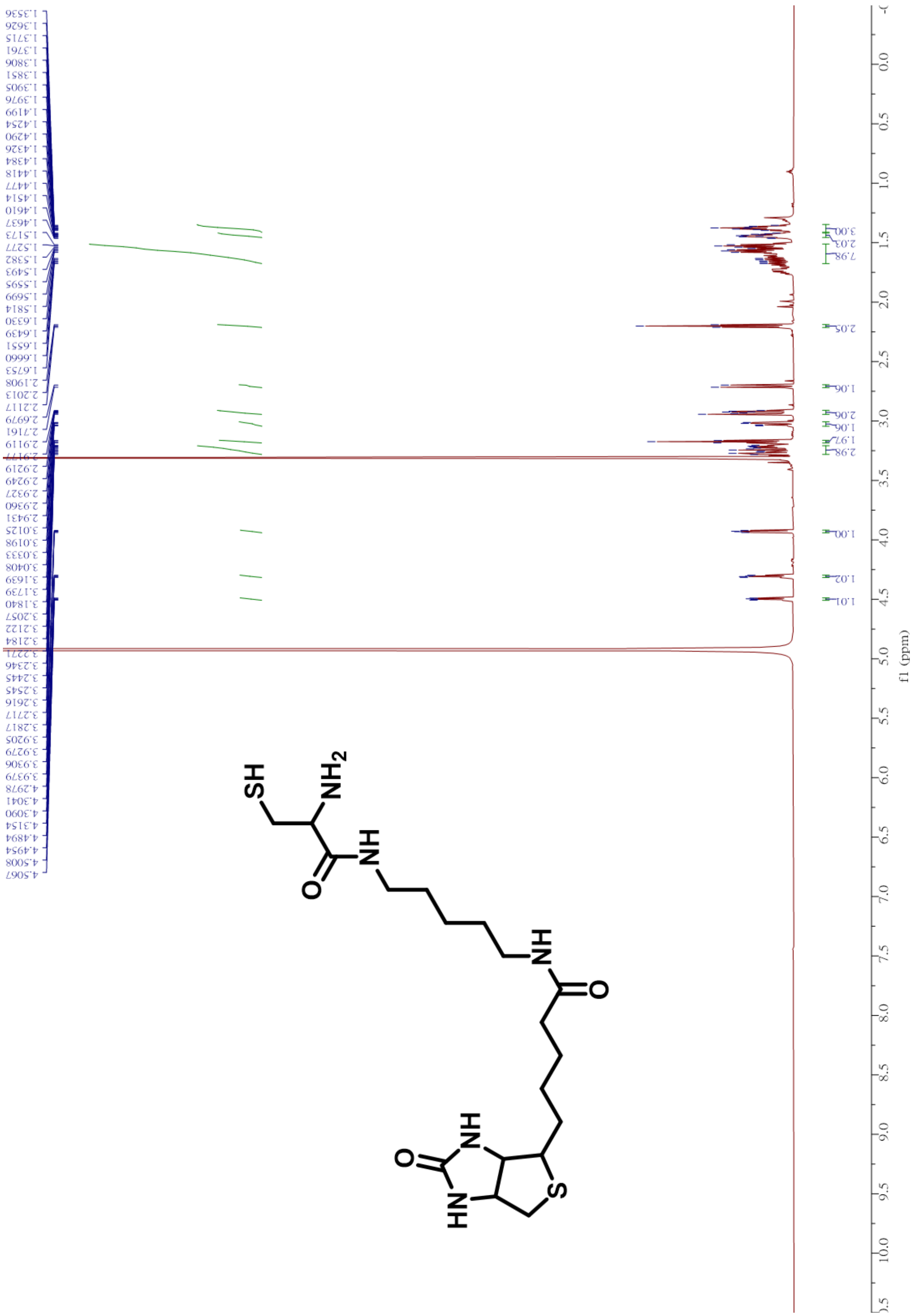
f1 (ppm)

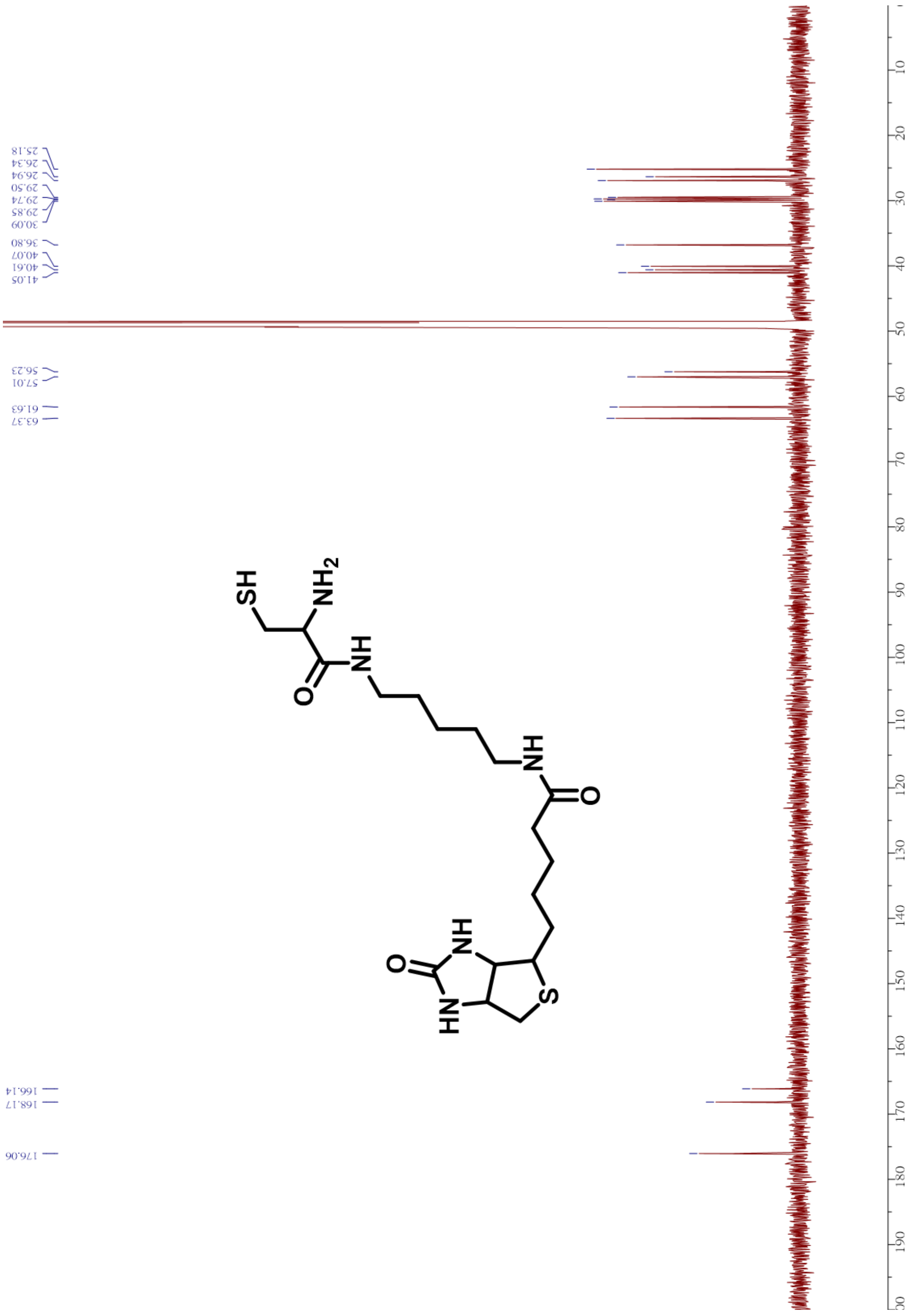
25.18
26.34
26.94
29.50
29.74
29.85
30.09
36.80
40.07
40.61
41.05
56.23
57.01
61.63
63.37
166.14
168.17
176.06
SH
NH2
NH
O
NH
O
NH
O
HN
S

Data:KP29
Comment:
Description:
Ionization Mode:ESI+
History:Average(MS[1] 0.23..0.26)

Acquired:12/1/2023 1:53:37 PM
Operator:AccuTOF
m/z Calibration File:20231107-TFANa_...
Created:12/1/2023 5:01:57 PM
Created by:AccuTOF

Charge number:1
Tolerance:400.00[ppm], 400.00 .. 400....
Unsaturation Number:-300.5 .. 300.0 (...
Element:^{12}C:18 .. 18, ^{1}H:26 .. 34, ^{14}N:5 .. 5, ^{23}Na:0 .. 1, ^{16}O:3 .. 3, ^{32}S:2 .. 2

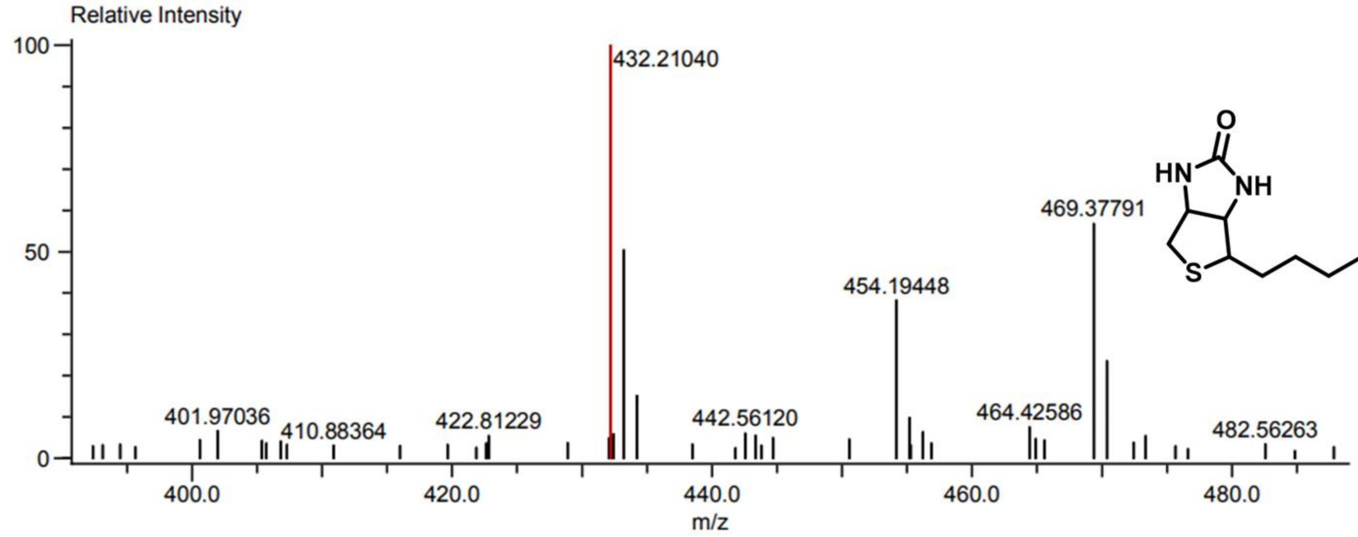

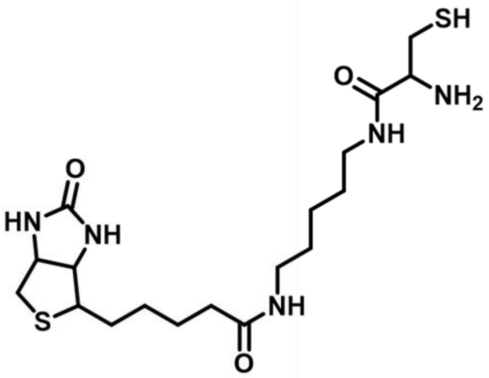

Mass	Intensity	Calc. Mass	Mass Difference [mDa]	Mass Difference [ppm]	Possible Formula
432.21040	3507.07	432.21031	0.09	0.22	$^{12}C_{18}{}^{1}H_{34}{}^{14}N_{5}{}^{16}O_{3}{}^{32}S_{2}$

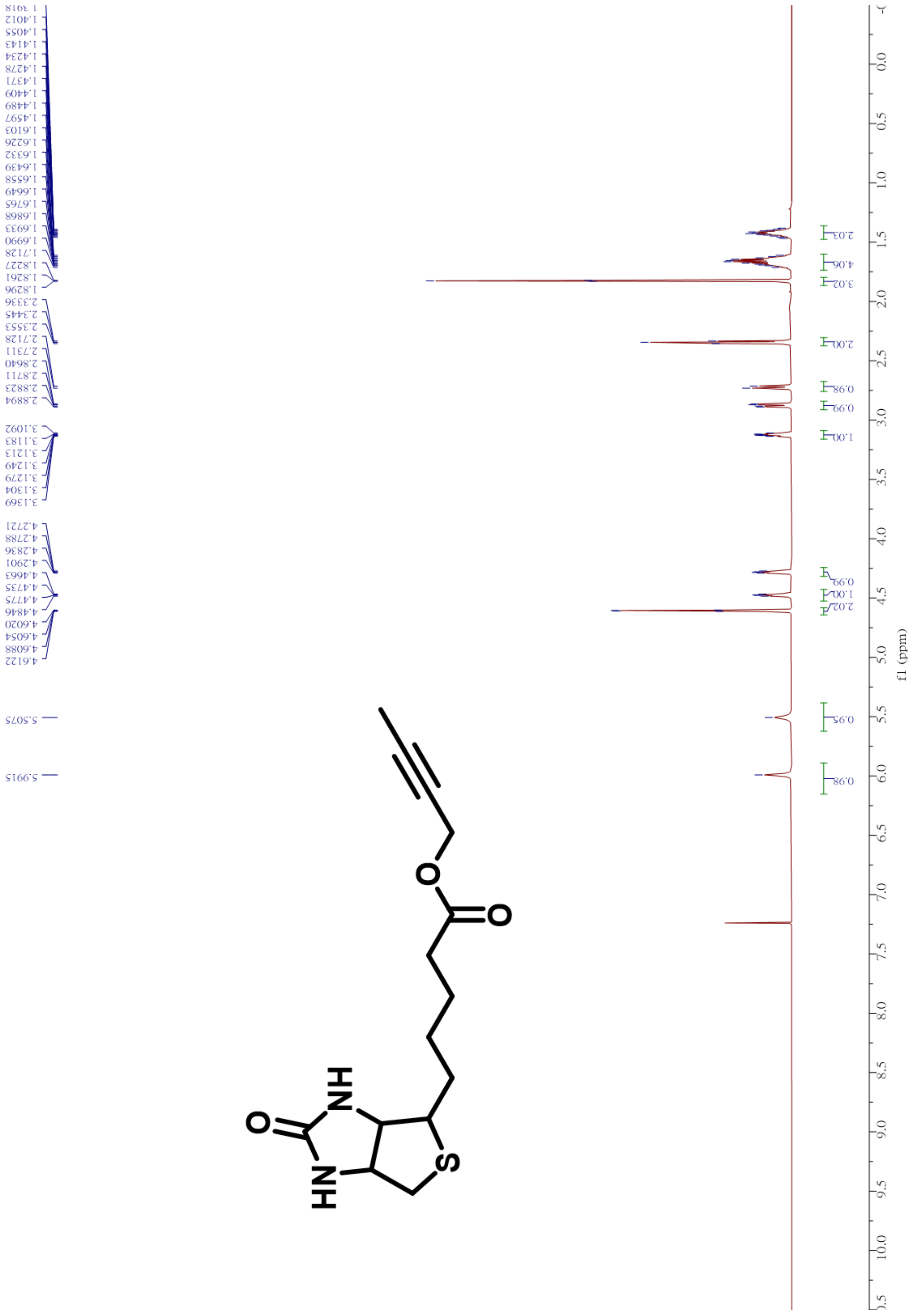
f1 (ppm)

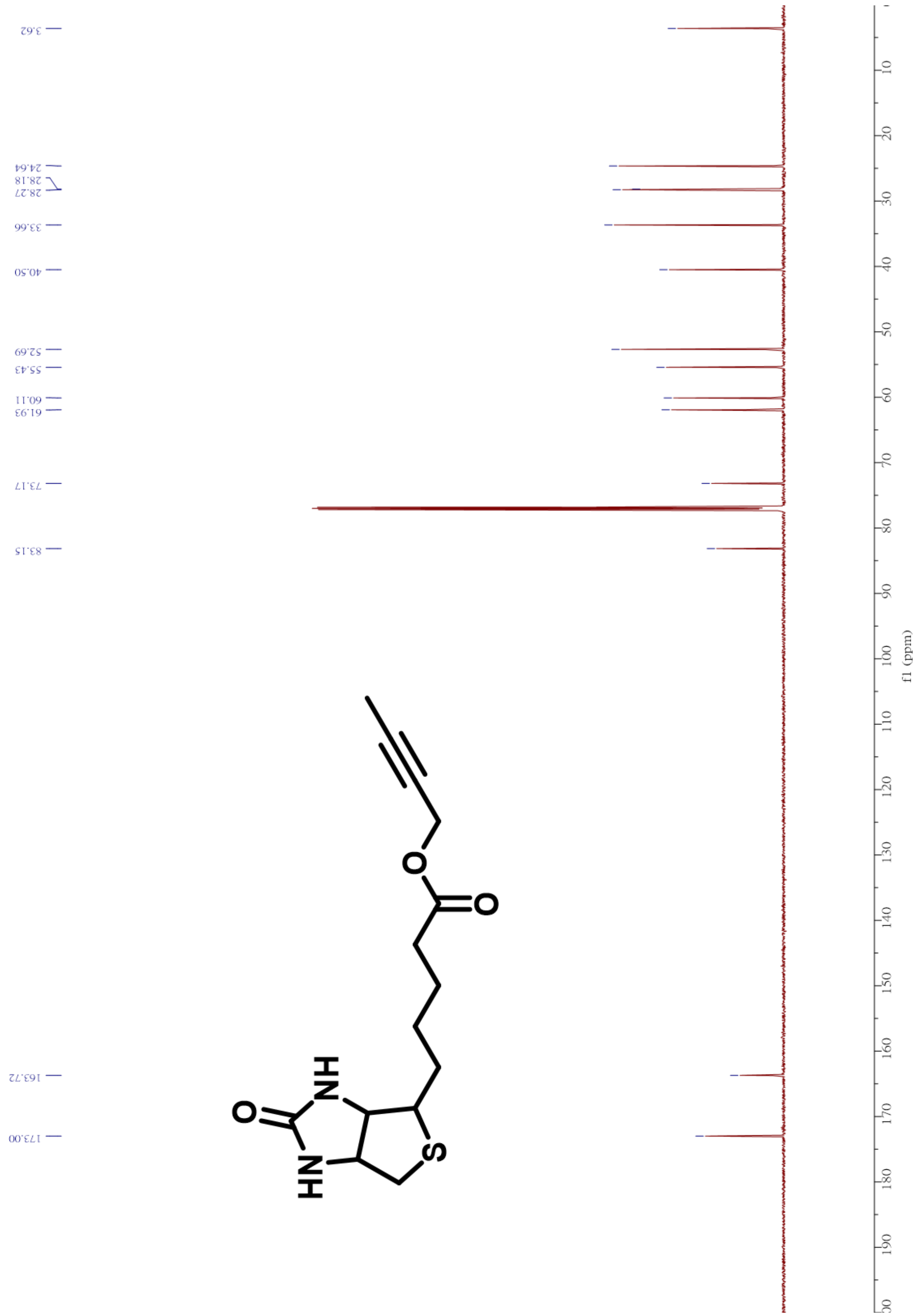
3.62
24.64
28.18
28.27
33.66
40.50
52.69
55.43
60.11
61.93
73.17
83.15
163.72
173.00
f1 (ppm)
HN
NH
O
S
O
O

Data:CY118
Comment:
Description:
Ionization Mode:ESI+
History:Average(MS[1] 0.17..0.18)

Acquired:1/8/2024 11:41:43 AM
Operator:AccuTOF
m/z Calibration File:20231107-TFANa_...
Created:1/8/2024 2:54:33 PM
Created by:AccuTOF

Charge number:1
Tolerance:400.00[ppm], 400.00 .. 400....
Unsaturation Number:-300.5 .. 300.0 (...
Element:^{12}C:14 .. 14, ^{1}H:0 .. 21, ^{14}N:2 .. 2, ^{23}Na:0 .. 3, ^{16}O:3 .. 3, ^{32}S:1 .. 1

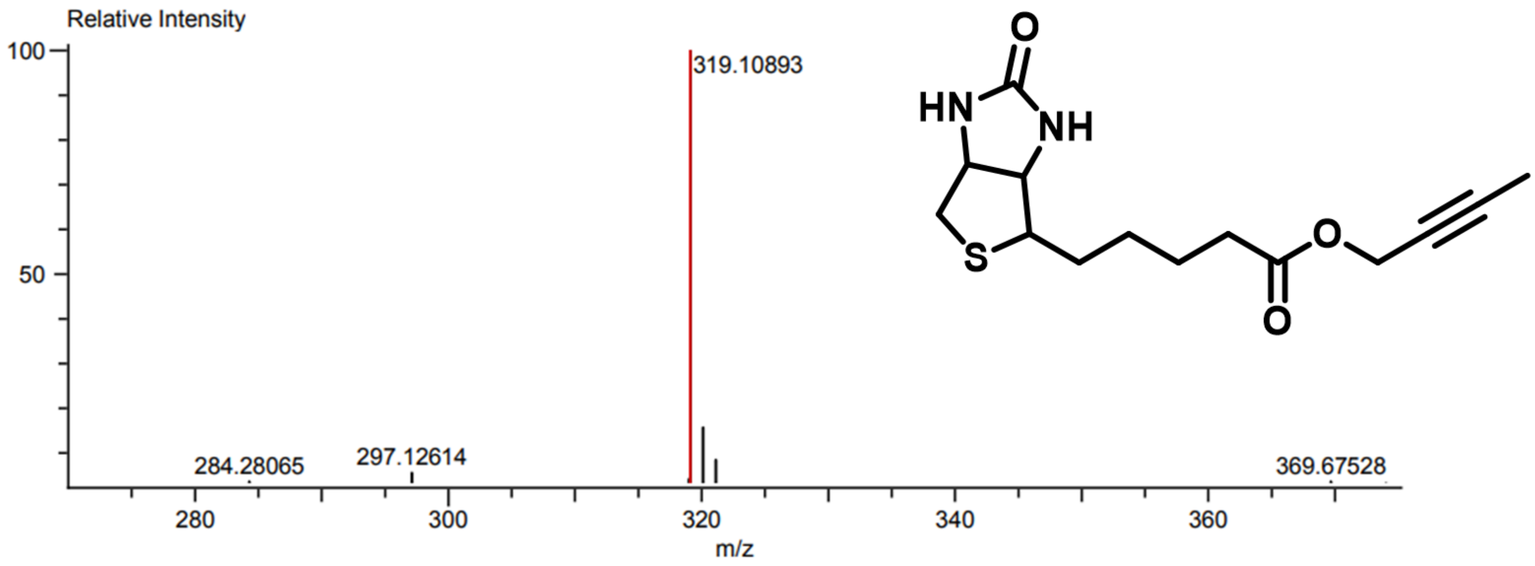

Mass	Intensity	Calc. Mass	Mass Difference [mDa]	Mass Difference [ppm]	Possible Formula
319.10893	17610.25	319.10923	-0.31	-0.96	$^{12}C_{14}{}^{1}H_{20}{}^{14}N_{2}{}^{23}Na_{1}{}^{16}O_{3}{}^{32}S_{1}$

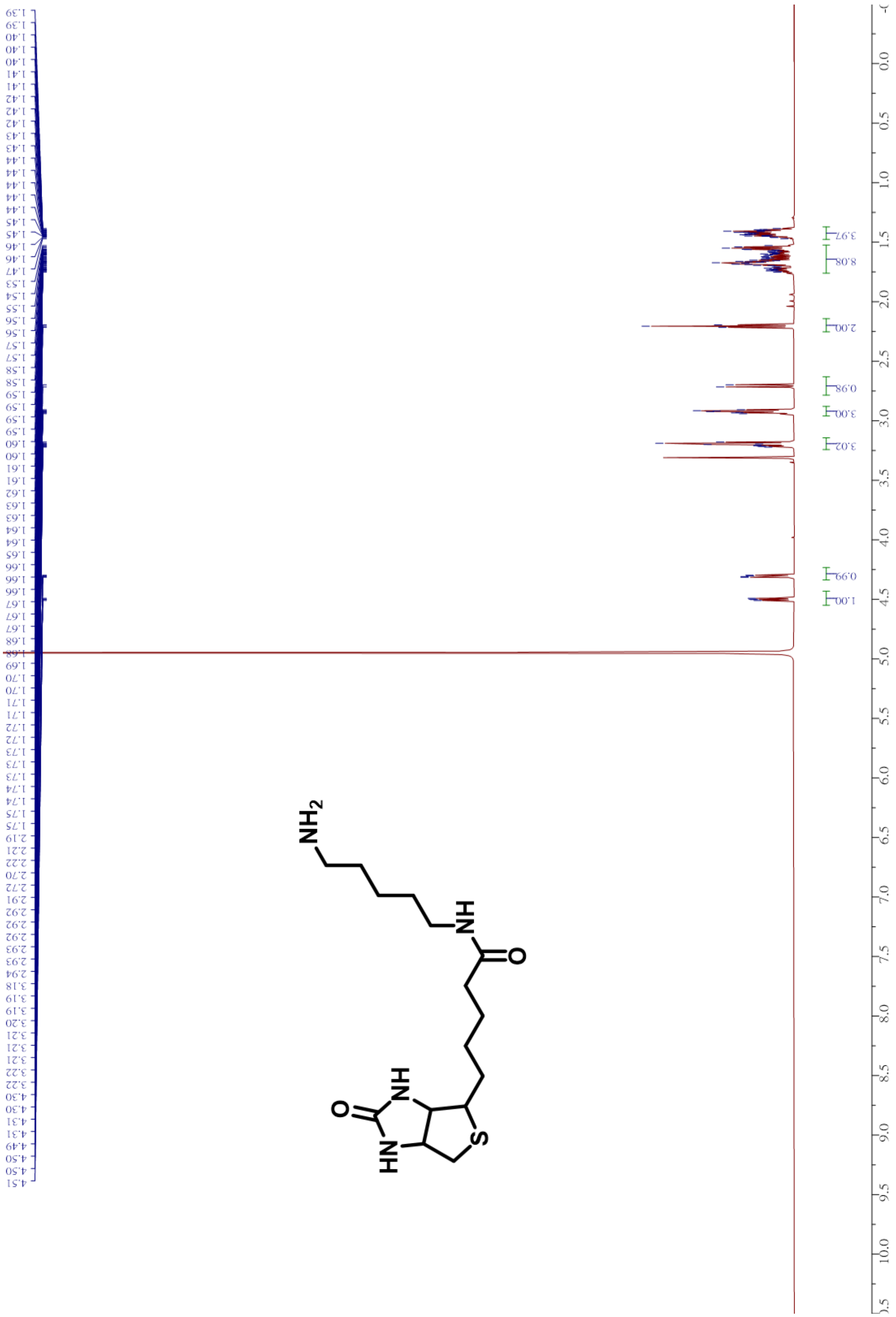
f1 (ppm)

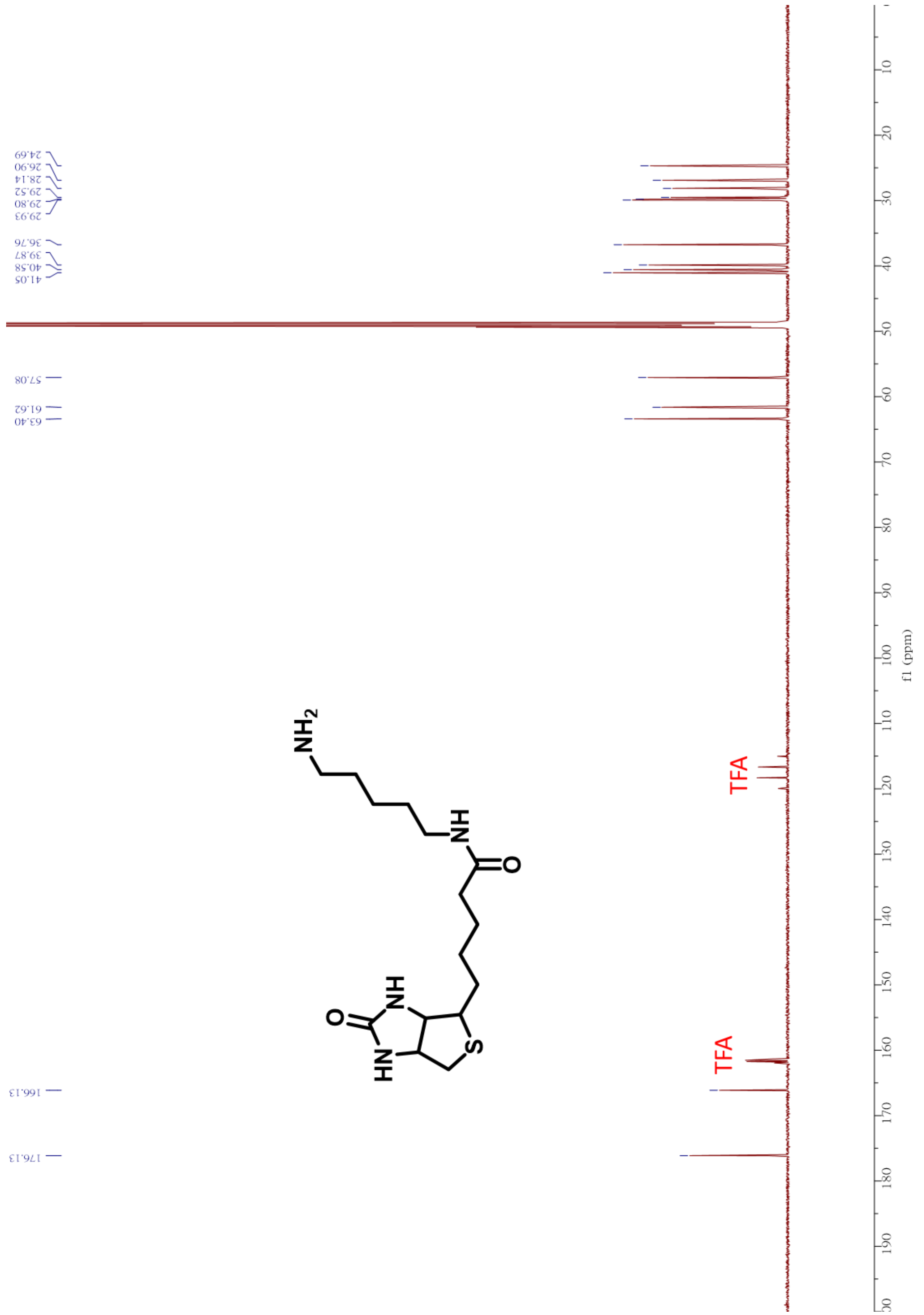
24.69
26.90
28.14
29.52
29.80
29.93
36.76
39.87
40.58
41.05
57.08
61.62
63.40
166.13
176.13
TFA
TFA
f1 (ppm)

Data:CY63
Comment:
Description:
Ionization Mode:ESI+
History:Average(MS[1] 0.26..0.32)

Acquired:11/17/2023 12:07:51 PM
Operator:AccuTOF
m/z Calibration File:20231107-TFANa_...
Created:11/17/2023 2:54:36 PM
Created by:AccuTOF

Charge number:1
Tolerance:400.00[ppm], 400.00 .. 400....
Unsaturation Number:-300.5 .. 300.0 (...
Element:^{12}C:15 .. 15, ^{1}H:29 .. 29, ^{14}N:4 .. 4, ^{23}Na:0 .. 2, ^{16}O:2 .. 2, ^{32}S:1 .. 1

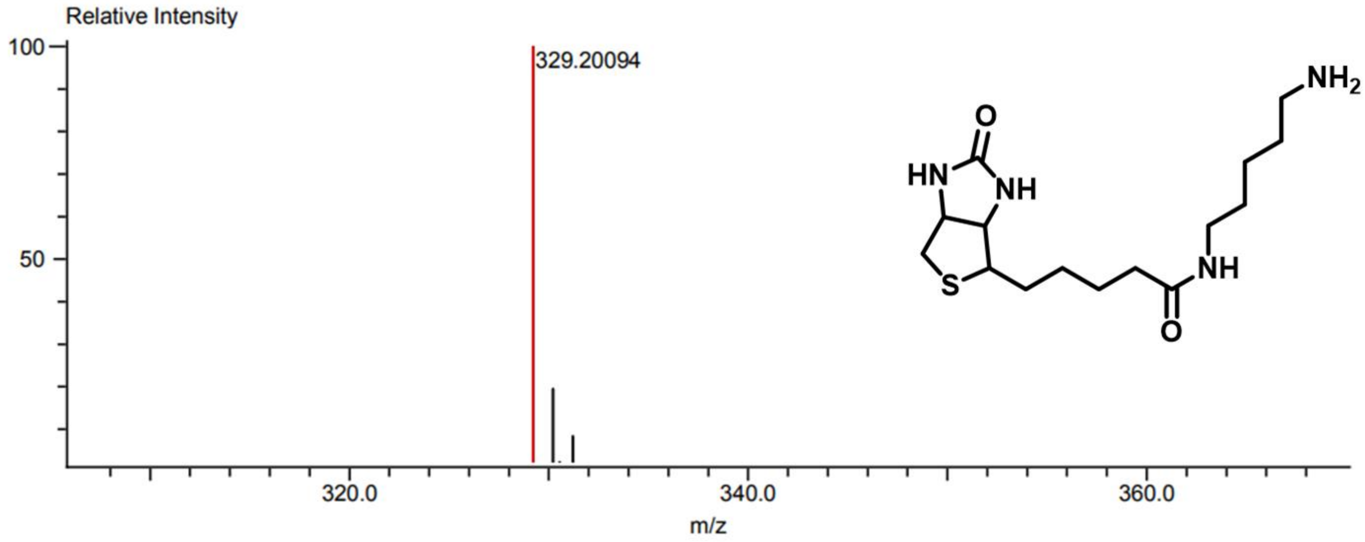

Mass	Intensity	Calc. Mass	Mass Difference [mDa]	Mass Difference [ppm]	Possible Formula
329.20094	206060.81	329.20112	-0.18	-0.55	$^{12}C_{15}{}^{1}H_{29}{}^{14}N_{4}{}^{16}O_{2}{}^{32}S_{1}$

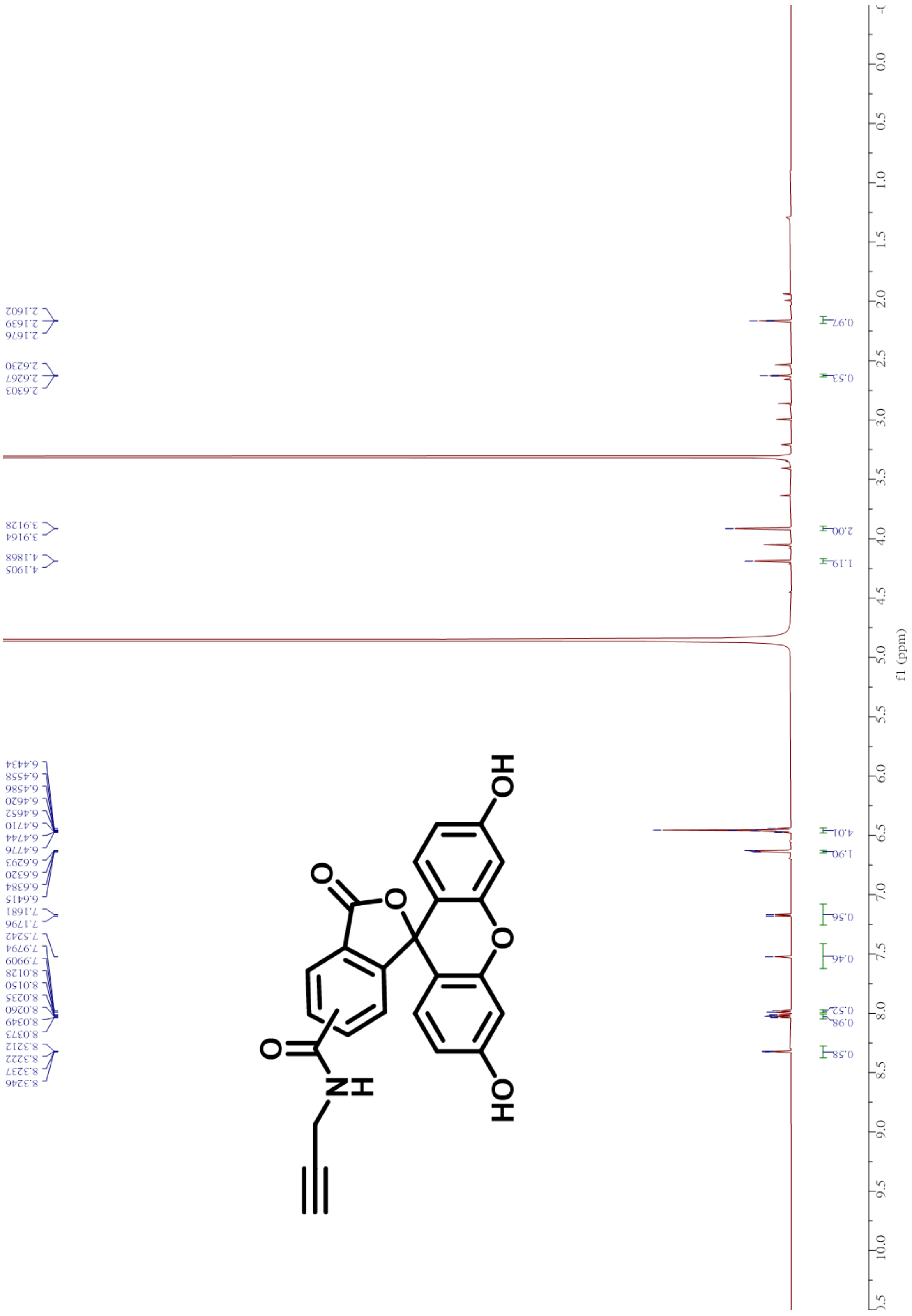

2.1602
2.1639
2.1676
2.6230
2.6267
2.6303
3.9128
3.9164
4.1868
4.1905
6.4434
6.4558
6.4586
6.4620
6.4652
6.4710
6.4744
6.4776
6.6293
6.6320
6.6384
6.6415
7.1681
7.1796
7.5242
7.9794
7.9909
8.0128
8.0150
8.0235
8.0260
8.0349
8.0373
8.3212
8.3222
8.3237
8.3246
0.97
0.53
2.00
1.19
4.01
1.90
0.56
0.46
0.52
0.98
0.58
HO
OH
O
NH
f1 (ppm)

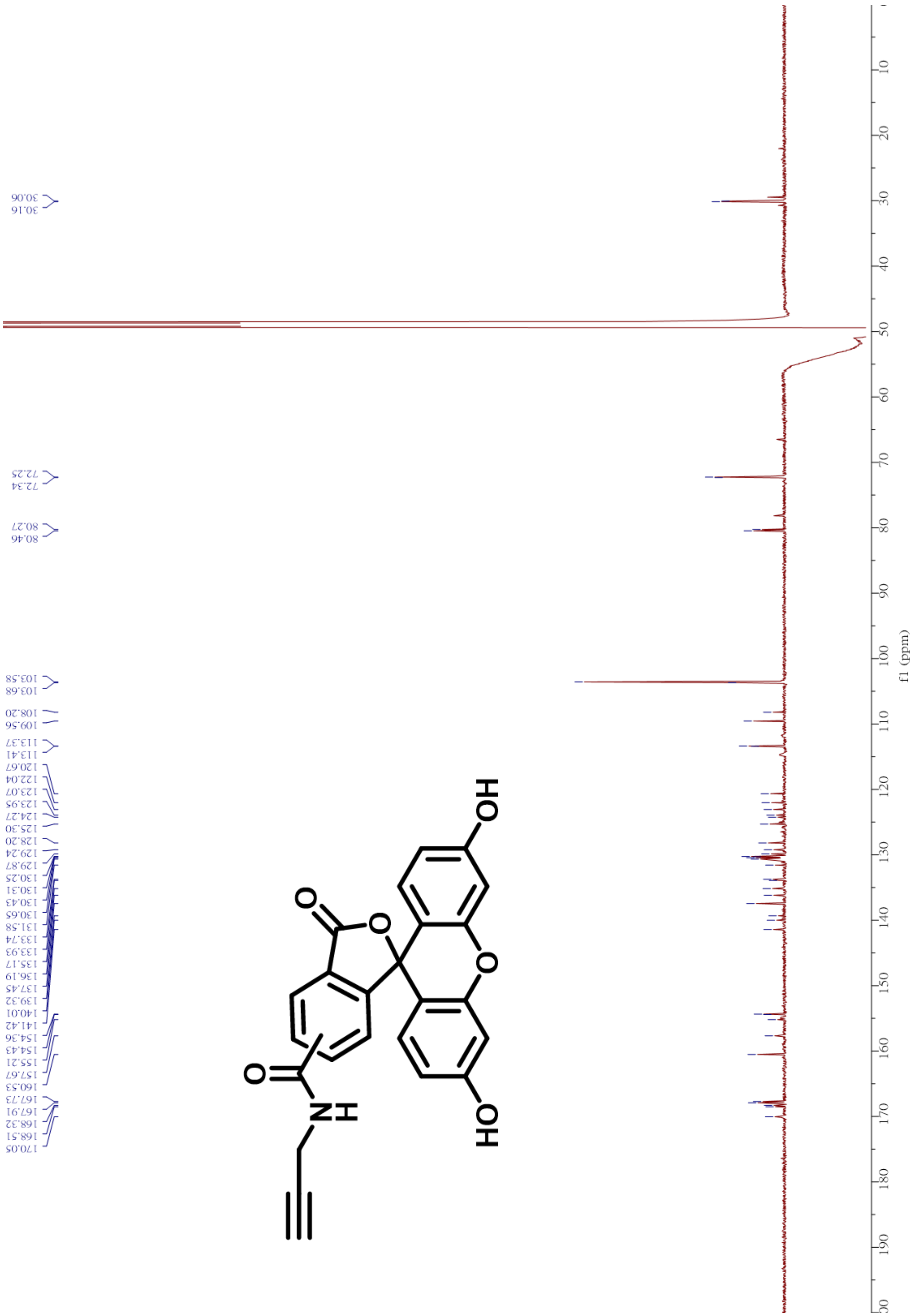
30.06
30.16
72.25
72.34
80.27
80.46
103.58
103.68
108.20
109.56
113.37
113.41
120.67
122.04
123.07
123.95
124.27
125.30
128.20
129.24
129.87
130.25
130.31
130.43
130.65
131.58
133.74
133.93
135.17
136.19
137.45
139.32
140.01
141.42
154.36
154.43
155.21
157.67
160.53
167.73
167.91
168.32
168.51
170.05
f1 (ppm)

Data:KP12-
Comment:
Description:
Ionization Mode:ESI-
History:Average(MS[1] 0.15..0.19)

Acquired:8/18/2023 4:43:08 PM
Operator:AccuTOF
m/z Calibration File:20230608-TFANa_...
Created:8/18/2023 5:15:14 PM
Created by:AccuTOF

Charge number:1
Tolerance:300.00[ppm], 300.00 .. 300....
Unsaturation Number:-200.5 .. 200.0 (...
Element:^{12}C:24 .. 24, ^{1}H:0 .. 14, ^{14}N:1 .. 1, ^{23}Na:0 .. 2, ^{16}O:6 .. 6

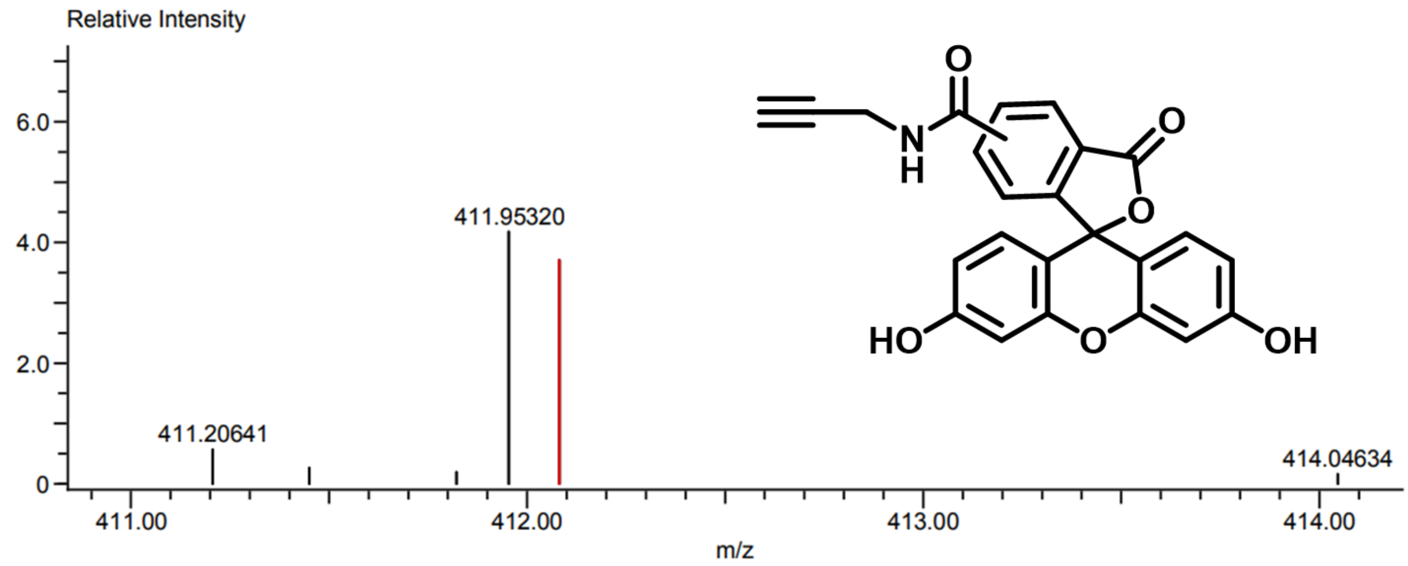

Mass	Intensity	Calc. Mass	Mass Difference [mDa]	Mass Difference [ppm]	Possible Formula
412.08210	1078.35	412.08211	-0.01	-0.02	$^{12}C_{24}{}^{1}H_{14}{}^{14}N_{1}{}^{16}O_{6}$

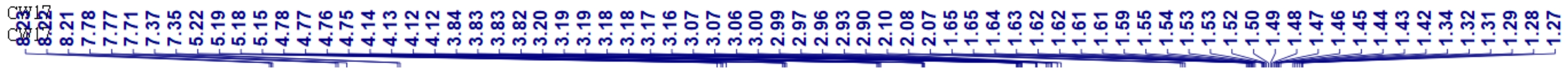

	Parameter	Value
1	Title	CW17
2	Solvent	dmso
3	Temperature	25.0
4	Pulse Sequence	s2pul
5	Experiment	1D
6	Number of Scans	56
7	Relaxation Delay	1.0000
8	Pulse Width	5.8500
9	Presaturation Frequency	
10	Spectrometer Frequency	499.78
11	Spectral Width	7996.8
12	Lowest Frequency	-993.1
13	Nucleus	1H
14	Acquired Size	16384
15	Spectral Size	65536
16	Digital Resolution	0.12

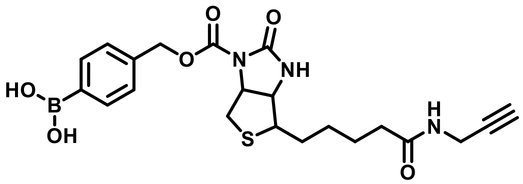

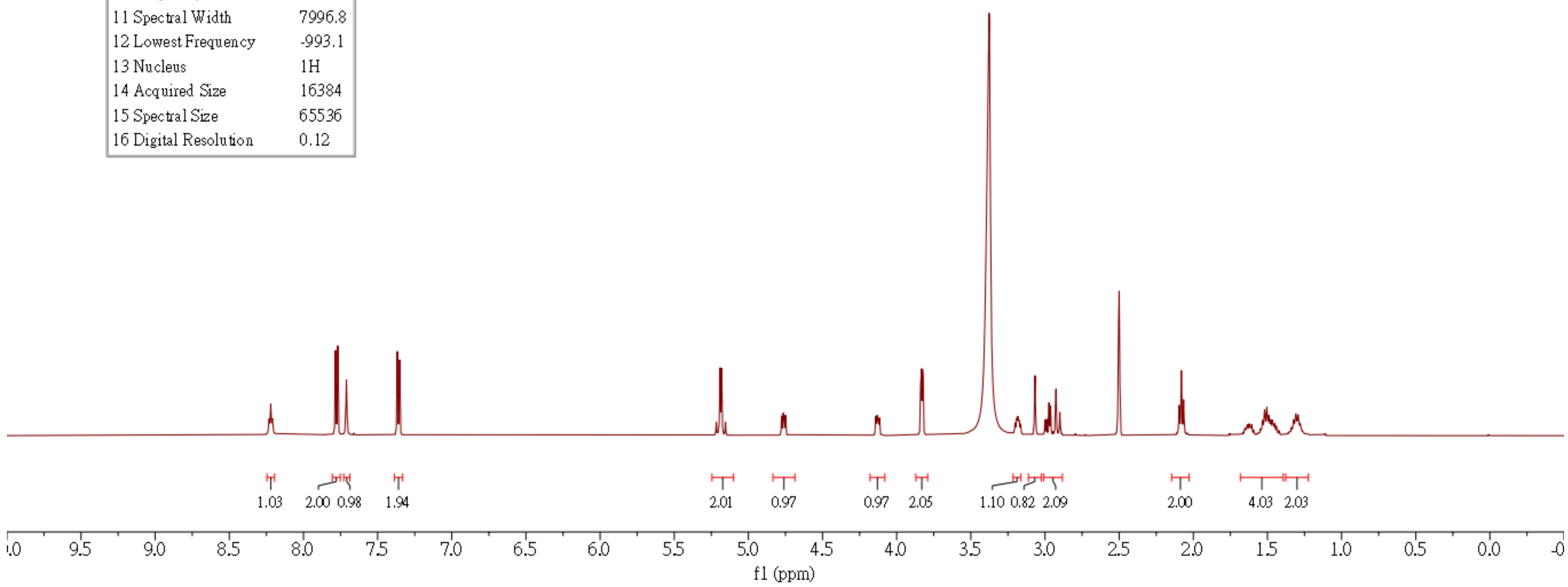

CW17-13C
CW17

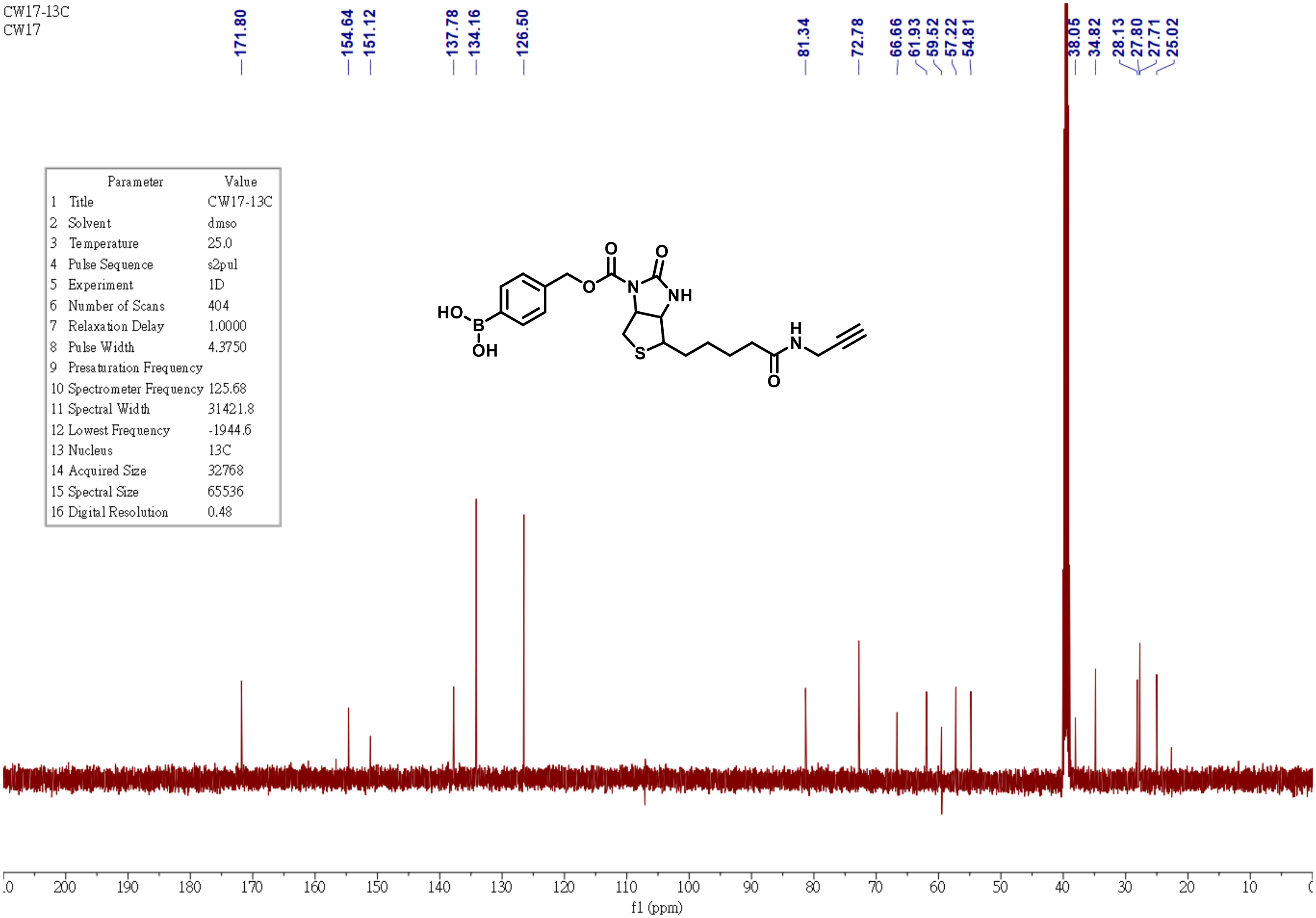

	Parameter	Value
1	Title	CW17-13C
2	Solvent	dmso
3	Temperature	25.0
4	Pulse Sequence	s2pul
5	Experiment	1D
6	Number of Scans	404
7	Relaxation Delay	1.0000
8	Pulse Width	4.3750
9	Presaturation Frequency	
10	Spectrometer Frequency	125.68
11	Spectral Width	31421.8
12	Lowest Frequency	-1944.6
13	Nucleus	13C
14	Acquired Size	32768
15	Spectral Size	65536
16	Digital Resolution	0.48

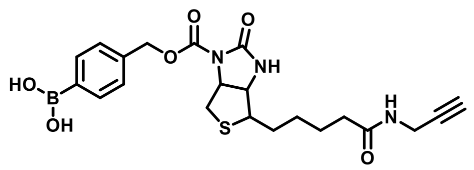

Data:YR55
Comment:
Description:
Ionization Mode:ESI+
History:Average(MS[1] 0.24..0.28)

Acquired:10/2/2023 5:04:33 PM
Operator:AccuTOF
m/z Calibration File:20230914-TFANa_...
Created:10/3/2023 2:57:39 PM
Created by:AccuTOF

Charge number:1
Tolerance:500.00[ppm], 500.00 .. 500....
Unsaturation Number:-300.5 .. 300.0 (...
Element:^{12}C:21 .. 21, ^{1}H:26 .. 29, ^{11}B:1 .. 1, ^{14}N:3 .. 3, ^{23}Na:0 .. 2, ^{16}O:6 .. 6, ^{32}S:1 .. 1

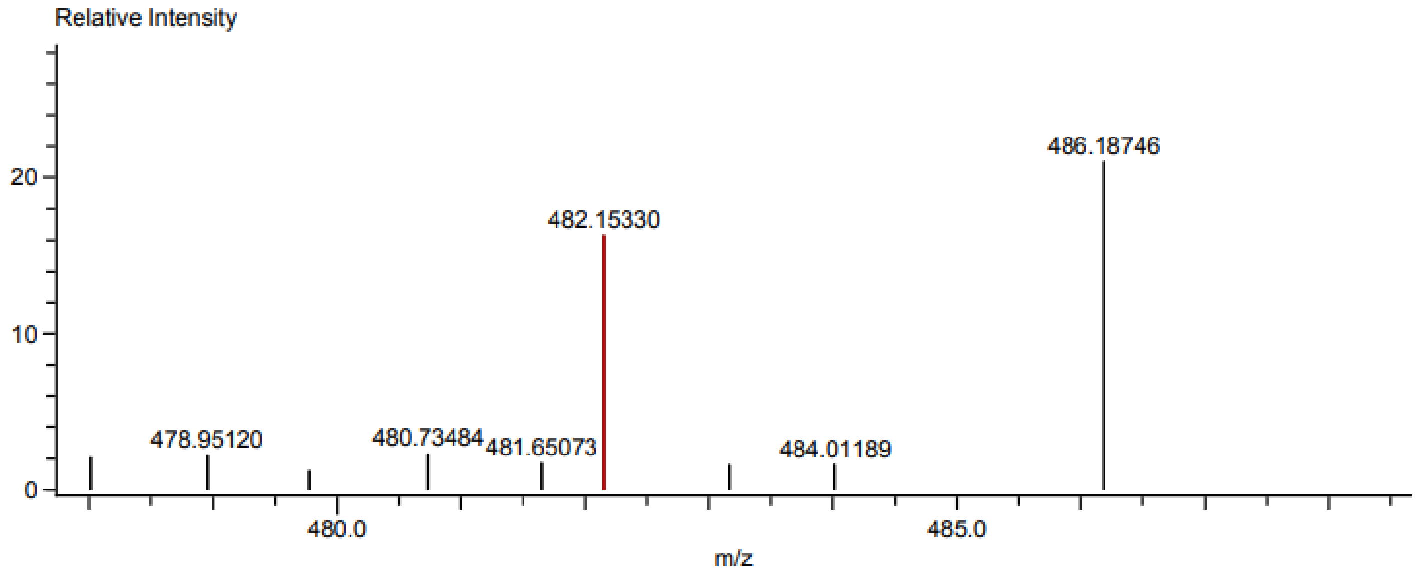

Mass	Intensity	Calc. Mass	Mass Difference [mDa]	Mass Difference [ppm]	Possible Formula
482.15330	1115.45	482.15331	-0.01	-0.01	$^{12}C_{21}{}^{1}H_{26}{}^{11}B_{1}{}^{14}N_{3}{}^{23}Na_{1}{}^{16}O_{6}{}^{32}S_{1}$
